# Hydration of Aliphatic Nitriles Catalyzed by an Osmium Polyhydride: Evidence for an Alternative Mechanism

**DOI:** 10.1021/acs.inorgchem.1c00380

**Published:** 2021-04-27

**Authors:** Juan C. Babón, Miguel A. Esteruelas, Ana M. López, Enrique Oñate

**Affiliations:** Departamento de Química Inorgánica, Instituto de Síntesis Química y Catálisis Homogénea (ISQCH), Centro de Innovación en Química Avanzada (ORFEO-CINQA), Consejo Superior de Investigaciones Científicas (CSIC)—Universidad de Zaragoza, Zaragoza 50009, Spain

## Abstract

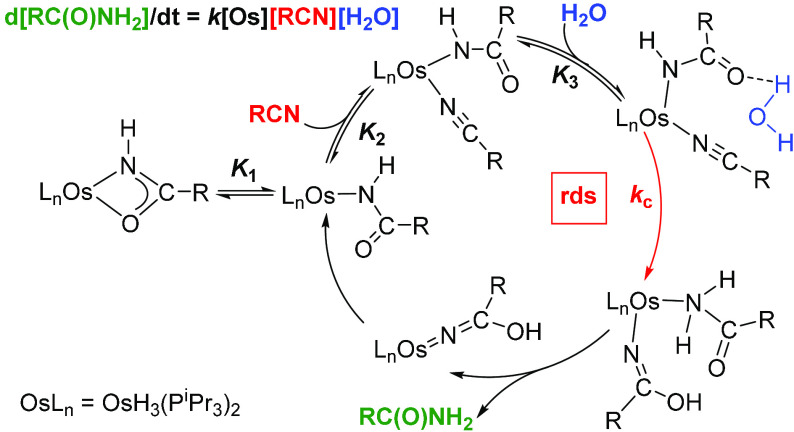

The hexahydride OsH_6_(P^i^Pr_3_)_2_ competently catalyzes the hydration of aliphatic nitriles to amides. The main metal species under the catalytic conditions are the trihydride osmium(IV) amidate derivatives OsH_3_{κ^2^-*N*,*O*-[HNC(O)R]}(P^i^Pr_3_)_2_, which have been isolated and fully characterized for R = ^i^Pr and ^t^Bu. The rate of hydration is proportional to the concentrations of the catalyst precursor, nitrile, and water. When these experimental findings and density functional theory calculations are combined, the mechanism of catalysis has been established. Complexes OsH_3_{κ^2^-*N*,*O*-[HNC(O)R]}(P^i^Pr_3_)_2_ dissociate the carbonyl group of the chelate to afford κ^1^-*N*-amidate derivatives, which coordinate the nitrile. The subsequent attack of an external water molecule to both the C(sp) atom of the nitrile and the N atom of the amidate affords the amide and regenerates the κ^1^-*N*-amidate catalysts. The attack is concerted and takes place through a cyclic six-membered transition state, which involves C_nitrile_···O–H···N_amidate_ interactions. Before the attack, the free carbonyl group of the κ^1^-*N*-amidate ligand fixes the water molecule in the vicinity of the C(sp) atom of the nitrile.

## Abstract 2

The hexahydride complex OsH_6_(P^i^Pr_3_)_2_ competently catalyzes the hydration of aliphatic nitriles to amides. Isolation of the main metal species under the catalytic conditions, kinetics of hydration, and density functional theory calculations support an alternative mechanism to those previously reported.

## Introduction

Amide functional groups are present in natural and synthetic products, including some drugs. In addition, amide compounds find industrial application in the production of detergents, lubricants, or polymers, among other manufactured goods.^1^ Amides have been traditionally prepared by procedures involving carboxylic acids and amines. However, these methods generate large quantities of waste, resulting in an unfavorable environmental profile. As a consequence, alternative approaches are being developed using surrogates of both substrates.^2^ In this context, nitriles have been proven to serve as carboxylic acid alternatives. Thus, several efficient reactions for construction of the amide function have been described starting from them.^3^

Homogeneous catalysts of platinum group metals are particularly efficient for developing atom-economical processes. This fact converts them into one of the most powerful tools of modern selective organic synthesis, being therefore especially relevant from an environmental point of view.^4^ Among the reactions developed for the synthesis of amides, nitrile hydration, which leads to primary amides in an atom-economical manner (eq 1), is one of the most elegant reactions promoted by this class of catalysts. It works under reasonable conditions, presents fine control of subsequent hydrolysis of the product to the carboxylic acid, and exhibits a notable functional group tolerance.^5^
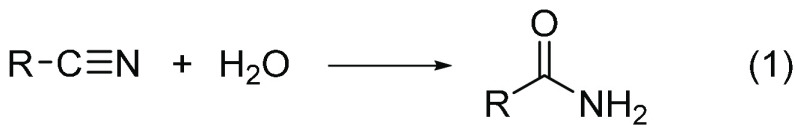
1

**Scheme 1:**
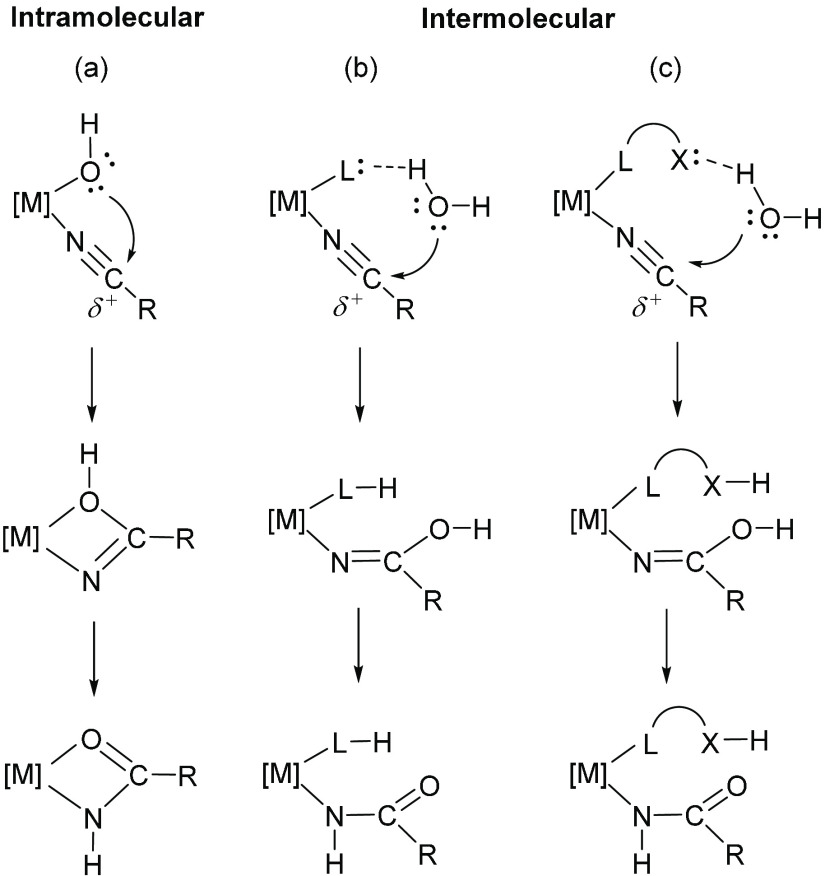
Nucleophilic Attack of the Hydroxide Group to Coordinated Nitriles

Aromatic nitriles have been mainly used in a ratio of about 2:1 with respect to aliphatic ones (Table S1). The reactions have been in an overwhelming preponderance performed in water as the solvent^6^ and, to a lesser extent, in alcohols,^7^ ethers,^8^ or their mixtures with water.^9^ Although complexes of metals of groups 6^6d,10^ and 8–11^6a,6f,6h,6j,7a,9,11^ have proven to be active for nitrile hydration reactions, more than half of the reported catalysts are ruthenium compounds,^6d,6h,6j−6l,7b,7c,8,9a,11b,12^ and the vast majority of them bear specific ligands that enhance the solubility of the complex in water by means of the formation of hydrogen bonds with solvent molecules.^6g,6k,8a,8b,11e,12b,12d,12e,12g,12w^ The improvement of catalysts and reaction conditions have mainly been based on empirical data obtained from trial-and-error methods. Kinetic analysis of the reactions,^6a,13^ isolation of the reaction intermediates,^9a,9e,14^ and a density functional theory (DFT) study of the catalysis^9a,12a,15^—the three legs of the mechanistic investigation—have received scarce attention. As far as we know, mechanistic proposals based on the three legs together have not been reported. There is consensus on the enhancement of the electrophilicity of the C(sp) atom of the nitrile, as a result of coordination to the metal center of the catalyst, which makes it more susceptible to undergoing the nucleophilic attack of the hydroxide group of a water molecule, to form metal amidate intermediates via iminolate species (Scheme 1). The hydroxide attack can be, however, intra- (a) or intermolecular (b and c). In the second case, the water molecule is activated through hydrogen-bonding interaction with a ligand of the metal coordination sphere (b) or a remote heteroatom present in the ligand backbone (c).

Catalysis by complexes of platinum group metals has been traditionally dominated by 4d elements. However, one of the most active and versatile catalysts for nitrile hydration is the platinum complex PtH{(PMe_2_O)_2_H}(PMe_2_OH), reported by Parkins and co-workers in 1995^16^ and improved by Virgil, Grubbs, and co-workers for cyanohydrins in 2018.^17^ Recently, Yao and co-workers have also discovered half-sandwich iridium catalysts, which display excellent activity, under mild conditions, for a broad scope of nitriles.^18^ Osmium is the less used element in catalysis from the six platinum group metals, although it has proven to be particularly useful in the asymmetric dihydroxylation of olefins and reactions similar to that,^19^ some reductions,^20^ C–C^21^ and C–heteroatom^22^ couplings, and acceptorless dehydrogenation of liquid organic hydrogen carriers^23^ and boranes,^24^ whereas complexes [Os(OH)(η^6^-*p*-cymene)IPr]OTf [IPr = 1,3-bis(2,6-diisopropylphenyl)imidazolydene; OTf = CF_3_SO_3_]^25^ and OsCl_2_(η^6^-*p*-cymene)(PMe_2_OH)^26^ promote nitrile hydration in water/2-propanol and water, respectively.

The osmium chemistry is rich in hydride complexes, which are further playing a relevant role in catalysis.^27^ Among them, the d^2^ hexahydride species OsH_6_(P^i^Pr_3_)_2_ (**1**) occupies a prominent place because of its ability to activate σ bonds,^28^ which converts it in one of the keystones in the development of the modern osmium organometallic chemistry. In the search for a catalyst that could work with high efficiency for the hydration of aliphatic nitriles (the least studied) in a conventional organic solvent, we decided to explore its performance. It bears a usual commercially available ligand, particularly useful for mechanistic studies, is easily prepared from OsCl_3_·*x*H_2_O, in two steps, in high yield,^29^ and is much more stable and handy than its ruthenium counterpart, the dihydride bis(dihydrogen) derivative RuH_2_(η^2^-H_2_)_2_(P^i^Pr_3_)_2_.^30^

**Scheme 2:**
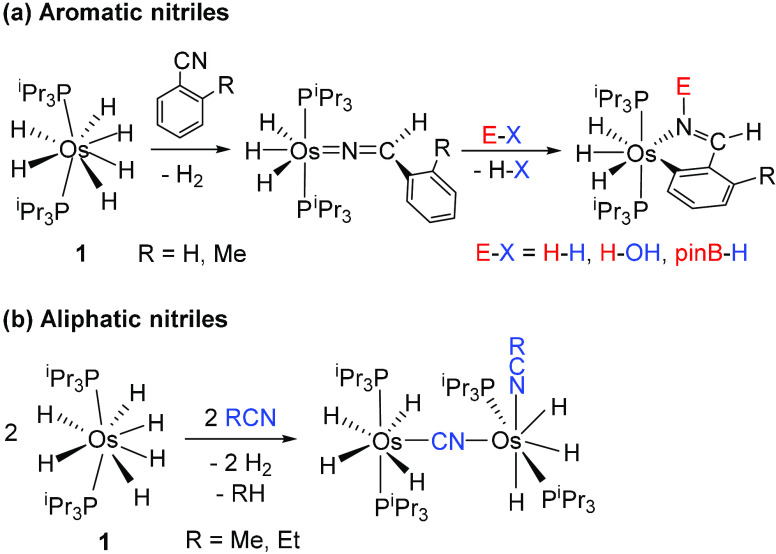
Reactions of Complex **1** with Aromatic and Aliphatic Nitriles

**Scheme 3:**
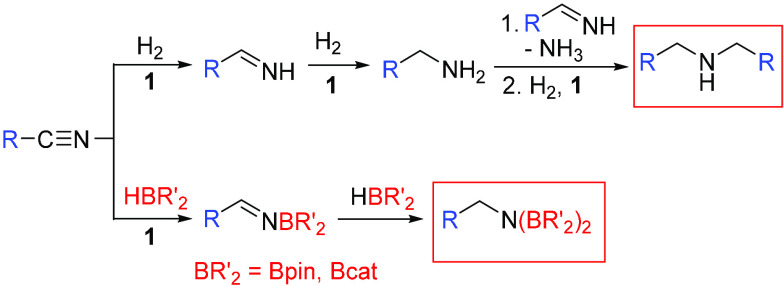
Catalytic Transformations of Aliphatic Nitriles Promoted by Complex **1**

We were inspired by the previous reactivity of complex **1** with nitriles. This polyhydride inserts aromatic nitriles to form trihydride osmium azavinylidene compounds, which activate molecular hydrogen, pinacolborane, and water to give orthometalated phenylaldimine derivatives (Scheme 2a).^31^ In contrast, aliphatic nitriles undergo C(sp)–C(sp^3^) bond activation to yield binuclear complexes (P^i^Pr_3_)_2_H_4_Os(μ-CN)OsH_3_(RCN)(P^i^Pr_3_)_2_ (Scheme 2b).^32^ Under a hydrogen atmosphere or in the presence of boranes, C(sp)–C(sp^3^) bond activation of the aliphatic nitriles is inhibited, and the catalytic formation of secondary amines^33^ and diborylamines^34^ is observed as a consequence of the respective hydrogenation–condensation and dihydroboration of the substrates (Scheme 3). We now show that C(sp)–C(sp^3^) cleavage is also inhibited in the presence of water. In addition, the catalytic formation of aliphatic amides takes place according to eq 1.

This paper reports a catalyst for the hydration of a wide range of aliphatic nitriles, which works with high efficiency under reasonable conditions, and the catalytic mechanism based on kinetic analysis of the catalysis, isolation of the key intermediate, and a DFT study. In addition, it demonstrates that sophisticated ligands favoring the formation of hydrogen bonds with water molecules are not necessary because the true catalysts of the hydration are amidate species generated in situ, under the reaction conditions, and they can generate the hydrogen bonds.

### Reaction Conditions and Scope

Initially, we looked for the optimal reaction conditions to obtain the amides with a high yield in a general manner, using 0.31 M solutions of acetonitrile, in deuterated tetrahydrofuran (THF-*d*_8_) under an argon atmosphere, contained in a NMR tube. Results of the optimization involving the catalyst loading, water amount, and temperature are collected in Table 1.

**Table 1:**
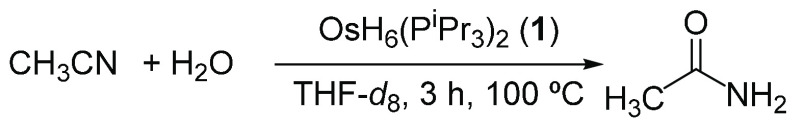
Optimization of the Catalytic Hydration of Aliphatic Nitriles^a^

entry	**1** (mol %)	*T* (°C)	H_2_O (equiv)	yield (%)^b^
1	1	100	20	43
2	2	100	20	52
3	5	100	20	72
4	5	80	20	34
5	5	100	10	55
6	5	100	50	80
7	0	100	50	0

a Reaction conditions: acetonitrile (0.14 mmol) in THF-*d*_8_ (450 μL) for 3 h.

b Yields were calculated by ^1^H NMR spectroscopy using mesitylene as an internal standard.

**Scheme 4:**
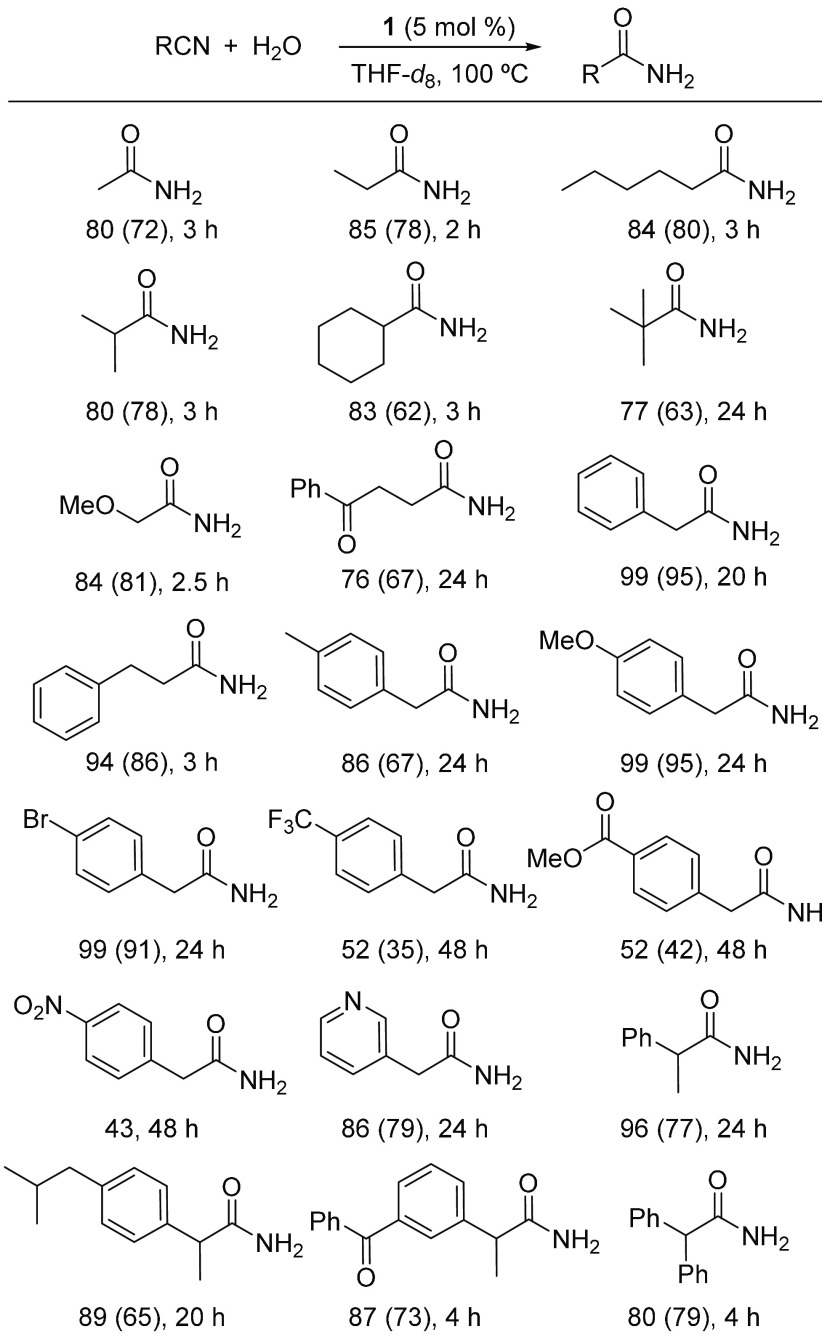
Hydration of Aliphatic Nitriles Catalyzed by **1** Reaction conditions: Corresponding nitrile (0.14 mmol), water (125 μL, 7.0 mmol), **1** (3.6 mg, 0.007 mmol, 5 mol %) in THF-*d*_8_ (450 μL) at 100 °C. Yields were calculated by ^1^H NMR spectroscopy using mesitylene as an internal standard. Isolated yields are in parentheses.

Acetonitrile was transformed in acetamide in 43% yield after 3 h in the presence of 1 mol % complex **1** and 20 equiv of water at 100 °C (entry 1). The raising of the catalyst loading up to 2 mol % increases the yield of the reaction to 52% (entry 2), which undergoes a new increment up to 72% by increasing the amount of catalyst precursor to 5 mol % (entry 3). Lowering the temperature just to 80 °C results in a drastic decrease in the amount of acetamide down to 34% (entry 4). Similarly, reduction of the number of water equivalents to 10 lowers the yield of the reaction to 55% (entry 5), whereas the increment of the water amount up to 50 equiv increases the yield of amide up to 80% (entry 6). Under these conditions, the reaction does not progress in the absence of a catalyst precursor (entry 7). Thus, we decided to carry out the hydration of nitriles under the conditions of entry 6, i.e., using 5 mol % of the hexahydride complex and 50 equiv of water at 100 °C. Under these conditions, the efficiency of complex **1** to promote the hydration of acetonitrile to acetamide is higher than those of the majority of the reported catalysts so far, whereas it compares well with the efficiencies of a few ruthenium precursors^6d,6l,12m,12o,12r,12w^ and the osmium complex OsCl_2_(η^6^-*p*-cymene)(PMe_2_OH),^26^ which work in water as the solvent (Tables S2–S5). Scheme 4 shows the amides isolated under the selected conditions.

Complex **1** displays good tolerance to functional groups. Consequently, it promotes the hydration of a remarkable variety of aliphatic nitriles, including unfunctionalized substrates of linear and branched chains, among others the defiant trisubstituted pivalonitrile, cyclic nitriles as cyclohexanecarbonitrile, and functionalized aliphatic nitriles with methoxide, keto, R-aryl (R = MeO, Br, CF_3_, R, CO_2_Me, NO_2_, and COPh), and pyridyl groups.

The length of the aliphatic chain does not have a noticeable influence on the yield of the obtained amide. Thus, acetamide, propionamide, and hexanamide are formed in similar yields, about 80% after 2–3 h. The hydration is slightly sensitive to the steric hindrance on the C(sp) atom of unfuctionalized substrates; 2-methylpropionitrile and cyclohexanecarbonitrile are converted into the corresponding amides with the same efficiency as that of linear nitriles; however, the trisubstituted pivalonitrile needs 24 h to reach a conversion similar to pivalamide. Although the presence of aromatic substituents at the C_α_ atom with respect to the CN function generally delays the reaction, the corresponding amides are formed in almost quantitative yield after 24 h. In this context, noteworthy is the preparation in high yields of branched chain amide derivatives of ketoprofen and ibuprofen, which are nonsteroidal antiinflammatory drugs widely employed as advanced intermediates in the preparation of several prodrugs and preclinical candidates.^6c,12g^

### Main Species under the Catalytic Conditions

The ^1^H NMR spectra of the catalytic solutions contain a broad triplet at about −13.6 ppm (^2^*J*_H–P_ ≈ 13 Hz), corresponding to a new class of species, in addition to the signals due to the reagents, amide products, and phosphine ligands of the catalyst. The high-field resonance fits with a singlet at about 36 ppm in the ^31^P{^1^H} NMR spectra. Resonances due to **1** are not observed. The new species are rapidly and quantitatively formed and remain while the nitrile is present in the solution and also once it is consumed. To gain information about their nature, we decided to prepare them at a Schlenk tube scale, with two model nitriles: 2-methylpropanenitrile and pivalonitrile. The treatment of THF solutions of **1** with 2.0 equiv of the nitriles and 2.0 equiv of water at 100 °C for 3 h afforded 1.0 equiv of the corresponding amide and the trihydride osmium(IV) amidate derivatives OsH_3_{κ^2^-*N*,*O*-[HNC(O)R]}(P^i^Pr_3_)_2_ [R = ^i^Pr (**2a**), ^t^Bu (**2b**)], according to eq 2. These compounds were isolated as a colorless oil (**2a**) and colorless crystals suitable for X-ray diffraction analysis (**2b**).
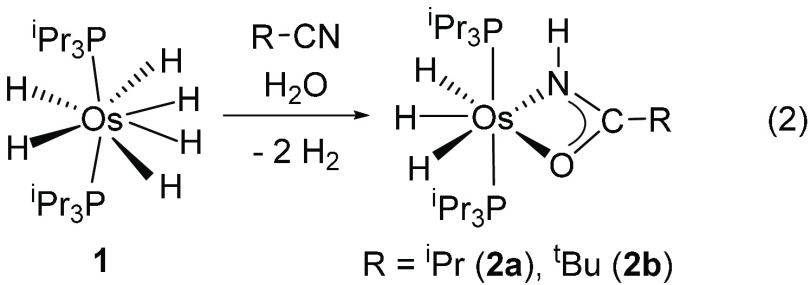
2

**Figure 1:**
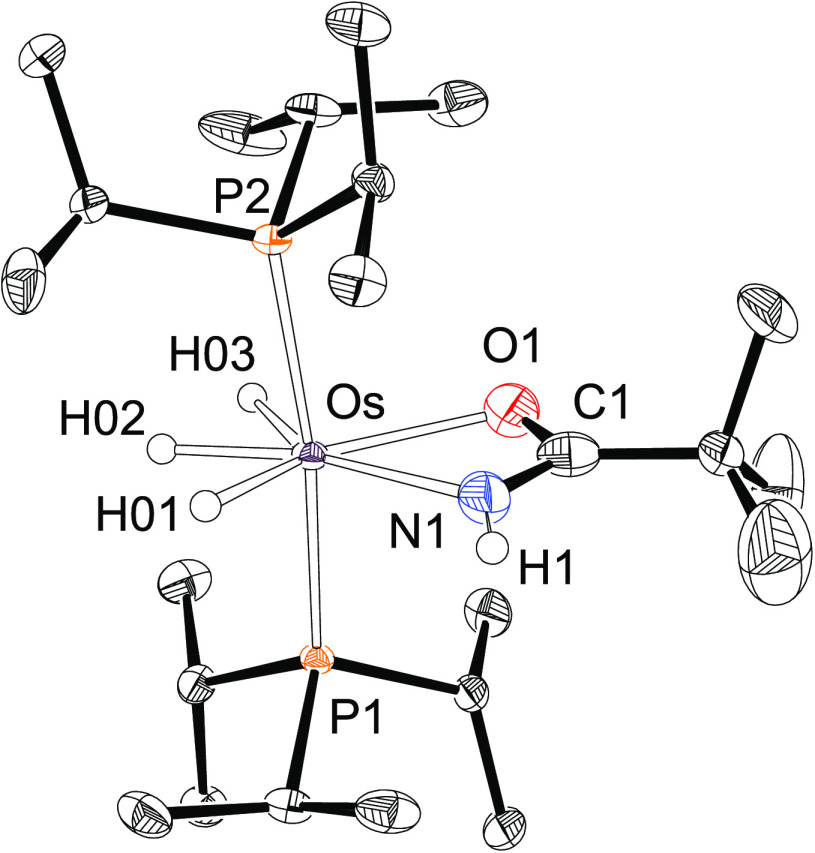
Molecular structure of **2b** with ellipsoids at the 50% probability level. H atoms are omitted for clarity (except for the hydride ligands and NH group). Selected bond distances (Å) and angles (deg): Os–N1 = 2.185(3), Os–O1 = 2.245(2), O1–C1 = 1.289(4), N1–C1 = 1.285(4), Os–P1 = 2.3373(6), Os–P2 = 2.3372(6); N1–Os–O1 = 57.47(10), P1–Os–P2 = 171.79(2).

Figure 1 shows a view of **2b**. The structure proves the formation of the amidate group, which acts as a N,O-chelate ligand with a bite angle of 57.47(10)°. The polyhedron around the metal center is the expected pentagonal bipyramid for a seven-coordinated d^4^ derivative, with the phosphine ligands occupying axial positions [P–Os–P = 171.79(2)°], whereas the chelate and hydride ligands lie at the perpendicular plane. The ^1^H and ^31^P{^1^H} NMR spectra of **2a** and **2b** are consistent with the spectra of the respective catalytic solutions involving 2-methylpropanenitrile and pivalonitrile. Furthermore, the ^1^H NMR spectra of these compounds in toluene-*d*_8_ as a function of the temperature reveal that the hydride ligands undergo a thermally activated position site exchange process, typical for OsH_3_(XY)(P^i^Pr_3_)_2_ complexes.^28f,28g,31^ Thus, the hydride resonance at about −13.6 ppm splits into three signals at about −10, −14, and −15 ppm at temperatures lower than 213 K. In the ^13^C{^1^H} NMR spectra, the presence of the amidate ligand is strongly supported by a singlet close to 181 ppm.

**Figure 2:**
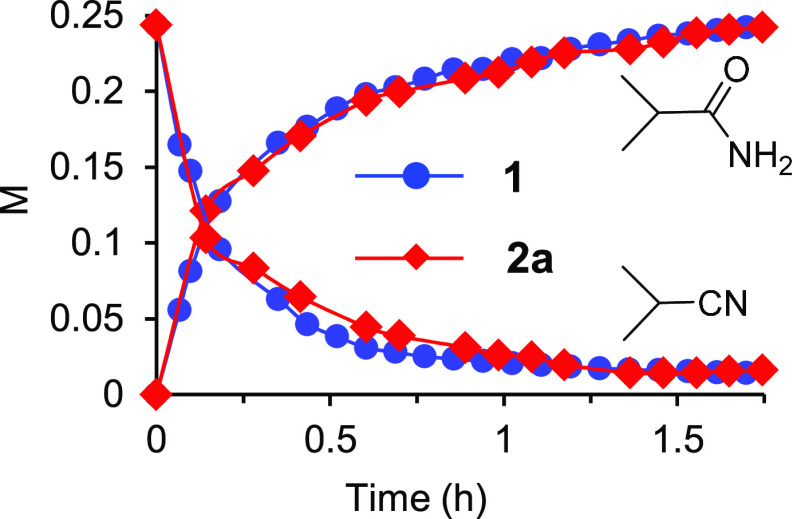
Hydration of 2-methylpropanenitrile (0.24 M) catalyzed by **1** (blue ●) or **2a** (red ◆) (both 1.2 × 10^–2^ M) in THF-*d*_8_ at 100 °C.

Once the nature of the main metal species was established under the catalytic conditions, we investigated their catalytic performance. Thus, hydration of 2-methylpropanenitrile and pivalonitrile was carried out using the isolated complexes **2a** and **2b**, respectively, as catalysts. Figure 2 shows the course of the hydration of 2-methylpropanenitrile in the presence of **1** and **2a**. According to the observed reaction profiles, it is clear that both compounds display the same activity; i.e., under the catalytic conditions, complex **1** reacts with 1.0 equiv of nitrile and 1.0 equiv of water to give trihydride osmium(IV) amidate species, such as **2a** and **2b**, and to release two hydrogen molecules. The formed osmium(IV) amidate compounds are catalyst precursors closer to the true catalyst of hydration than **1**. Each hydration has a specific catalyst that is generated with the nitrile substrate itself.

**Scheme 5:**
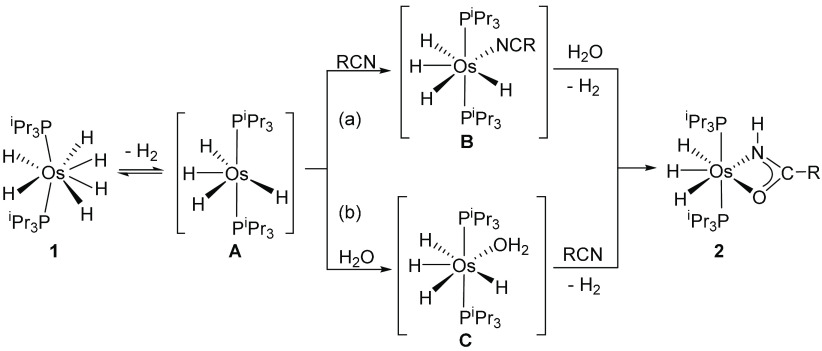
Possible Routes for the Formation of Amidate Complexes

Complex **1** is saturated, and, consequently, its transformation into amidate derivatives needs the previous creation of a coordination vacancy, which occurs by the dissociation of a hydrogen molecule. The resulting unsaturated tetrahydride OsH_4_(P^i^Pr_3_)_2_ (**A**) has been trapped by several types of Lewis bases.^28e,31−35^ Once **A** is generated, the formation of amidate complexes could take place via two different paths: (a) nitrile or (b) water (Scheme 5). The first route should involve the initial coordination of the nitrile to the unsaturated metal center of **A**. The coordination would give **B**, with the coordinated substrate activated for the attack of an external water molecule. The attack should afford the amidate ligand and the release of a second hydrogen molecule. In the second one, the tetrahydride **A** would be trapped by a water molecule. Then, the subsequent reaction of the resulting intermediate **C** with the nitrile could yield the amidate complexes and the second hydrogen molecule. To gain information on the intimate details of the routes and to compare their energetic cost, we carried out DFT calculations at the dispersion-corrected PCM(THF)-B3LYP-D3//SDD(f)-6-31G** level (see computational details in the Supporting Information) using propionitrile as a model of the substrate. The variations in free energy (Δ*G*) were calculated in THF at 298.15 K and 1 atm.

A nitrile route was previously proposed by Lin, Lau, and co-workers to rationalize the hydration of nitriles with an indenylruthenium hydride catalyst. The presence of a Ru–H···H–OH dihydrogen-bonding interaction in the transition state lowers the barrier for nucleophilic attack of an external water molecule to the coordinated nitrile.^12a^ Although the drop is significant (19.3 kcal mol^–1^), the barrier remains too high (40.0 kcal mol^–1^). A similar attack involving an Os–H···H–OH dihydrogen bonding is also possible in our case (Figure S93); the activation energy is even lower than that for the half-sandwich ruthenium catalyst. However, it is still very high (32.5 kcal mol^–1^). Thus, we discarded the nitrile route as a feasible pathway for the formation of amidate compounds.

**Figure 3:**
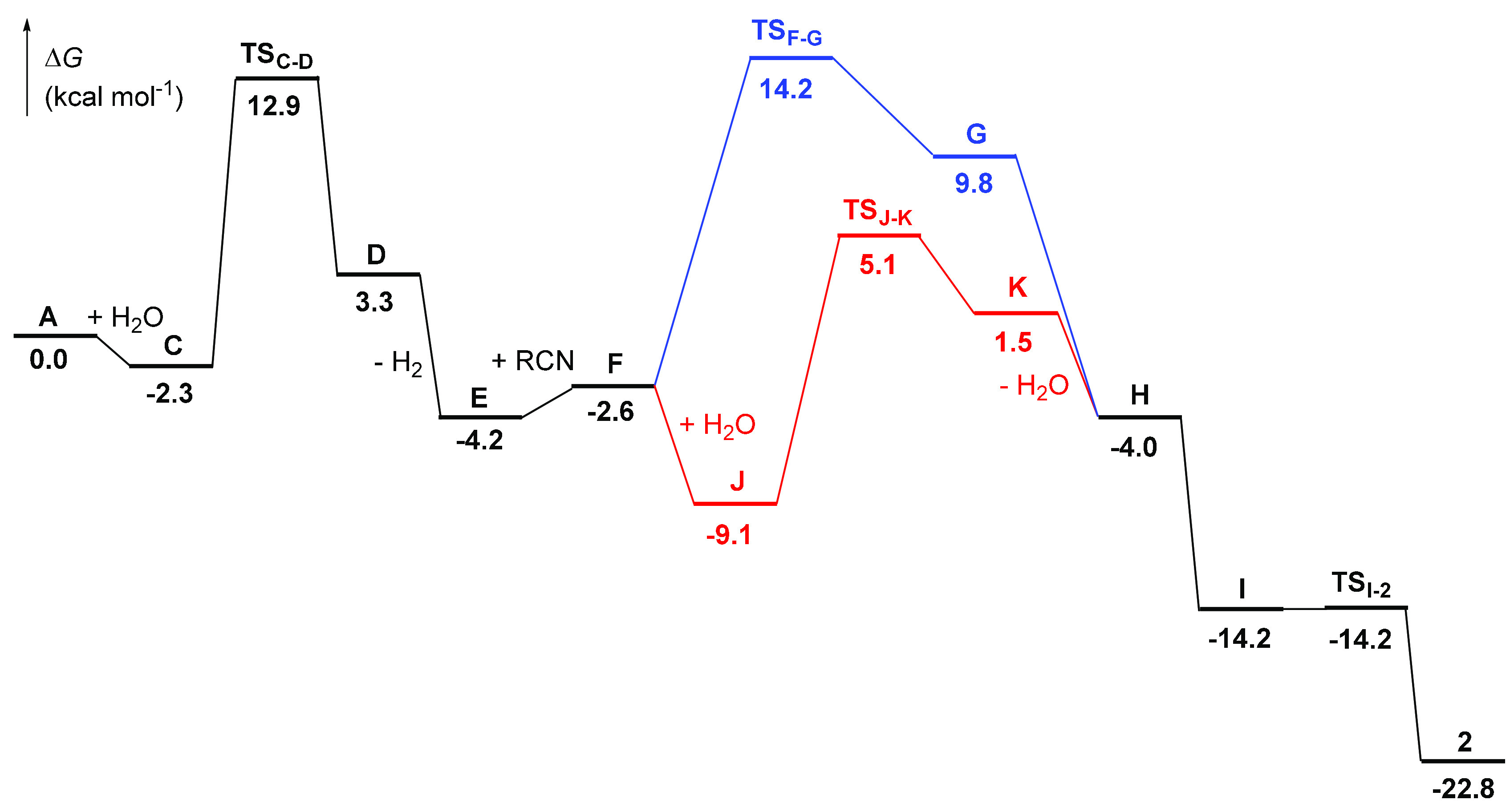
Relative Gibbs energies for formation of the κ^2^-amidate OsH_3_{κ^2^-*N*,*O*-[HNC(O)R]}(P^i^Pr_3_)_2_ (**2**; R = Et) via intramolecular (blue lines) or intermolecular (red lines) attack.

**Scheme 6:**
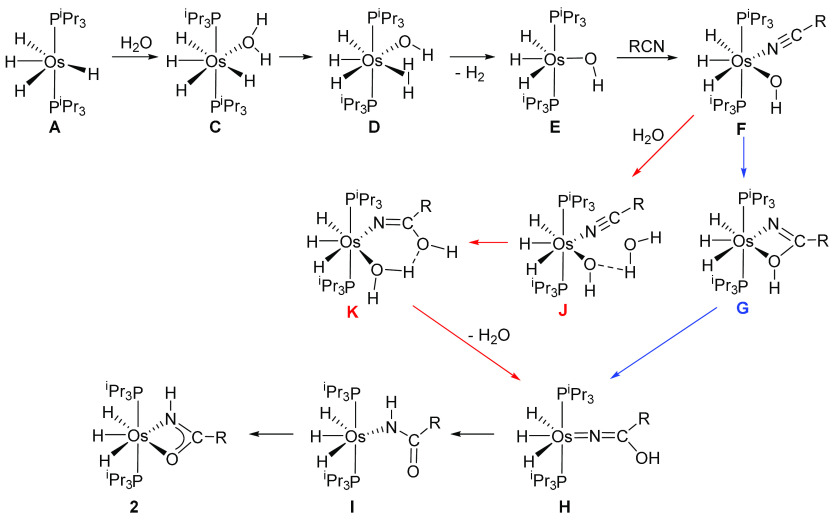
Intermediates in the Formation of κ^2^-Amidate Complexes **2**

Once the nitrile route was discarded, we analyzed the water route. Figure 3 shows the energetic profile of the transformation, whereas Scheme 6 collects the calculated reaction intermediates. Coordination of the water molecule to the metal center of **A** to give **C** is slightly exergonic (2.3 kcal mol^–1^). The formation of **C** is the previous step to the hydride-mediated heterolytic activation of the water molecule. The cleavage occurs with an activation energy of 12.9 kcal mol^–1^, with respect to **A**, and leads to the trihydride hydroxo Kubas-type dihydrogen osmium(IV) species **D** (*d*_H–H_ = 0.839 Å). Subsequent dissociation of the coordinated hydrogen molecule affords the unsaturated six-coordinate osmium(IV) derivative **E**, which lies 4.2 kcal mol^–1^ below **A**. Although hydride hydroxo derivatives of the platinum group metals are very rare and their chemistry is underdeveloped,^36^ the trihydride hydroxoosmium(IV) complex OsH_3_(OH){xant(P^i^Pr_2_)_2_} [xant(P^i^Pr_2_)_2_ = 9,9-dimethyl-4,5-bis(diisopropylphosphino)xanthene], related to **E**, was recently reported and a part of its reactivity studied.^23g,37^ Intermediate **E** displays the typical structure with *C*_*s*_ symmetry of complexes OsH_3_X(PR_3_)_2_. In order to be diamagnetic, these compounds undergo distortion from the octahedral geometry, which involves destabilization of an orbital of the t_2g_ set and the simultaneous stabilization of some occupied ones. This distortion partially cancels the electron deficiency of the metal center, which receives electron density through σ bonds with the hydride ligands and from a lone pair of X via a π bond.^38^ In agreement with the partially saturated character of the metal center of **E**, coordination of the nitrile is slightly endergonic (1.6 kcal mol^–1^). The resulting seven-coordinate species **F** has two pathways to evolve into the amidate complex, one intramolecular and the other intermolecular. The former would involve the attack of the coordinated hydroxo group to the C(sp) atom of the nitrile, while in the second one, the attack should proceed from the hydroxo group of an external water molecule. The intramolecular attack has to overcome an activation energy of 14.2 kcal mol^–1^ with respect to **A**, which is experimentally accessible, and leads to the κ^2^-iminolate derivative **G**. Dissociation of the coordinated OH group of the iminolate affords the hydroxoazavinylidene species **H**, a thermodynamically disfavored tautomer of the κ^1^-*N*-amidate **I**. Coordination of the carbonyl group of the amidate ligand of the latter yields the experimentally observed κ^2^-amidate species **2** in an exergonic overall process by 22.8 kcal mol^–1^ with respect to **A**. The barrier for the intermolecular attack is lower than that for the intramolecular one (5.1 kcal mol^–1^ with regard to **A**). The reason is that the external water molecule forms a HO···H–OH hydrogen bond with the coordinated hydroxo group, which provides slight stabilization of the system. The resulting adduct **J** lies 9.1 kcal mol^–1^ below **A**. The attack leads to the κ^1^-*N*-iminolate **K**, which coordinates a water ligand. Its dissociation regenerates the external water molecule and affords the hydroxoazavinylidene intermediate **H**, a common intermediate for both pathways. A comparison of the overall profile for both routes reveals that the main difference between them is the rate-determining step of the process. While, for the intramolecular pathway, it is the attack of the coordinated hydroxo group at the nitrile, in the intermolecular one, it is the heterolytic activation of the water molecule. The difference between the barriers (ΔΔ*G*^⧧^) is small, 1.3 kcal mol^–1^, and both barriers are low and experimentally accessible.

### Kinetics and Mechanism of the Catalysis

Once the main metal species under the hydration conditions was established and its method of generation was analyzed, we investigated the mechanism of catalysis. To this end, the kinetics of hydration of 2-methylpropanenitrile promoted by **1** was studied in THF-*d*_8_ under pseudo-first-order conditions. The reactions were followed by ^1^H NMR spectroscopy and carried out in the 373–348 K temperature range with concentrations of the catalyst precursor **1** between 2.4 × 10^–2^ and 1.2 × 10^–2^ M and concentrations of water between 12.2 and 4.9 M, starting from an initial concentration of nitrile of 0.24 M.

**Figure 4:**
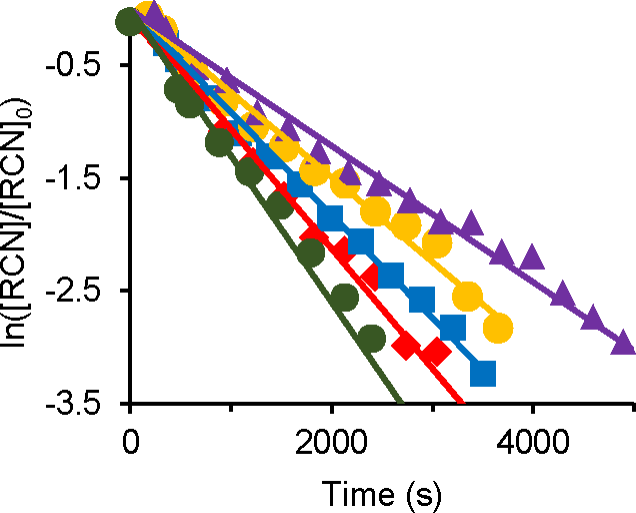
Plot of eq 3 for hydration of 2-methylpropanenitrile (0.24 M) with different concentrations of **1** in THF-*d*_8_ at 373 K. [H_2_O] = 12.2 M; [**1**] = 1.2 × 10^–2^ M (purple ▲); 1.5 × 10^–2^ M (yellow ●); 1.7 × 10^–2^ M (blue ■); 1.9 × 10^–2^ M (red ◆); 2.4 × 10^–2^ M (green ●).

The decrease of the nitrile concentration with a corresponding increase of the amide concentration is an exponential function of time under the selected conditions, in agreement with a pseudo-first-order process. The rate constant *k*^obs^ for each concentration of the catalyst precursor and water used and each temperature was calculated by graphing the expression shown in eq 3, as exemplified in Figure 4 for the reactions performed at 373 K, with a concentration of water of 12.2 M. The obtained values are collected in Table 2.
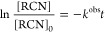
3

**Table 2:** Kinetic Data for Hydration of 2-Methylpropanenitrile (0.24 M) in THF-*d*_8_ Catalyzed by **1**

*T* (K)	[**1**]_0_ (×10^2^ M)	[H_2_O]_0_ (M)	*k*^obs^ (×10^4^ s^–1^)	*k*_1_^obs^ (×10^2^ M^–1^ s^–1^)	*k* (×10^3^ M^–2^ s^–1^)
373	2.4	12.2	13.0 ± 2.0	5.3 ± 0.5	4.4 ± 0.4
373	1.9	12.2	10.6 ± 0.7	5.5 ± 0.6	4.5 ± 0.5
373	1.7	12.2	9.1 ± 0.4	5.4 ± 0.5	4.4 ± 0.4
373	1.5	12.2	7.3 ± 0.7	5.1 ± 0.5	4.2 ± 0.4
373	1.2	12.2	6.2 ± 0.4	5.1 ± 0.5	4.2 ± 0.4
373	2.4	9.7	10.6 ± 0.4	4.4 ± 0.4	4.5 ± 0.5
373	2.4	8.5	8.6 ± 0.5	3.5 ± 0.4	4.1 ± 0.4
373	2.4	7.3	7.6 ± 0.4	3.1 ± 0.3	4.3 ± 0.4
373	2.4	6.1	6.9 ± 0.3	2.8 ± 0.3	4.6 ± 0.5
373	2.4	4.9	5.0 ± 0.3	2.1 ± 0.2	4.2 ± 0.4
363	2.4	12.2	5.3 ± 0.4	2.2 ± 0.2	1.8 ± 0.2
358	2.4	12.2	4.0 ± 0.3	1.7 ± 0.2	1.4 ± 0.1
353	2.4	12.2	2.8 ± 0.2	1.1 ± 0.1	0.9 ± 0.1
348	2.4	12.2	1.9 ± 0.1	0.8 ± 0.1	0.6 ± 0.1

The rate constant *k*^obs^ is a function of the concentrations of the catalyst precursor and water, according to eqs 4 and 5:
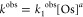
4
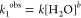
5

**Figure 5:**
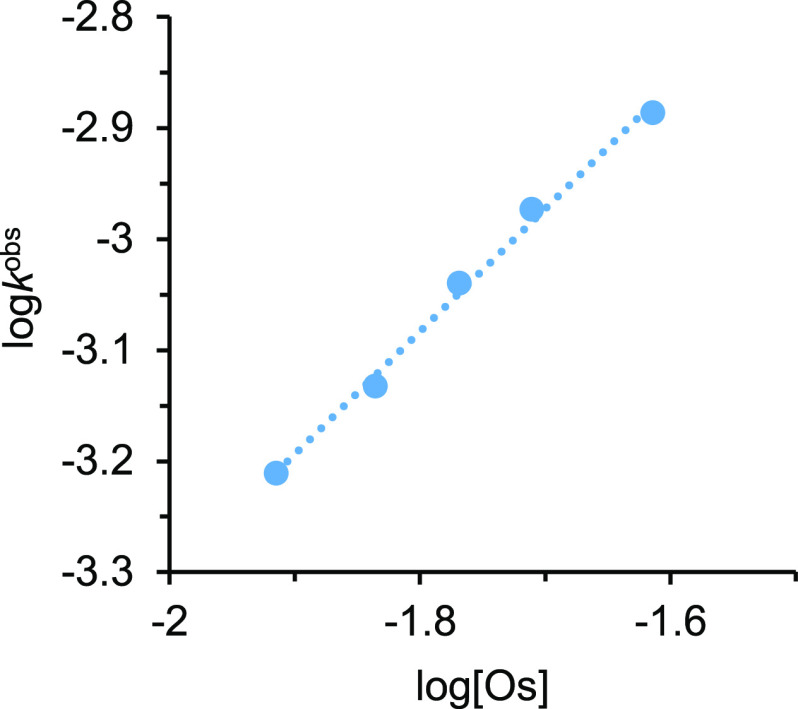
Plot of log *k*^obs^ versus log [Os] for hydration of 2-methylpropanenitrile (0.24 M) catalyzed by **1** in THF-*d*_8_ at 373 K.

**Figure 6:**
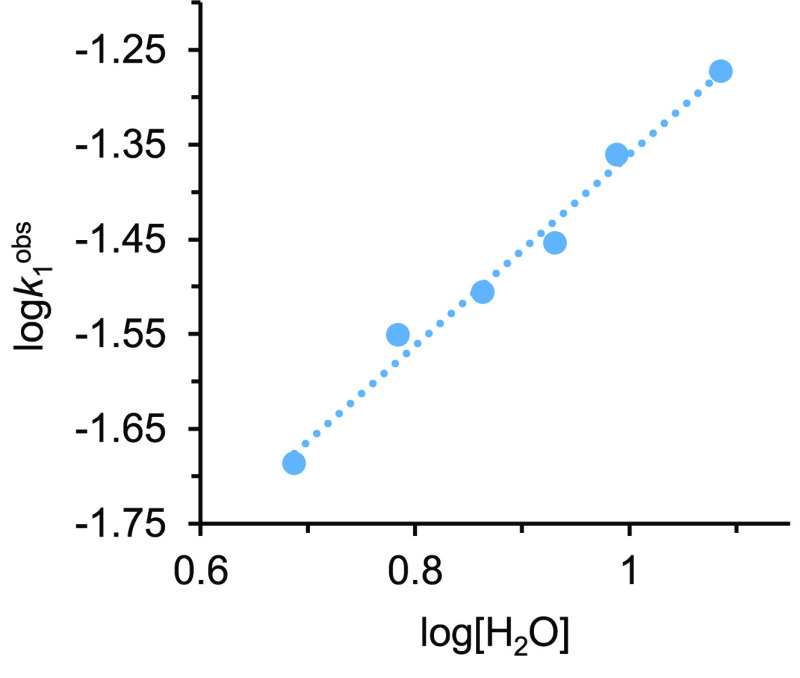
Plot log *k*_*1*_^obs^ versus log [H_2_O] for hydration of 2-methylpropanenitrile (0.24 M) catalyzed by **1** (2.4 × 10^–2^ M) in THF-*d*_8_ at 373 K.

A plot of log *k*^obs^ versus log [Os], for a water concentration of 12.2 M, yields a straight line of slope 1.1 (Figure 5), revealing that the hydration is first-order also in the catalyst concentration and therefore the values of *k*_1_^obs^ given in Table 2 were obtained from eq 4 for *a* = 1. Similarly, the plot of log *k*_1_^obs^ versus log [H_2_O], for a concentration of the catalyst precursor of 2.4 × 10^–2^ M, affords a straight line of slope 1.0 (Figure 6), proving that the reaction is also first-order in the water concentration, i.e., *b* = 1 in eq 5. Thus, the rate law is described by eq 6, where *k*[H_2_O] = *k*_1_^obs^ and *k*_1_^obs^[Os] = *k*^obs^.
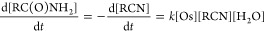
6

**Figure 7:**
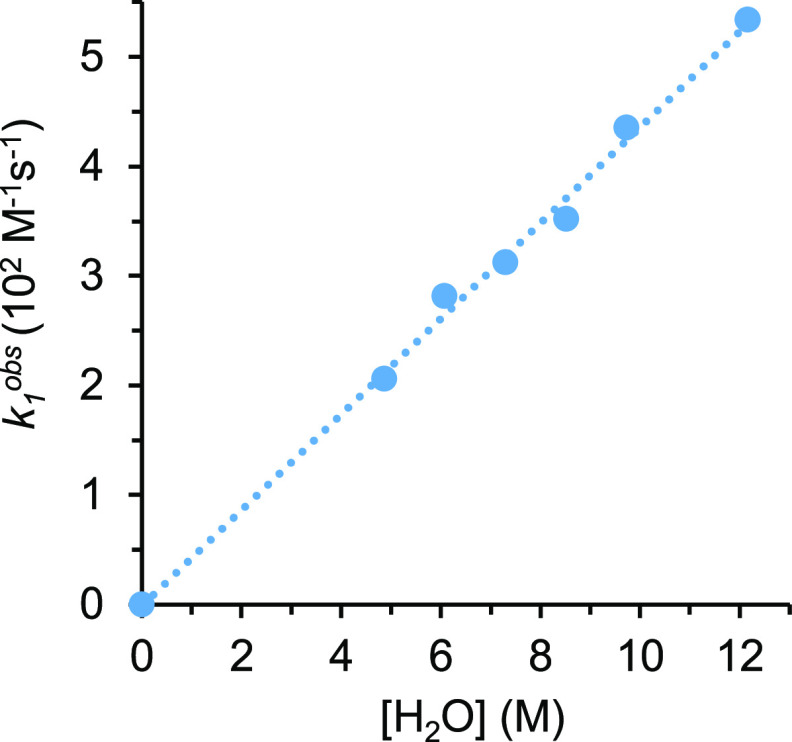
Plot of *k*_1_^obs^ versus [H_2_O] for hydration of 2-methylpropanenitrile (0.24 M) with water catalyzed by **1** (2.4 × 10^–2^ M) in THF-*d*_8_ at 373 K.

The plot of *k*_1_^obs^ versus [H_2_O] (Figure 7) provides a value of (4.4 ± 0.4) × 10^–3^ M^–2^ s^–1^ for *k* at 373 K.

The rate law described in eq 6 excludes the reaction of κ^2^-amidate complexes with water as the rate-determining step of nitrile hydration. In this context, it should be noted that, because the concentration of metal introduced in the catalysis is approximately equal to the concentration of the κ^2^-amidate complex generated during hydration, such a rate-determining step should yield a second-order reaction, independent of the nitrile concentration.

**Scheme 7:**
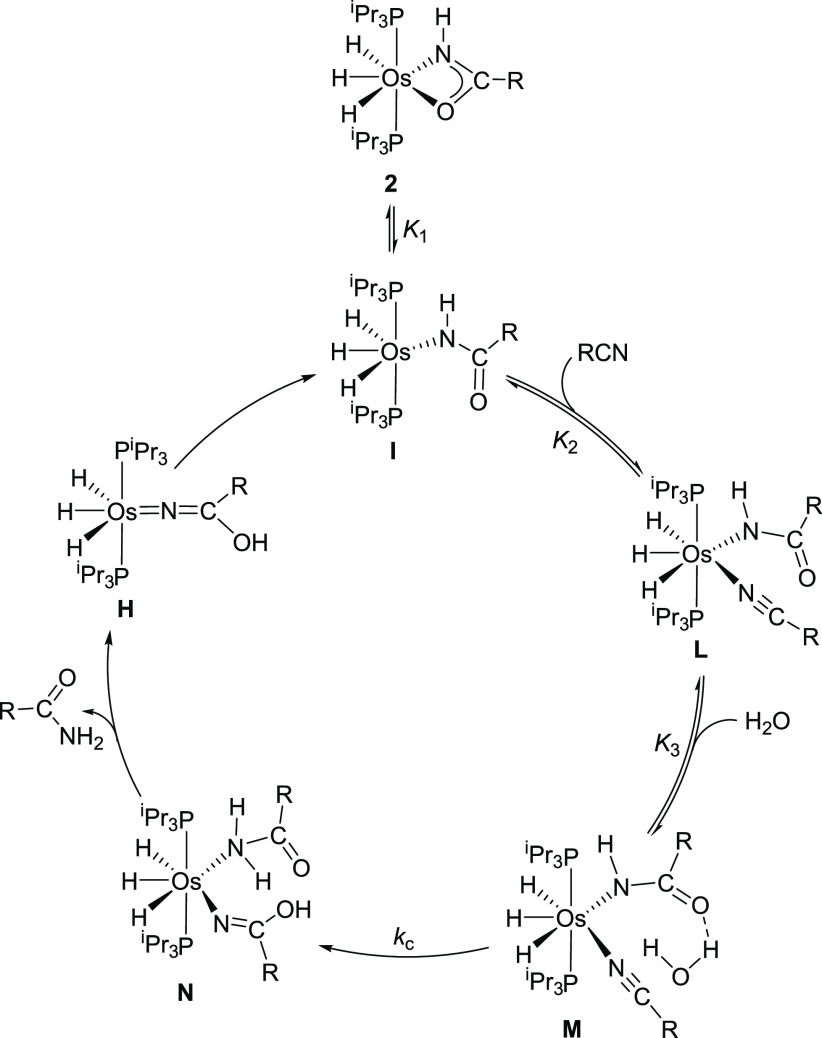
Catalytic Cycle for Nitrile Hydration

**Figure 8:**
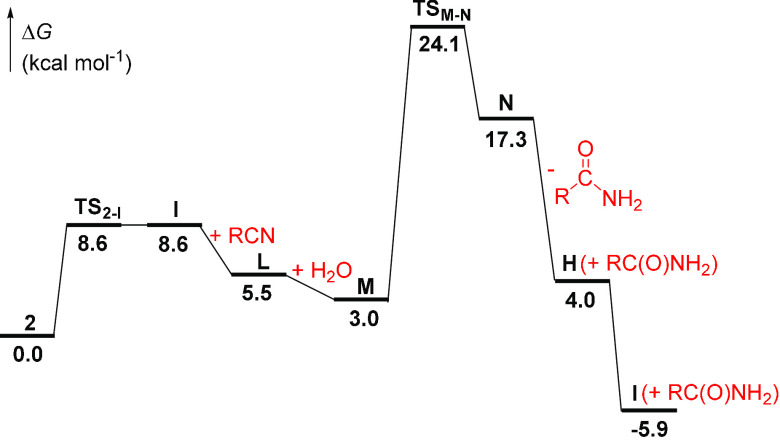
Computed energy profile for the catalytic cycle shown in Scheme 7 (R = Et).

The obtained rate law indicates that both nitrile and water are involved in the rate-determining step of hydration. To gain information about it, we extended the previous DFT calculations to the catalytic cycle (Scheme 7). Figure 8 shows the calculated profile for propionitrile as the model nitrile.

**Figure 9:**
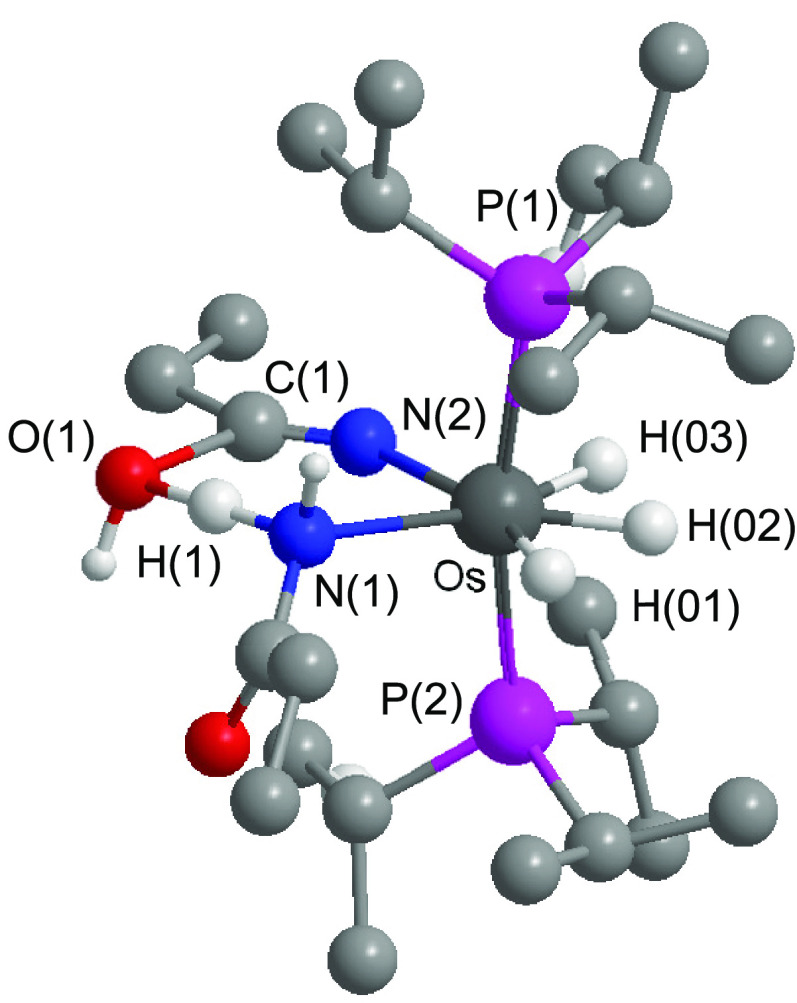
Transition state (TS_**M**–**N**_) between intermediates **M** and **N**. H atoms of the ethyl group and triisopropilphosphine ligands have been omitted for clarity. Selected bond distances (Å) and angles (deg): Os–N(1) = 2.363, Os–N(2) = 2.118, H(1)–N(1) = 1.231, H(1)–O(1) = 1.272, C(1)–O(1) = 1.952, C(1)–N(2) = 1.186; N(1)–Os–N(2) = 81.6.

The κ^2^-*N*,*O* to κ^1^-*N* transformation of the coordination mode of the amidate ligand of **2** affords the necessary coordination vacancy for entry of the nitrile molecule. Coordination of the nitrile to the κ^1^-*N*-amidate complex **I** leads to the seven-coordinate intermediate **L**, which is the key species of the catalysis. It undergoes the attack of an external water molecule in the rate-determining step, as expected according to eq 6. The free carbonyl group of the κ^1^-*N*-amidate ligand fixes the water molecule in the vicinity of the C(sp) atom of the nitrile. Once placed, the water molecule of adduct **M** attacks the C atom of the nitrile and the N atom of the amidate in a concerted manner. The attack takes place through a six-membered cyclic transition state, **TS_M-N_**, which involves C_nitrile_···O–H···N_amidate_ interactions (Figure 9). This transition state lies 24.1 kcal mol^–1^ above the κ^2^-amidate complex and ends up in the κ^1^-*N*-iminolate **N**, which resembles **K** bearing a κ^1^-*N*-amide instead of a water ligand. The amide dissociation from **N** affords the hydroxoazavinylidene intermediate **H**, which tautomerizes into the κ^1^-*N*-amidate complex **I**, closing the cycle.

The rate of formation of the amide is described by eq 7 according to the profile shown in Figure 7 and the rate-determining step approximation.
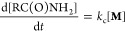
7The concentration of the intermediate **M** can be determined as follows:
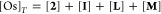
8Because [**L**] = [**M**]/*K*_3_[H_2_O] and [**I**] = [**M**]/*K*_2_*K*_3_[RCN][H_2_O], we have [**2**] = [**M**]/*K*_1_*K*_2_*K*_3_[RCN][H_2_O] and finally
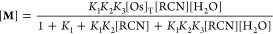
9Amidate complexes **2** are the only spectroscopically observed species during the course of hydration. As a consequence, we can assume that *K*_1_ + *K*_1_*K*_2_[RCN] + *K*_1_*K*_2_*K*_3_[RCN][H_2_O] ≪ 1, and therefore [**M**] can be described as follows:
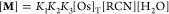
10Combining eqs 7 and 10, we obtain eq 11, where [Os]_T_ is the concentration of the catalyst precursor complex **1**.
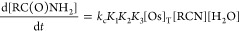
11Inspection of eq 11 shows that the rate of hydration is proportional to the concentrations of the catalyst precursor, nitrile, and water, in good agreement with the rate law obtained experimentally (see eq 6), where *k* = *k*_c_*K*_1_*K*_2_*K*_3_.

## Concluding Remarks

This study has discovered that the d^2^ hexahydride **1**, which is easily prepared from OsCl_3_·*x*H_2_O in high yield and bears a usual commercially available ligand, efficiently catalyzes the hydration of alkyl nitriles to amides. Furthermore, it displays a good tolerance to functional groups, including methoxide, benzoyl, functionalized aryl, and pyridyl groups, while also being active with substrates of a branched chain as the challenging trisubstituted pivalonitrile. The main metal species under the catalytic conditions are the trihydride osmium(IV) amidate derivatives OsH_3_{κ^2^-*N*,*O*-[HNC(O)R]}(P^i^Pr_3_)_2_, which are formed in a stoichiometric process involving three main steps: heterolytic O–H bond activation of water, nitrile coordination, and nucleophilic attack of a hydroxo group at the C(sp) atom of the coordinated nitrile.

Evidence obtained by combining isolation of the main metal species under the catalytic conditions, kinetic analysis of the hydration, and DFT calculations strongly supports an alternative mechanism to those previously reported. Each reaction has its own catalyst. The trihydride osmium(IV) amidate complexes OsH_3_{κ^2^-*N*,*O*-[HNC(O)R]}(P^i^Pr_3_)_2_ release the carbonyl group of the chelate to afford κ^1^-*N*-amidate derivatives, which are the true catalysts of hydration, one diffeent for each nitrile. These catalysts coordinate the nitrile to give the key intermediates of the catalysis, which undergo the attack of an external water molecule in the rate-determining step. The water molecule attacks the C atom of the nitrile and the N atom of the amidate in a concerted manner, through a six-membered cyclic transition state, which involves C_nitrile_···O–H···N_amidate_ interactions. The attack liberates the amide and regenerates a new κ^1^-*N*-amidate to continue the hydration. The group κ^1^-*N*-amidate is not only an intermediate in the formation pathway of the amide but also a noninnocent ligand, which cooperates in the external attack of the water molecule. Its free carbonyl group fixes the water molecule in the vicinity of the C(sp) atom of the nitrile, before the attack.

The electron density of the metal center of the precursor is responsible of the formation of these amidate catalysts; direct participation of the ligands of the precursor does not take place. Once the amidate complexes are formed, the steps involved in the catalytic cycle are also mainly governed by the amidate itself and the electron density of the metal center; the role of the ligands of the precursor is reduced to that typical in homogeneous catalysis: to modulate the electron density of the metal center and the space around it. According to this, it seems to be clear that the hydration of nitriles with catalyst precursors bearing only innocent ligands is possible.

## Experimental Section

General details including X-ray analysis, instrumental methods, and computational information are given in the Supporting Information. Chemical shifts are expressed in parts per million. Coupling constants are given in hertz (*N* = ^3^*J*_H–P_ + ^5^*J*_H–P′_ for ^1^H and ^1^*J*_C–P_ + ^3^*J*_C–P′_ for ^13^C).

### Preparation of OsH_3_{κ^2^-*N*,*O*-[HNC(O)CH(CH_3_)_2_]}(P^i^Pr_3_)_2_ (**2a**)

2-Methylpropanenitrile (35.9 μL, 0.4 mmol) and water (7.2 μL, 0.4 mmol) were placed in a Schlenk tube with a solution of **1** (100 mg, 0.19 mmol) in THF (2 mL). The Schlenk tube was heated at 100 °C for 3 h. The solvent was eliminated in vacuo to give a yellow oil. The oil was washed with several portions of cold pentane (3 × 2 mL at −78 °C) and dried in vacuo. Yield: 60 mg (50%). HR-MS (electrospray). Calcd for C_22_H_52_NOOsP_2_ ([M – H]^+^): *m*/*z* 600.3101. Found: *m*/*z* 600.3133. ^1^H NMR (300.13 MHz, C_7_D_8_, 298 K): δ 5.30 (br, 1H, NH), 1.99 (m, 7H, CHP^i^Pr_3_ + CH^i^Pr), 1.22 (dvt, ^3^*J*_H–H_= 6.7, *N* = 12.6, 36H, CH_3_P^i^Pr_3_), 0.98 (d, ^3^*J*_H–H_= 7.0, 6H, CH_3_^i^Pr), −13.26 (br, 3H, OsH_3_). ^1^H NMR (300.13 MHz, C_7_D_8_, 183 K): δ 5.30 (br, 1H, NH), 1.91 (br, 7H, CHP^i^Pr_3_ + CH^i^Pr), 1.23 (br, 36H, CH_3_P^i^Pr_3_), 1.00 (br, 6H, CH_3_^i^Pr), −10.28 (br, 1H, OsH), −13.80 (br, 1H, OsH), −15.14 (br, 1H, OsH). ^31^P{^1^H} NMR (121.50 MHz, C_7_D_8_, 298 K): δ 36.5. ^13^C{^1^H} APT NMR (75.48 MHz, CDCl_3_, 298 K): δ 181.2 (NCO), 37.6 (CH^i^Pr), 26.6 (vt, *N* = 23, CH^i^Pr), 20.3 (CH_3_P^i^Pr_3_), 18.5 (CH_3_^i^Pr).

### Preparation of OsH_3_{κ^2^-*N*,*O*-[HNC(O)C(CH_3_)_3_]}(P^i^Pr_3_)_2_ (**2b**)

Complex **1** (100 mg, 0.19 mmol) in THF (2 mL) was treated with pivalonitrile (44.2 μL, 0.4 mmol) and water (7.2 μL, 0.4 mmol) for 3 h at 130 °C. The solvent was eliminated under vacuum, obtaining a yellow oil. The addition of cold pentane (1 mL at −78 °C) caused the precipitation of a white solid. The solid was washed with further portions of cold pentane (3 × 2 mL) and dried in vacuo. Yield: 35 mg (30%). Colorless single crystals suitable for X-ray diffraction analysis were obtained from a saturated solution of **2b** in pentane at −30 °C. HR-MS (electrospray). Calcd for C_23_H_54_NOOsP_2_ ([M – H]^+^): *m*/*z* 614.3291. Found: *m*/*z* 614.3302. Anal. Calcd for C_23_H_55_NOOsP_2_: C, 45.00; H, 9.03; N, 2.28. Found: C, 44.78; H, 8.85; N, 2.46. IR (ATR, cm^–1^): ν(NH) 3432 (w), ν(Os–H) 2126 (s). ^1^H NMR (300.13 MHz, C_7_D_8_, 298 K): δ 5.42 (br, 1H, NH), 1.99 (m, 6H, CHP^i^Pr_3_), 1.20 (dvt, ^3^*J*_H–H_ = 5.6, *N* = 12.5, 36H, CH_3_P^i^Pr_3_), 1.02 (s, 9H, CH_3_^t^Bu), −13.36 (br, 3H, OsH_3_). ^1^H NMR (300.13 MHz, C_7_D_8_, 193 K): δ 5.45 (br, 1H, NH), 1.89 (br, 6H, CHP^i^Pr_3_), 1.26 (br, 36H, CH_3_P^i^Pr_3_), 1.06 (s, 9H, CH_3_^t^Bu), −10.33 (br, 1H, OsH), −13.77 (br, 1H, OsH), −14.94 (br, 1H, OsH). ^31^P{^1^H} NMR (121.50 MHz, C_7_D_8_, 298 K): δ 37.0. ^13^C{^1^H} APT NMR (75.48 MHz, CDCl_3_, 298 K): δ 182.5 (NCO), 39.9 (C_q_^t^Bu), 26.8 (CH_3_^t^Bu), 26.4 (vt, *N* = 23.1, CHP^i^Pr), 20.3 (CH_3_P^i^Pr_3_).

### Catalytic Hydration of Nitriles

All reactions were performed in NMR tubes under an argon atmosphere. Nitrile (0.14 mmol), deoxygenated water (125 μL, 7.0 mmol), and mesitylene (19.5 μL, 0.14 mmol), used as an internal standard, were added to a solution of **1** (3.6 mg, 0.007 mmol, 5 mol %) in THF-*d*_8_ (450 μL). The mixture was heated at 100 °C, and the reaction was monitored by ^1^H NMR. The yields were determined by comparing the integration areas of the characteristic signals of the amides with those of the mesitylene. After the time indicated on Scheme 4, the solvent and remaining water were removed under vacuum, yielding a silvery oil or a white solid. The addition of pentane (1 mL) induced the precipitation of a white solid, which was washed with further portions of pentane (3 × 1 mL) and dried under vacuo. The amides were characterized by ^1^H and ^13^C{^1^H} NMR and IR spectroscopy.

### Kinetic Experiments

All kinetic experiments were performed in THF-*d*_8_ solutions contained in NMR tubes under an argon atmosphere. The NMR tubes were charged with 2-methylpropanenitrile (0.14 mmol, 0.24 M), water (50.4−125 μL, 2.8–7.0 mmol, 4.9–12.2 M), complex **1** (7.0 × 10^–3^–14.0 × 10^–3^ mmol, 1.2 × 10^–2^–2.4 × 10^–2^ M), and mesitylene (0.14 mmol, 0.24 M; internal standard), and the final volume was brought to 575 μL using THF-*d*_8_. Then ^1^H NMR spectra were recorded every 5 min for 1 h or until the conversion was over 90%.

## Supporting Information Available

General information, structural analysis, computational details, analytical data of the isolated amides, NMR spectra, energies of computed structures, and comparative tables of metal-catalyzed nitrile hydration (PDF)
Cartesian coordinates of calculated structures (XYZ)

## Supplementary Material

ic1c00380_si_001.pdf

ic1c00380_si_002.xyz

## Accession Codes

CCDC 2057271 contains the supplementary crystallographic data for this paper. These data can be obtained free of charge via www.ccdc.cam.ac.uk/data_request/cif, or by emailing data_request@ccdc.cam.ac.uk, or by contacting The Cambridge Crystallographic Data Centre, 12 Union Road, Cambridge CB2 1EZ, UK; fax: +44 1223 336033.

## References

[ref1] Greenberg A., Breneman C. M., Liebman J. F., Eds. The Amide Linkage: Structural Significance in Chemistry, Biochemistry, and Material Science; John Wiley and Sons: New York, 2000.

[ref2] aKovács E.; Rózsa B.; Csomos A.; Csizmadia I. G.; Mucsi Z. Amide Activation in Ground and Excited States. Molecules 2018, 23, 2859 10.3390/molecules23112859. PMC6278462 30400217

[ref3] De Figueiredo R. M.; Suppo J.-S.; Campagne J.-M. Nonclassical Routes for Amide Bond Formation. Chem. Rev. 2016, 116, 12029–12122. 10.1021/acs.chemrev.6b00237. 27673596

[ref4] Kettler P. B. Platinum Group Metals in Catalysis: Fabrication of Catalysts and Catalyst Precursors. Org. Process Res. Dev. 2003, 7, 342–354. 10.1021/op034017o.

[ref5] aKukushkin V. Y.; Pombeiro A. J. L. Additions to Metal-Activated Organonitriles. Chem. Rev. 2002, 102, 1771–1802. 10.1021/cr0103266. 11996549

[ref6] aKaminskaia N. V.; Kostic N. M. Nitrile hydration catalysed by palladium(II) complexes. J. Chem. Soc., Dalton Trans. 1996, 3677–3686. 10.1039/dt9960003677.

[ref7] aGulyás H.; Rivilla I.; Curreli S.; Freixa Z.; van Leeuwen P. W. N. M. Highly active, chemo- and enantioselective Pt-SPO catalytic systems for the synthesis of aromatic carboxamides. Catal. Sci. Technol. 2015, 5, 3822–3828. 10.1039/C5CY00627A.

[ref8] aOshiki T.; Yamashita H.; Sawada K.; Utsunomiya M.; Takahashi K.; Takai K. Dramatic Rate Acceleration by a Diphenyl-2-Pyridylphosphine Ligand in the Hydration of Nitriles Catalyzed by Ru(acac)_2_ Complexes. Organometallics 2005, 24, 6287–6290. 10.1021/om050792b.

[ref9] aLeung C. W.; Zheng W.; Zhou Z.; Lin Z.; Lau C. P. Mechanism of Catalytic Hydration of Nitriles with Hydrotris(pyrazolyl)borato (Tp) Ruthenium Complexes. Organometallics 2008, 27, 4957–4969. 10.1021/om800474w.

[ref10] Ma X.; He Y.; Lu M. Efficient Mo(VI)-Catalyzed Hydration of Nitrile with Acetaldoxime. Synth. Commun. 2014, 44, 474–480. 10.1080/00397911.2013.806668.

[ref11] aStepanenko I. N.; Cebrián-Losantos B.; Arion V. B.; Krokhin A. A.; Nazarov A. A.; Keppler B. K. The Complexes [OsCl_2_(azole)_2_(dmso)_2_] and [OsCl_2_(azole)(dmso)_3_]: Synthesis, Structure, Spectroscopic Properties and Catalytic Hydration of Chloronitriles. Eur. J. Inorg. Chem. 2007, 2, 400–411. 10.1002/ejic.200600859.

[ref12] aFung W. K.; Huang X.; Man M. L.; Ng S. M.; Hung M. Y.; Lin Z.; Lau C. P. Dihydrogen-Bond-Promoted Catalysis: Catalytic Hydration of Nitriles with the Indenylruthenium Hydride Complex (η^5^-C_9_H_7_)Ru(dppm)H (dppm = Bis(diphenylphosphino)methane). J. Am. Chem. Soc. 2003, 125, 11539–11544. 10.1021/ja034050q. 13129356

[ref13] aJensen C. M.; Trogler W. C. Kinetics and Mechanism of Nitrile Hydration Catalyzed by Unhindered Hydridobis(phosphine)platinum(II) Complexes. Regioselective Hydration of Acrylonitrile. J. Am. Chem. Soc. 1986, 108, 723–729. 10.1021/ja00264a025.

[ref14] Swartz R. D.; Coggins M. K.; Kaminsky W.; Kovacs J. A. Nitrile Hydration by Thiolate- and Alkoxide-Ligated Co-NHase Analogues. Isolation of Co(III)-Amidate and Co(III)-Iminol Intermediates. J. Am. Chem. Soc. 2011, 133, 3954–3963. 10.1021/ja108749f. 21351789 PMC3151161

[ref15] aTílvez E.; Menéndez M. I.; López R. On the Mechanism of the [Cp_2_Mo(OH)(OH_2_)]^+^-Catalyzed Nitrile Hydration to Amides: A Theoretical Study. Organometallics 2012, 31, 1618–1626. 10.1021/om200541z.

[ref16] aGhaffar T.; Parkins A. W. A New Homogeneous Platinum Containing Catalyst for the Hydrolysis of Nitriles. Tetrahedron Lett. 1995, 36, 8657–8660. 10.1016/0040-4039(95)01785-G.

[ref17] Xing X.; Xu C.; Chen B.; Li C.; Virgil S. C.; Grubbs R. H. Highly Active Platinum Catalysts for Nitrile and Cyanohydrin Hydration: Catalyst Design and Ligand Screening via High-Throughput Techniques. J. Am. Chem. Soc. 2018, 140, 17782–17789. 10.1021/jacs.8b11667. 30482014

[ref18] Fan X.-N.; Deng W.; Liu Z.-J.; Yao Z.-J. Half-Sandwich Iridium Complexes for the One-Pot Synthesis of Amides: Preparation, Structure, and Diverse Catalytic Activity. Inorg. Chem. 2020, 59, 16582–16590. 10.1021/acs.inorgchem.0c02497. 33113329

[ref19] aKolb H. C.; VanNieuwenhze M. S.; Sharpless K. B. Catalytic Asymmetric Dihydroxylation. Chem. Rev. 1994, 94, 2483–2547. 10.1021/cr00032a009.

[ref20] aEsteruelas M. A.; Oro L. A.; Valero C. Hydrogenation of Benzylideneacetone Catalyzed by OsHCl(CO)(PR_3_)_2_ (PR_3_ = P-*i*-Pr_3_, PMe-*t*-Bu_2_): New Roles of Dihydrogen Complexes in Homogeneous Catalytic Hydrogenation. Organometallics 1992, 11, 3362–3369. 10.1021/om00046a040.

[ref21] aEsteruelas M. A.; Herrero J.; López A. M.; Oliván M. Alkyne-Coupling Reactions Catalyzed by OsHCl(CO)(P^i^Pr_3_)_2_ in the Presence of Diethylamine. Organometallics 2001, 20, 3202–3205. 10.1021/om010178+.

[ref22] aBarrio P.; Esteruelas M. A.; Oñate E. Reactions of a Hexahydride-Osmium Complex with Aldehydes: Double C-H*_α_* Activation-Decarbonylation and Single C-H*_α_* Activation-Hydroxylation Tandem Processes and Catalytic Tishchenko Reactions. Organometallics 2004, 23, 1340–1348. 10.1021/om034389l.

[ref23] aBaratta W.; Bossi G.; Putignano E.; Rigo P. Pincer and Diamine Ru and Os Diphosphane Complexes as Efficient Catalysts for the Dehydrogenation of Alcohols to Ketones. Chem. - Eur. J. 2011, 17, 3474–3481. 10.1002/chem.201003022. 21341330

[ref24] aEsteruelas M. A.; Fernández I.; López A. M.; Mora M.; Oñate E. Osmium-Promoted Dehydrogenation of Amine-Boranes and B-H Bond Activation of the Resulting Amino-Boranes. Organometallics 2014, 33, 1104–1107. 10.1021/om500027p.

[ref25] Buil M. L.; Cadierno V.; Esteruelas M. A.; Gimeno J.; Herrero J.; Izquierdo S.; Oñate E. Selective Hydration of Nitriles to Amides Promoted by an Os-NHC Catalyst: Formation and X-Ray Characterization of κ^2^-Amidate Intermediates. Organometallics 2012, 31, 6861–6867. 10.1021/om3006799.

[ref26] González-Fernández R.; Crochet P.; Cadierno V.; Menéndez M. I.; López R. Phosphinous Acid-Assisted Hydration of Nitriles: Understanding the Controversial Reactivity of Osmium and Ruthenium Catalysts. Chem. - Eur. J. 2017, 23, 15210–15221. 10.1002/chem.201703481. 28816406

[ref27] Esteruelas M. A.; López A. M.; Oliván M. Polyhydrides of Platinum Group Metals: Nonclassical Interactions and σ-Bond Activation Reactions. Chem. Rev. 2016, 116, 8770–8847. 10.1021/acs.chemrev.6b00080. 27268136

[ref28] aAlabau R. G.; Eguillor B.; Esler J.; Esteruelas M. A.; Oliván M.; Oñate E.; Tsai J.-Y.; Xia C. CCC-Pincer-NHC Osmium Complexes: New Types of Blue-Green Emissive Neutral Compounds for Organic Light-Emitting Devices (OLEDs). Organometallics 2014, 33, 5582–5596. 10.1021/om500905t.

[ref29] Aracama M.; Esteruelas M. A.; Lahoz F. J.; López J. A.; Meyer U.; Oro L. A.; Werner H. Synthesis, Reactivity, Molecular Structure, and Catalytic Activity of the Novel Dichlorodihydridoosmium(IV) Complexes OsH_2_Cl_2_(PR_3_)_2_ (PR_3_ = P-*i*-Pr_3_, PMe-*t*-Bu_2_). Inorg. Chem. 1991, 30, 288–293. 10.1021/ic00002a025.

[ref30] Abdur-Rashid K.; Gusev D. G.; Lough A. J.; Morris R. H. Synthesis and Characterization of RuH_2_(H_2_)_2_(P^i^Pr_3_)_2_ and Related Chemistry. Evidence for a Bis(dihydrogen) Structure. Organometallics 2000, 19, 1652–1660. 10.1021/om990669i.

[ref31] Babón J. C.; Esteruelas M. A.; Fernández I.; López A. M.; Oñate E. Reduction of Benzonitriles via Osmium-Azavinylidene Intermediates Bearing Nucleophilic and Electrophilic Centers. Inorg. Chem. 2019, 58, 8673–8684. 10.1021/acs.inorgchem.9b01013. 31247858

[ref32] Babón J. C.; Esteruelas M. A.; Fernández I.; López A. M.; Oñate E. Redox-Assisted Osmium-Promoted C-C Bond Activation of Alkylnitriles. Organometallics 2018, 37, 2014–2017. 10.1021/acs.organomet.8b00326.

[ref33] Babón J. C.; Esteruelas M. A.; López A. M.; Oñate E. Osmium-Promoted Transformation of Alkyl Nitriles to Secondary Aliphatic Amines: Scope and Mechanism. Organometallics 2020, 39, 2177–2188. 10.1021/acs.organomet.0c00236.

[ref34] Babón J. C.; Esteruelas M. A.; Fernández I.; López A. M.; Oñate E. Dihydroboration of Alkyl Nitriles Catalyzed by an Osmium-Polyhydride: Scope, Kinetics, and Mechanism. Organometallics 2020, 39, 3864–3872. 10.1021/acs.organomet.0c00582.

[ref35] Eguillor B.; Esteruelas M. A.; García-Raboso J.; Oliván M.; Oñate E. Stoichiometric and Catalytic Deuteration of Pyridine and Methylpyridines by H/D Exchange with Benzene-*d*_6_ Promoted by an Unsaturated Osmium Tetrahydride Species. Organometallics 2009, 28, 3700–3709. 10.1021/om900335b.

[ref36] aOzerov O. V. Oxidative addition of water to transition metal complexes. Chem. Soc. Rev. 2009, 38, 83–88. 10.1039/B802420K. 19088967

[ref37] Antiñolo A.; Esteruelas M. A.; García-Yebra C.; Martín J.; Oñate E.; Ramos A. Reactions of an Osmium(IV)-Hydroxo Complex with Amino-Boranes: Formation of Boroxide Derivatives. Organometallics 2019, 38, 310–318. 10.1021/acs.organomet.8b00727.

[ref38] Esteruelas M. A.; Oliván M.; Oñate E. Sigma-Bond Activation Reactions Induced by Unsaturated Os(IV)-Hydride Complexes. Adv. Organomet. Chem. 2020, 74, 53–104. 10.1016/bs.adomc.2020.04.002.

