# Supplementary material for: Hydration of Aliphatic Nitriles Catalyzed by an Osmium Polyhydride: Evidence for an Alternative Mechanism
Source: Inorg Chem. 2021 Apr 27;60(10):7284–96. doi: 10.1021/acs.inorgchem.1c00380 (PMC8892838; doi:10.1021/acs.inorgchem.1c00380)
Supplement: Supplementary file 1 — ic1c00380_si_001.pdf [file ic1c00380_si_001.pdf]

Supporting Information

Hydration of Aliphatic Nitriles Catalyzed by an Osmium Polyhydride: Evidence for an Alternative Mechanism

Juan C. Babón, Miguel A. Esteruelas, Ana M. López, and Enrique Oñate*

Departamento de Química Inorgánica, Instituto de Síntesis Química y Catálisis Homogénea (ISQCH), Centro de Innovación en Química Avanzada (ORFEO-CINQA), Universidad de Zaragoza-CSIC, 50009 Zaragoza, Spain

* Corresponding author's e-mail address: maester@unizar.es

Contents:

Experimental Section: General Information…… S2

Structural analysis of complex **2b**…… S2

Computational details…… S3

Analytical data of the isolated amides…… S3

References…… S7

^{1}H NMR spectra of the reaction crude of the catalytic nitrile hydrations,

^{1}H, ^{13}C{^{1}H} APT NMR, and IR spectra of the amides…… S10

^{1}H, ^{13}C{^{1}H} APT NMR, and IR spectra of complexes **2a** and **2b**…… S52

Energies of computed structures…… S56

Comparative tables of metal-catalyzed nitrile hydration…… S64

Experimental Section: General information. All manipulations were performed with rigorous exclusion of air at an argon/vacuum manifold using standard Schlenk-tube or glovebox techniques. Solvents were dried by the usual procedures and distilled under argon prior to use or obtained oxygen- and water-free from an MBraun solvent purification apparatus. THF-d_8 was stored over sodium in the glovebox. Complex $OsH_6(P^iPr_3)_2$ (**1**) was prepared according to the published method.[1] Nitriles were purchased from commercial sources and distilled in a Kugelrohr distillation oven. NMR spectra were recorded on a Bruker ARX 300, Bruker Avance 300 MHz, or a Bruker Avance 400 MHz instruments. Chemical shifts (expressed in parts per million) are referenced to residual solvent peaks (^1H, $^{13}C\{^1H\}$) and external H_3PO_4 ($^{31}P\{^1H\}$) or $CFCl_3$ (^{19}F). Coupling constants *J* are given in Hertz. High-resolution (HRMS) electrospray mass spectra were acquired using a MicroTOF-Q hybrid quadrupole time-of-flight spectrometer (Bruker Daltonics, Bremen, Germany). C, H, and N analyses were carried out in a Perkin-Elmer 2400-B Series II CHNS-Analyzer. Attenuated total reflection infrared spectra (ATR-IR) of solid samples were run on a Perkin-Elmer Spectrum 100 FT-IR spectrometer.

Structural analysis of complex 2b. X-ray data were collected on a Bruker APEX DUO diffractometer equipped with a normal or fine focus, and 2.4 kW sealed tube source (Mo radiation, $\lambda = 0.71073$ Å). Data were collected over the complete sphere covering 0.3° in ω. Data were corrected for absorption by using a multiscan method applied with the SADABS program.[2] The structures were solved by Patterson or direct methods and refined by full-matrix least squares on F^2 with SHELXL2016,[3] including isotropic and subsequently anisotropic displacement parameters. The hydrogen atoms were observed in the last Fourier Maps or calculated and refined freely or using a restricted riding model. The hydride ligands were observed in the difference Fourier maps and refined with a restrained distance to osmium atoms.

Crystal data for **2b**: $C_{23}H_{55}NOOsP_2$, M_W 613.82, yellow, irregular block (0.135 x 0.148 x 0.313 mm^3), monoclinic, space group P21/c, *a*: 9.3498(7) Å, *b*: 29.037(2) Å, *c*: 11.1353(8) Å, *β*: 112.5400(10)°, $V = 2792.2(4)$ Å^3, $Z = 4$, $Z' = 1$, D_{calc}: 1.460 g cm^{-3}, F(000): 1256, T = 100(2) K, μ 4.695 mm^{-1}. 33123 measured reflections (2θ: 3-57°, ω scans 0.3°), 7506 unique ($R_{int} = 0.1137$); min./max. transm. Factors 0.637/0.862. Final agreement factors were $R^1 = 0.0221$(6710 observed reflections, $I > 2\sigma(I)$) and $wR^2 =$

0.0513; data/restraints/parameters 7506/9/ 277; GoF = 1.076. Largest peak and hole 1.670 (close to osmiun atoms) and –0.858 e/ Å^3.

Computational details: All calculations in the mechanistic studies were performed at the DFT level using the B3LYP functional[4] supplemented with the Grimme's dispersion correction D3[5] including an ultrafine integration grid, as implemented in Gaussian09.[6] Os atom was described by means of an effective core potential SDD for the inner electron[7] and its associated double-ζ basis set for the outer ones, complemented with a set of f-polarization functions.[8] The 6-31G** basis set was used for the H, C, N, O, and P atoms.[9] All geometries were fully optimized in THF (ε = 7.58) solvent using the continuum SMD model.[10] Transition states were identified by having one imaginary frequency in the Hessian matrix. It was confirmed that transition states connect with the corresponding intermediates by means of application of an eigenvector corresponding to the imaginary frequency and subsequent optimization of the resulting structures. Gibbs energies were computed at 298.15 K and 1 atmosphere. All values collected in schemes and figures correspond to Gibbs energies in toluene in kcal mol^{-1}.

The Cartesian coordinates for the computed structures can be found in the supplemental file xyz. The file may be opened as a text file to read the coordinates, or opened directly by a molecular modeling program such as Mercury (version 3.3 or later, http://www.ccdc.cam.ac.uk/pages/Home.aspx) for visualization and analysis.

Analytical data of the isolated amides

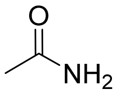

Acetamide:[11] ^{1}H NMR (300.13 MHz, $CDCl_3$, 298 K): δ 5.49 (br, 2H, NH_2), 2.04 (s, 3H, CH_3). $^{13}C\{^1H\}$ APT NMR (75.48 MHz, $CDCl_3$, 298 K): δ 172.4 (CO), 22.6 (CH_3). IR (ATR, cm^{-1}): ν(N–H) 3313 (s), 3185 (s); ν(CO) 1924 (s).

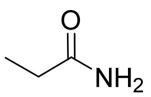

Propionamide:[12] ^{1}H NMR (300.13 MHz, $CDCl_3$, 298 K): δ 5.44 (br, 2H, NH_2), 2.28 (q, $^3J_{H–H}$ = 7.6, 2H, CH_2), 1.19 (t, $^3J_{H–H}$ = 7.6, 3H, CH_3). $^{13}C\{^1H\}$ APT NMR (75.48 MHz, $CDCl_3$, 298 K): δ 176.0 (CO), 28.9 (CH_2CO), 9.6 (CH_3). IR (ATR, cm^{-1}): ν(N–H) 3320 (s), 3169 (s); ν(CO) 1893 (s).

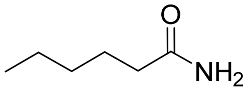

Hexanamide:[13] ^{1}H NMR (300.13 MHz, $CDCl_3$, 298 K): δ 5.57 (br, 2H, NH_2), 2.23 (t, $^3J_{H–H}$ = 7.6, 2H, CH_2CO), 1.65 (m, 2H, CH_2), 1.33 (m, 4H, CH_2CH_2), 0.91(m, 3H, CH_3).$^{13}C\{^1H\}$ APT NMR (75.48 MHz, $CDCl_3$, 298

K): δ 175.7 (CO), 35.9, 31.4, 25.2, 22.4 (all CH_2), 13.9 (CH_3). IR (ATR, cm^{-1}): ν(N–H) 3356 (s), 3178 (s); ν(CO) 1594 (s)

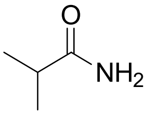

2-Methylpropionamide:[14] ^1H NMR (400.16 MHz, $CDCl_3$, 298 K): δ 5.27 (br, 2H, NH_2), 2.28 (hept, $^3J_{H\text{–}H}$ = 6.9, 1H, CH), 1.03 (d, $^3J_{H\text{–}H}$ = 6.9, 6H, CH_3). $^{13}C\{^1H\}$ APT NMR (75.48 MHz, $CDCl_3$, 298 K): δ 179.5 (CO), 35.0 (*C*HCO), 19.6 (CH_3). IR (ATR, cm^{-1}): ν(N–H) 3346 (s), 3169 (s); ν(CO) 1634 (s).

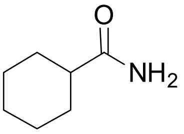

Ciclohexanecarboxamide:[15] ^1H NMR (400.16 MHz, $CDCl_3$, 298 K): δ 5.48 (br, 2H, NH_2), 2.16 (tt, $^3J_{H\text{–}H}$ = 11.7, $^3J_{H\text{–}H}$ = 3.5 Hz, 1H, COCH), 1.92 (m, 2H, CH_2), 1.81 (m, 2H, CH_2), 1.70 (m, 1H, CH_2), 1.44 (m, 2H, CH_2), 1.29 (m, 3H, CH_2). $^{13}C\{^1H\}$ APT NMR (75.48 MHz, $CDCl_3$, 298 K): δ 178.7 (CO), 44.8 (*C*HCO), 29.7, 25.7, 25.7 (all CH_2). IR (ATR, cm^{-1}): ν(N–H) 3333 (s), 3156 (s); ν(CO) 1633 (s).

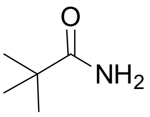

Pivalamide:[16] ^1H NMR (400.16 MHz, $CDCl_3$, 298 K): δ 5.48 (br, 2H, NH_2), 1.25 (s, 9H, CH_3). $^{13}C\{^1H\}$ APT NMR (75.48 MHz, $CDCl_3$, 298 K): δ 181.3 (CO), 38.6 (C_qCO), 27.6 (CH_3). IR (ATR, cm^{-1}): ν(N–H) 3392 (s), 3194 (s); ν(CO) 1621 (s).

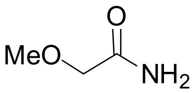

2-Methoxyacetamide:[17] ^1H NMR (300.13 MHz, $CDCl_3$, 298 K): δ 6.19 (br, 2H, NH_2), 3.92 (s, 2H, CH_2), 3.45 (s, 3H, OCH_3). $^{13}C\{^1H\}$ APT NMR (75.48 MHz, $CDCl_3$, 298 K): δ 172.5 (CO), 71.8 (CH_2), 59.2 (OCH_3). IR (ATR, cm^{-1}): ν(N–H) 3375 (s), 3185 (s); ν(CO) 1652 (s).

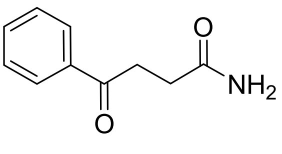

4-Oxo-4-phenylbutanamide:[18] ^1H NMR (300.13 MHz, $CDCl_3$, 298 K): δ 7.99 (m, 2H, CH Ph), 7.57 (m, 1H, CH Ph), 7.47 (m, 2H, CH Ph), 5.61 (br, 2H, NH_2), 3.38 (t, $^3J_{H\text{–}H}$ = 6.5, 2H, CH_2), 2.68 (t, $^3J_{H\text{–}H}$ = 6.5, 2H, CH_2). $^{13}C\{^1H\}$ APT NMR (75.48 MHz, $CDCl_3$, 298 K): δ 193.3 (COPh), 168.9 ($CONH_2$), 130.9 (C_q Ph), 127.8, 123.1, 122.6 (all CH Ph), 28.4 (CH_2), 23.9 (CH_2). IR (ATR, cm^{-1}): ν(N–H) 3403 (s), 3205 (s); ν(CO) 1683 (s), 1656 (s).

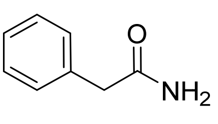

2-Phenylacetamide:[15] ^1H NMR (400.16 MHz, $CDCl_3$, 298 K): δ 7.29 (m, 3H, CH Ph), 7.20 (m, 2H, CH Ph), 5.62 (br, 2H, NH_2), 3.50 (s, 2H, CH_2). $^{13}C\{^1H\}$ APT NMR (75.48 MHz, $CDCl_3$, 298 K): δ 173.7 (CO), 134.9 (C_q Ph), 129.4, 129.0, 127.4 (all CH Ph), 43.3 (CH_2CO). IR (ATR, cm^{-1}): ν(N–H) 3349 (s), 3162 (s); ν(CO) 1633 (s).

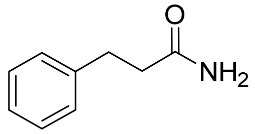

3-Phenylpropanamide:[19] ^{1}H NMR (300.13 MHz, $CDCl_3$, 298 K): δ 7.31 (m, 3H, CH Ph), 7.21 (m, 2H, CH Ph), 5.55 (br, 2H, NH_2), 2.99 (t, $^3J_{H-H}$ = 8.0, 2H, CH_2CO), 2.55 (t, $^3J_{H-H}$ = 8.1, 2H, CH_2Ph). $^{13}C\{^1H\}$ APT NMR (75.48 MHz, $CDCl_3$, 298 K): δ 174.5 (CO), 140.7 (C_q Ph), 128.6, 128.3, 126.3 (all CH Ph), 37.5 (CH_2CO), 31.4 (CH_2Ph). IR (ATR, cm^{-1}): ν(N–H) 3391 (s), 3180 (s); ν(CO) 1650 (s).

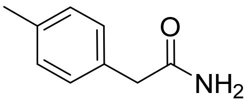

2-(*p*-tolyl)acetamide:[20] ^{1}H NMR (400.16 MHz, $CDCl_3$, 298 K): δ 7.09 (m, 4H, CH Ar), 5.44 (br, 2H, NH_2), 3.47 (s, 2H, CH_2CO), 2.27 (s, 3H, CH_3). $^{13}C\{^1H\}$ APT NMR (75.48 MHz, $CDCl_3$, 298 K): δ 173.9 (CO), 137.2 (C_q Ar), 131.8 (C_q Ar), 129.8, 129.3 (both CH Ar), 49.2 (CH_2CO), 21.1 (CH_3). IR (ATR, cm^{-1}): ν(N–H) 3346 (s), 3161 (s); ν(CO) 1630 (s)

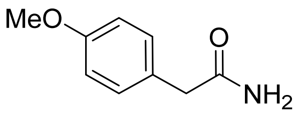

2-(4-Methoxyphenyl)acetamide:[21] ^{1}H NMR (300.13 MHz, $CDCl_3$, 298 K): δ 7.22 (m, 2H, CH Ar), 6.92 (m, 2H, CH Ar), 5.44 (br, 2H, NH_2), 3.83 (s, 3H, OCH_3), 3.55 (s, 2H, CH_2CO). $^{13}C\{^1H\}$ APT NMR (75.48 MHz, $CDCl_3$, 298 K): δ 173.9 (CO), 137.3 (C_q Ar), 130.5 (CH Ar), 126.9 (C_q Ar), 114.5 (CH Ar), 55.3 (OCH_3), 42.4 (CH_2CO). IR (ATR, cm^{-1}): ν(N–H) 3345 (s), 3158 (s); ν(CO) 1634 (s).

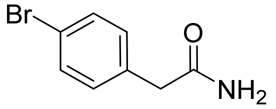

2-(4-Bromophenyl)acetamide:[22] ^{1}H NMR (300.13 MHz, $CDCl_3$, 298 K): δ 7.51 (m, 2H, CH Ar), 7.19 (m, 2H, CH Ar), 5.44 (br, 2H, NH_2), 3.56 (s, 2H, CH_2CO). $^{13}C\{^1H\}$ APT NMR (75.48 MHz, $CDCl_3$, 298 K): δ 172.5 (CO), 133.7 (C_q Ar), 132.1, 131.1 (both CH Ar), 121.5 (BrC_q Ar), 42.5 (CH_2CO). IR (ATR, cm^{-1}): ν(N–H) 3406 (s); ν(CO) 1651 (s)

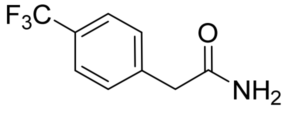

2-(4-Trifluorophenyl)acetamide:[22] ^{1}H NMR (300.13 MHz, $CDCl_3$, 298 K): δ 7.64 (m, 2H, CH Ar), 7.44 (m, 2H, CH Ar), 5.51 (br, 2H, NH_2), 3.66 (s, 1H, CHCO). ^{19}F NMR (376.49 MHz, $CDCl_3$, 298 K): 62.58 (CF_3). $^{13}C\{^1H\}$ APT NMR (75.48 MHz, $CDCl_3$, 298 K): δ 172.4 (CO), 138.7 (C_q Ar), 129.7 (CH Ar), 125.9 (q, $^4J_{F-H}$ = 3.8, CH Ar), 122.3 (q, $^3J_{F-H}$ = 18.9, C_q Ar), 42.8 (CHCO). The CF_3 resonance was not observed. IR (ATR, cm^{-1}): ν(N–H) 3354 (s), 3164 (s); ν(CO) 1634 (s).

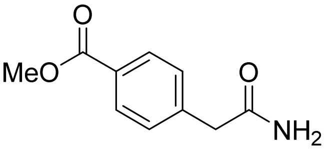

Methyl-4-(2-amino-2-oxoethyl)benzoate:[23] ^{1}H NMR (300.13 MHz, $CDCl_3$, 298 K): δ 8.02 (m, 2H, CH Ar), 7.37 (m, 2H, CH Ar), 5.55 (br, 2H, NH_2), 3.91 (s, 3H, OCH_3), 3.65

(s, 2H, CH_2CO). $^{13}C\{^1H\}$ APT NMR (75.48 MHz, $CDCl_3$, 298 K): δ 172.3 (CO), 166.7 (CO_2Me), 139.9 (C_q Ar), 130.2, 129.4 (both CH Ar), 129.4 (C_q Ar), 52.2 (OCH_3), 43.1 (CH_2CO). IR (ATR, cm^{-1}): ν(N–H) 3386 (s), 3202 (s); ν(CO) 1720 (s), 1650(s).

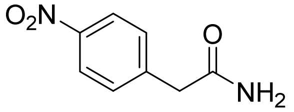

4-Nitrophenylacetamide:[24] ^1H NMR (300.13 MHz, $CDCl_3$, 298 K): δ 8.25 (m, 2H, CH Ar), 7.51 (m, 2H, CH Ar), 5.43 (br, 2H, NH_2), 3.71 (s, 2H, CH_2CO). IR (ATR, cm^{-1}): ν(N–H) 3411 (s), 3156 (s); ν(CO) 1624 (s); ν(NO) 1343(s).

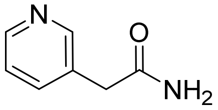

(Pyridin-3-yl)acetamide:[15] ^1H NMR (300.13 MHz, $CDCl_3$, 298 K): δ 8.53 (br, 2H, CH Py), 7.68 (br, 1H, CH Py), 7.31 (br, 2H, CH Py), 5.89 (br, 2H, NH_2), 3.59 (s, 1H, CH). $^{13}C\{^1H\}$ APT NMR (75.48 MHz, $CDCl_3$, 298 K): δ 172.3 (CO), 150.3, 148.6, 136.9 (all CH Py), 130.6 (C_q Py), 123.7 (CH Py), 40.0 (CH_2). IR (ATR, cm^{-1}): ν(N–H) 3348 (s), 3088 (s); ν(CO) 1671 (s).

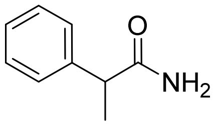

2-Phenylpropanamide:[25] ^1H NMR (300.13 MHz, $CDCl_3$, 298 K): δ 7.39-7.28 (m, 5H, CH Ph), 5.47 (br, 2H, NH_2), 3.61 (q, $^3J_{H\text{–}H}$ = 7.2, 1H, CHCO), 1.55 (d, $^3J_{H\text{–}H}$ = 7.2, 3H, CH_3). $^{13}C\{^1H\}$ APT NMR (75.48 MHz, $CDCl_3$, 298 K): δ 176.7 (CO), 141.3 (C_q Ph), 129.0, 127.6, 127.4 (all CH Ph), 46.6 (CHCO), 18.3 (CH_3). IR (ATR, cm^{-1}): ν(N–H) 3354 (s), 3176 (s); ν(CO) 1632 (s).

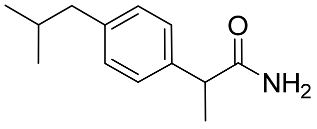

2-(4-Isobutylphenyl)propanamide:[26] ^1H NMR (300.13 MHz, $CDCl_3$, 298 K): δ 7.14 (m, 2H, CH Ar), 7.05 (m, 2H, CH Ar), 5.44 (br, 2H, NH_2), 3.50 (q, $^3J_{H\text{–}H}$ = 7.2, 1H, CHCO), 2.38 (d, $^3J_{H\text{–}H}$ = 7.2, 3H, CH_3), 1.77 (m, 1H, CH ^iPr), 1.44 (d, $^3J_{H\text{–}H}$ = 7.2, 3H, CH_3), 0.83 (d, $^3J_{H\text{–}H}$ = 6.6, 6H, CH_3 ^iPr). $^{13}C\{^1H\}$ APT NMR (75.48 MHz, $CDCl_3$, 298 K): δ 177.0 (CO), 140.9, 138.5 (both C_q Ar), 129.7, 127.3 (both CH Ar), 46.3 (CHCO), 45.0 (CH_2), 30.2 (CH), 22.4, 18.3 (both CH_3). IR (ATR, cm^{-1}): ν(N–H) 3346 (s), 3168 (s); ν(CO) 1630 (s).

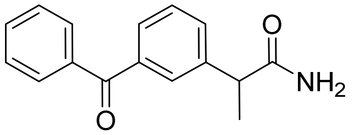

2-(3-Benzoylphenyl)propanamide:[27] ^1H NMR (300.13 MHz, $CDCl_3$, 298 K): δ 7.78 (m, 3H, CH Ar), 7.67 (m, 1H, CH Ar), 7.59 (m, 2H, CH Ar), 7.49 (m, 3H, CH Ar), 5.74 (br, 2H, NH_2), 3.70 (q, $^3J_{H\text{–}H}$ = 7.0, 1H, CHCO), 1.55 (d, $^3J_{H\text{–}H}$ = 7.0, 3H, CH_3). $^{13}C\{^1H\}$ APT NMR (75.48 MHz, $CDCl_3$, 298 K): δ 196.5 (CO), 176.0 ($CONH_2$), 141.8, 138.1,

137.4 (C_q Ar), 132.6, 131.5, 130.0, 129.2, 129.1, 128.8, 128.4 (all CH Ar), 46.4 (CH), 18.5 (CH_3). IR (ATR, cm^{-1}): ν(N–H) 3342 (s), 3190 (s); ν(CO) 1651 (s).

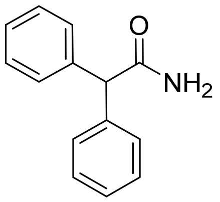

2,2-Diphenylacetamide:[28] ^1H NMR (300.13 MHz, $CDCl_3$, 298 K): δ 7.39-7.28 (m, 10H, CH Ph), 5.71 (br, 2H, NH_2), 4.98 (s, 1H, CH). $^{13}C\{^1H\}$ APT NMR (75.48 MHz, $CDCl_3$, 298 K): δ 174.4 (CO), 139.2 (C_q Ph), 128.9, 128.8, 127.4 (CH Ph), 58.8 (CH). IR (ATR, cm^{-1}): ν(N–H) 3387 (s), 3173 (s); ν(CO) 1651 (s).

References

(1) Aracama, M.; Esteruelas, M. A.; Lahoz, F. J.; López, J. A.; Meyer, U.; Oro, L. A.; Werner, H. Synthesis, Reactivity, Molecular Structure, and Catalytic Activity of the Novel Dichlorodihydridoosmium(IV) Complexes $OsH_2Cl_2(PR_3)_2$ (PR_3 = P-*i*-Pr_3, PMe-*t*-Bu_2). *Inorg. Chem.* **1991**, *30*, 288−293.

(2) Blessing, R. H. An Empirical Correction for Absorption Anisotropy. *Acta Crystallogr.* **1995**, *A51*, 33. SADABS: Area-detector absorption correction; Bruker-AXS, Madison, WI, 1996.

(3) SHELXL-2016/6. Sheldrick, G. M. A short history of SHELX. *Acta Cryst.* **2008**, *A64*, 112-122.

(4) (a) Lee, C.; Yang, W.; Parr, R. G. Development of the Colle-Salvetti correlation-energy formula into a functional of the electron density. *Phys. Rev. B* **1988**, *37*, 785–789. (b) Becke, A. D. Density-functional thermochemistry .III. The role of exact exchange. *J. Chem. Phys.* **1993**, *98*, 5648–5652. (c) Stephens, P. J.; Devlin, F. J.; Chabalowski, C. F.; Frisch, M. J. Ab Initio Calculation of Vibrational Absorption and Circular Dichroism Spectra Using Density Functional Force Fields *J. Phys. Chem.* **1994**, *98*, 11623–11627.

(5) Grimme, S.; Antony, J.; Ehrlich, S.; Krieg, H. A consistent and accurate ab initio parametrization of density functional dispersion correction (DFT-D) for the 94 elements H-Pu. *J. Chem. Phys*., **2010**, *132*, 154104-154123.

(6) Gaussian 09, Revision D.01, Frisch, M. J.; Trucks, G. W.; Schlegel H. B.; Scuseria, G. E.; Robb, M. A.; Cheeseman, J. R.; Scalmani, G.; Barone, V.; Mennucci, B.; Petersson, G. A.; Nakatsuji, H.; Caricato, M.; Li, X.; Hratchian, H. P.; Izmaylov, A. F.; Bloino, J.; Zheng, G.; Sonnenberg, J. L.; Hada, M.; Ehara, M.; Toyota, K.; Fukuda, R.; Hasegawa, J.; Ishida, M.; Nakajima, T.; Honda, Y.; Kitao, O.; Nakai, H.; Vreven, T.; Montgomery, J. A.; Peralta, Jr., J. E.; Ogliaro, F.; Bearpark, M.; Heyd, J. J.; Brothers, E.; Kudin, K. N.; Staroverov, V. N.; Keith, T.; Kobayashi, R.; Normand, J.; Raghavachari, K.; Rendell, A.; Burant, J. C.; Iyengar, S. S.; Tomasi, J.; Cossi, M.; Rega, N.; Millam, J. M.; Klene, M.; Knox, J. E.; Cross, J. B.; Bakken, V.; Adamo, C.; Jaramillo, J.; Gomperts, R.; Stratmann, R. E.; Yazyev, O.; Austin, A. J.; Cammi, R.; Pomelli, C.; Ochterski, J. W.; Martin, R. L.; Morokuma, K.; Zakrzewski, V. G.; Voth, G. A.; Salvador, P.; Dannenberg, J. J.; Dapprich, S.; Daniels, A. D.; Farkas, O.; Foresman, J. B.; Ortiz, J. V.; Cioslowski, J.; Fox, D. J. Gaussian, Inc., Wallingford CT, 2013.

(7) Andrea, D.; Haeussermann, U. M.; Dolg, M.; Stoll, H.; Preuss, H. Energy-adjustedab initio pseudopotentials for the second and third row transition elements *Theor. Chim. Acta* **1990**, *77*, 123–141.

(8) Ehlers, A. W.; Bohme, M.; Dapprich, S.; Gobbi, A.; Hollwarth, A.; Jonas, V.; Kohler, K. F.; Stegmann, R.; Veldkamp, A.; Frenking, G. A set of f-polarization functions for pseudo-potential basis sets of the transition metals SC-Cu, Y-Ag and La-Au. *Chem. Phys. Lett.* **1993**, *208*, 111–114.

(9) (a) Hehre, W. J.; Ditchfield, R.; Pople, J. A. Self-Consistent Molecular Orbital Methods. XII. Further Extensions of Gaussian-Type Basis Sets for Use in Molecular Orbital Studies of Organic Molecules. *J. Chem. Phys.* **1972**, *56*, 2257–2261. (b) Francl, M. M.; Pietro, W. J.; Hehre, W. J.; Binkley, J. S.; Gordon, M. S.; DeFrees, D. J.; Pople, J. A. Self-consistent molecular orbital methods. XXIII. A polarization-type basis set for second-row elements. *J. Chem. Phys.* **1982**, *77*, 3654–3665.

(10) Marenich, A. V.; Cramer, C. J.; Truhlar, D. G. Universal Solvation Model Based on Solute Electron Density and on a Continuum Model of the Solvent Defined by the Bulk Dielectric Constant and Atomic Surface Tensions *J. Phys. Chem. B* **2009**, *113*, 6378–6396.

(11) Ali, M. A.; Punniyamurthy, T. Palladium-Catalyzed One-Pot Conversion of Aldehydes to Amides. *Adv. Synth. Catal.* **2010**, *352*, 288-292.

(12) Kuzma, P. C.; Brown, L. E.; Harris, T. M. Generation of the Dianion of *N*-(Trimethylsilyl)acetamide and Reaction of the Dianion with Electrophilic Reagents. *J. Org. Chem.* **1984**, *49*, 2015-2018.

(13) Page, P. C. B.; Rosenthal, S.; Williams, R. V. A New, One-Pot Synthesis of Primary 2-Alkynamides. *Synthesis* **1988**, 621-623.

(14) Coxon, B.; Fatiadi, A. J.; Sniegoski, L. T.; Hertz, H. S.; Schaffer, R. A Novel Acylative Degradation of Uric Acid. Carbon-13 Nuclear Magnetic Resonance Studies of Uric Acid and Its Degradation Products. *J. Org. Chem.* **1977**, *42*, 3132-3140.

(15) Bonne, D.; Dekhane, M.; Zhu, J. Mild Oxidative One-Carbon Homologation of Aldehyde to Amide. *J. Am. Chem. Soc.* **2005**, *127*, 6926-6927.

(16) Kaufmann, D.; Bialer, M.; Shimshoni, J. A.; Devor, M.; Yagen, B. Synthesis and Evaluation of Antiallodynic and Anticonvulsant Activity of Novel Amide and Urea Derivatives of Valproic Acid Analogues. *J. Med. Chem.* **2009**, *52*, 7236-7248.

(17) Breno, K. L.; Pluth, M. D.; Tyler, D. R. Organometallic Chemistry in Aqueous Solution. Hydration of Nitriles to Amides Catalyzed by a Water-Soluble Molybdocene, $(MeCp)_2Mo(OH)(H_2O)^+$. *Organometallics* **2003**, *22*, 1203-1211.

(18) Kouser, F.; Sharma, V. K.; Rizvi, M.; Sultan, S.; Chalotra, N.; Gupta, V. K.; Nandi, U.; Shah, B. A. Stereoselective synthesis of 3,4-di-substituted mercaptolactones viaphotoredox-catalyzed radical addition of thiophenols. *Tetrahedron Lett.* **2018**, *59*, 2161–2166.

(19) Brinchi, L.; Chiavini, L.; Goracci, L.; Di Profio, P.; Germani, R. Efficient Hydrolysis of Nitriles to Amides with Hydroperoxide Anion in Aqueous Surfactant Solutions as Reaction Medium. *Lett. Org. Chem.* **2009**, *6*, 175-179.

(20) BIORAD: https://spectrabase.com/spectrum/7S7BfqYTYpz.

(21) Chen, H.; Dai, W.; Chen, Y.; Xu, Q.; Chen, J.; Yu, L.; Zao, Y.; Ye, M.; Pan, Y. Efficient and selective nitrile hydration reactions in water catalyzed by an unexpected

dimethylsulfinyl anion generated in situ from CsOH and DMSO. *Green Chem.* **2014**, *16*, 2136-2141.

(22) Yoshimura, A.; Middleton, K. R.; Luedtke, M. W.; Zhu, C.; Zhdankin, V. V. Hypervalent Iodine Catalyzed Hofmann Rearrangement of Carboxamides Using Oxone as Terminal Oxidant. *J. Org. Chem.* **2012**, *77*, 11399-11404.

(23) Shen, W.; Kunzer, A. A Facile One-Carbon Homologation of Aryl Aldehydes to Amides. *Org. Lett.* **2002**, *4*, 1315–1317.

(24) Veisi, H.; Maleki, B.; Hamelian, M.; Ashrafi, S. S. Chemoselective hydration of nitriles to amides using hydrated ionic liquid (IL) tetrabutylammonium hydroxide (TBAH) as a green catalyst. *RSC Adv.* **2015**, *5*, 6365–6371.

(25) Liu, C.; He, C.; Shi, W.; Chen, M.; Lei, A. Ni-Catalyzed Mild Arylation of α-Halocarbonyl Compounds with Arylboronic Acids. *Org. Lett.* **2007**, *26*, 5601–5604.

(26) García-Álvarez, R.; Francos J.; Crochet, P.; Cadierno, V. Ibuprofenamide: A convenient method of synthesis by catalytic hydration of 2-(4-isobutylphenyl)propionitrile in pure aqueous medium. *Tetrahedron Lett.* **2011**, *52*, 4218–4220.

(27) **CAS:** 59512-16-2

(28) Kitamura, M.; Miyagawa, S.; Okauchi, T. Synthesis of α,α-diarylacetamides from benzyl aryl ketones using 2-azido-1,3-dimethylimidazolinium hexafluorophosphate. *Tetrahedron Lett.* **2011,** *52*, 3158-3161.

^{1}H NMR spectra of the reaction crude of the catalytic nitrile hydrations, ^{1}H, $^{13}C\{^{1}H\}$ APT NMR and IR spectra of the amides

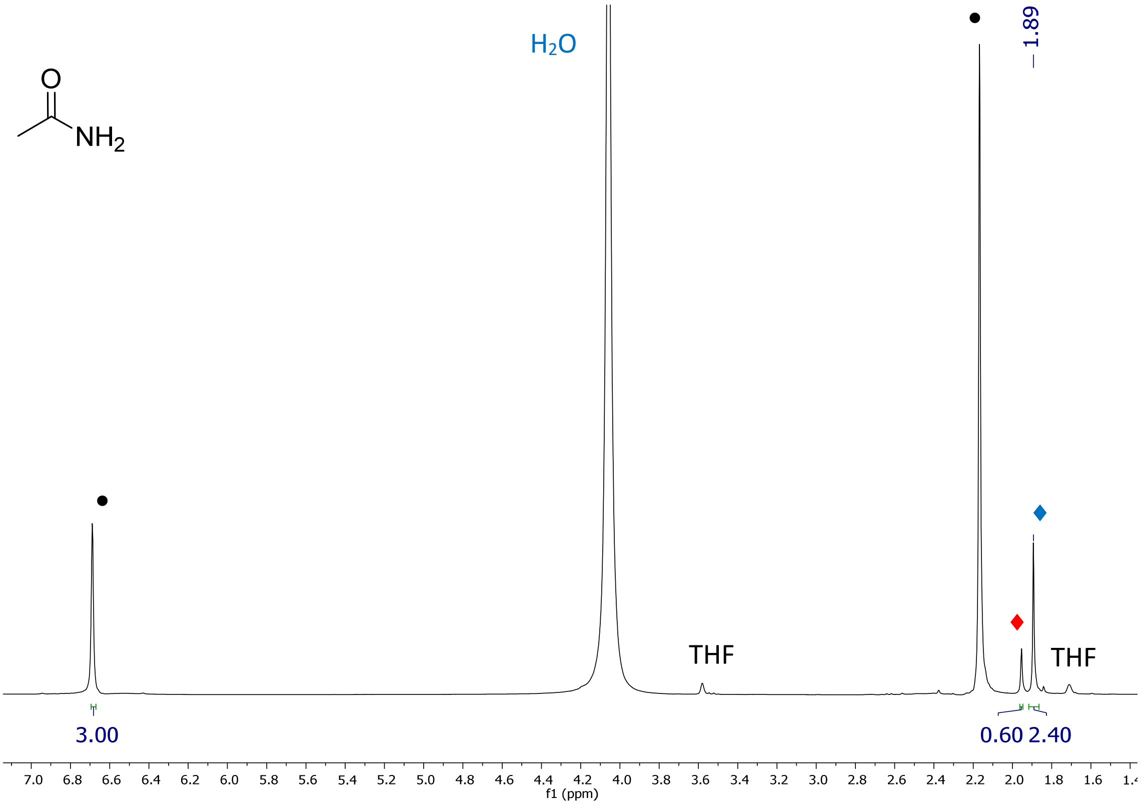

Figure S1. ^{1}H NMR (300.13 MHz, THF-d_8, 298 K) spectrum of the reaction mixture of the hydration of acetonitrile (♦): formation of acetamide (♦). • Mesitylene.

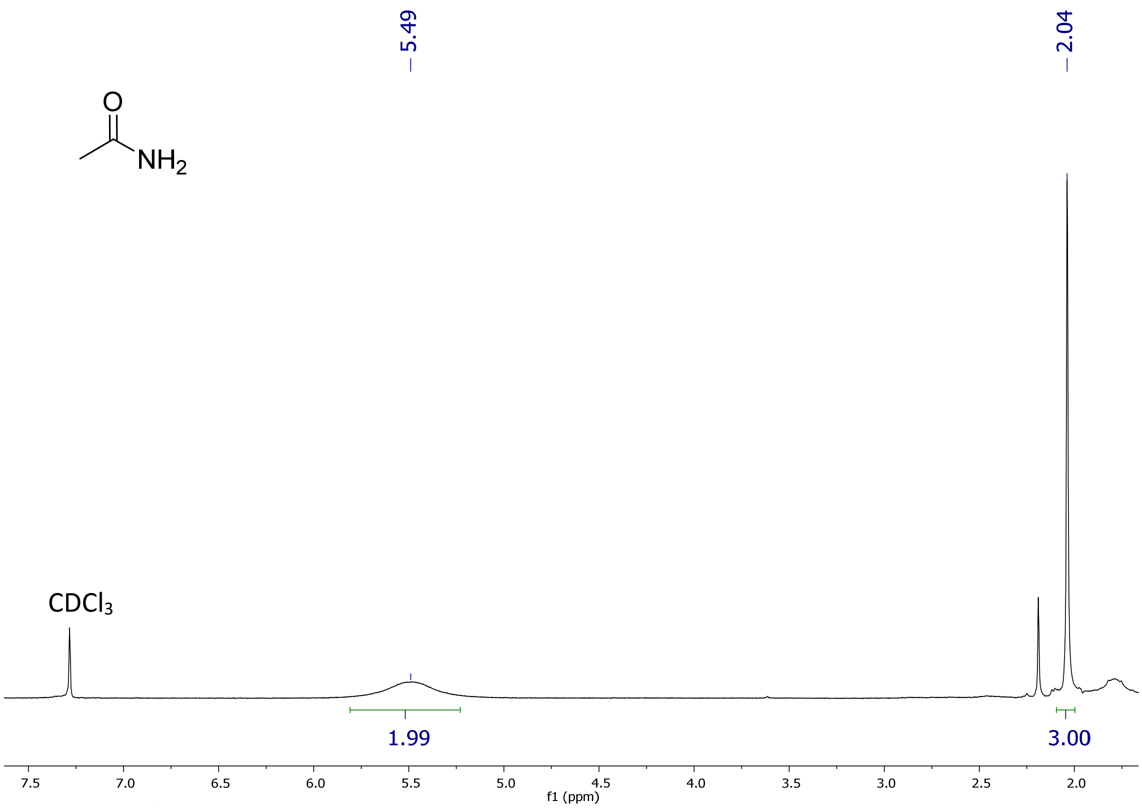

Figure S2. ^{1}H NMR (300.13 MHz, $CDCl_3$, 298 K) spectrum of acetamide.

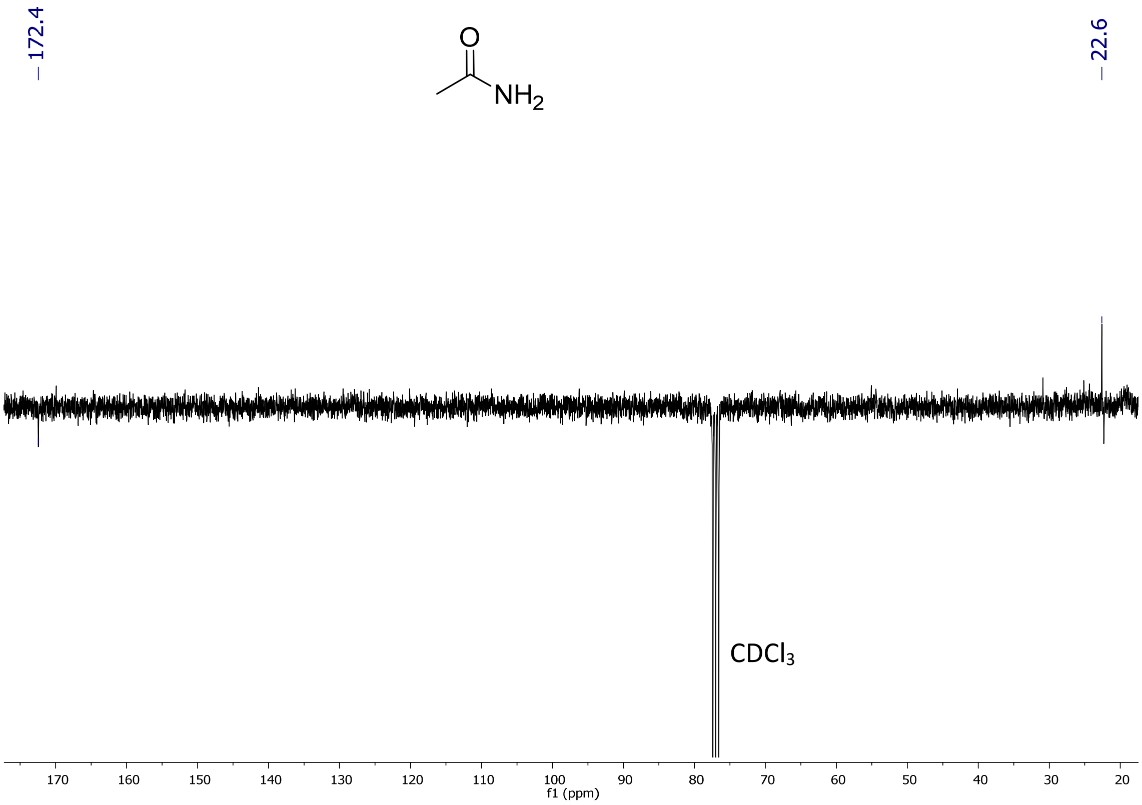

Figure S3. $^{13}C\{^{1}H\}$ APT NMR (75.48 MHz, $CDCl_3$, 298 K) spectrum of acetamide.

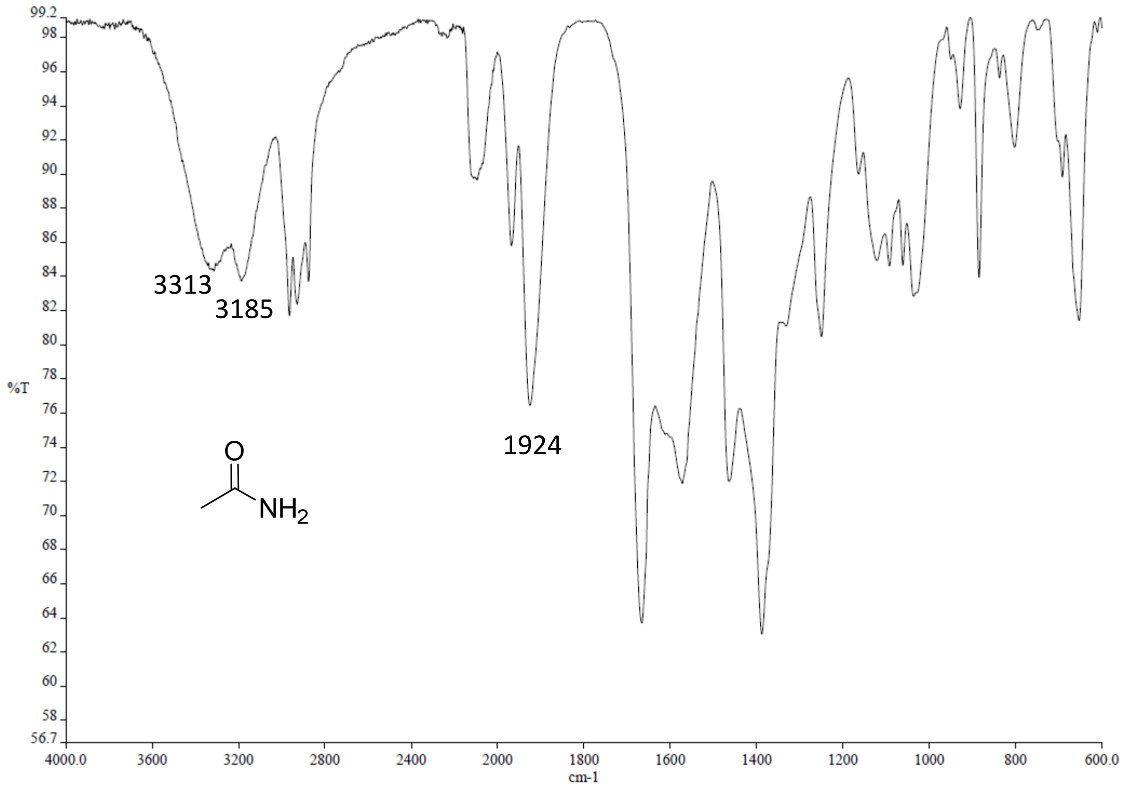

Figure S4. IR ATR spectrum of acetamide.

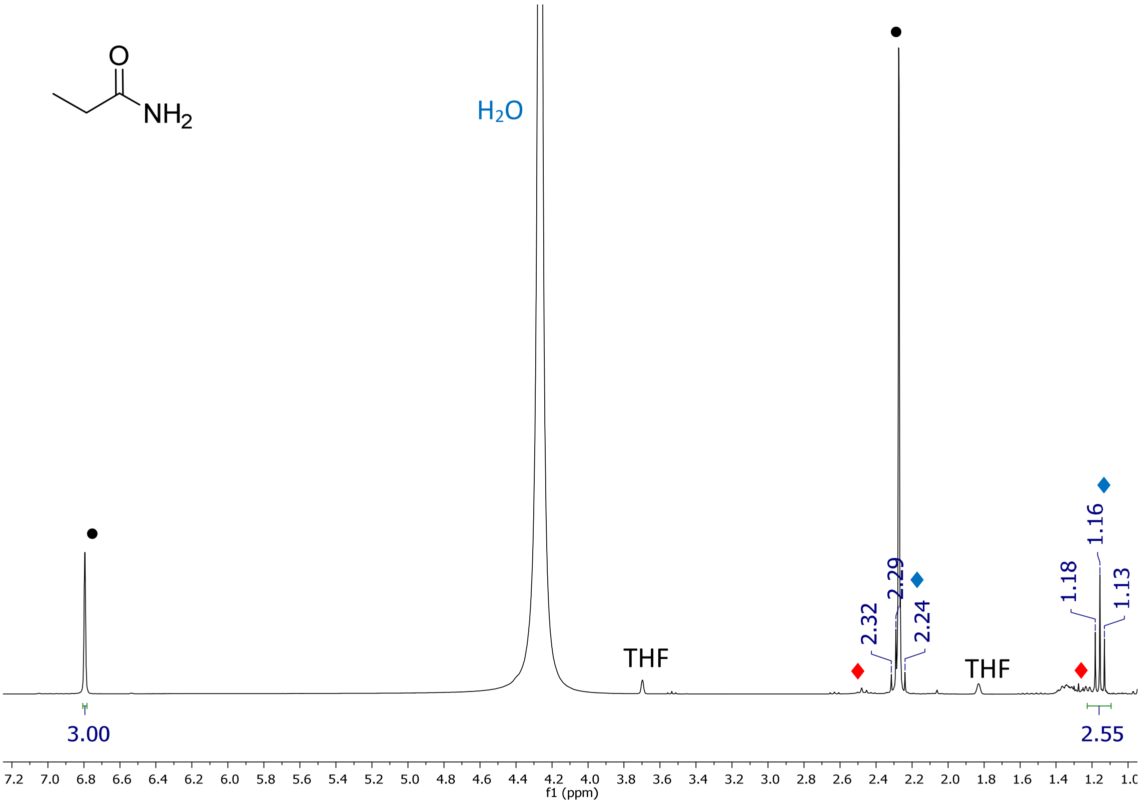

Figure S5. ^{1}H NMR (300.13 MHz, THF-d_8, 298 K) spectrum of the reaction mixture of the hydration of propionitrile (♦): formation of propionamide (♦). • Mesitylene.

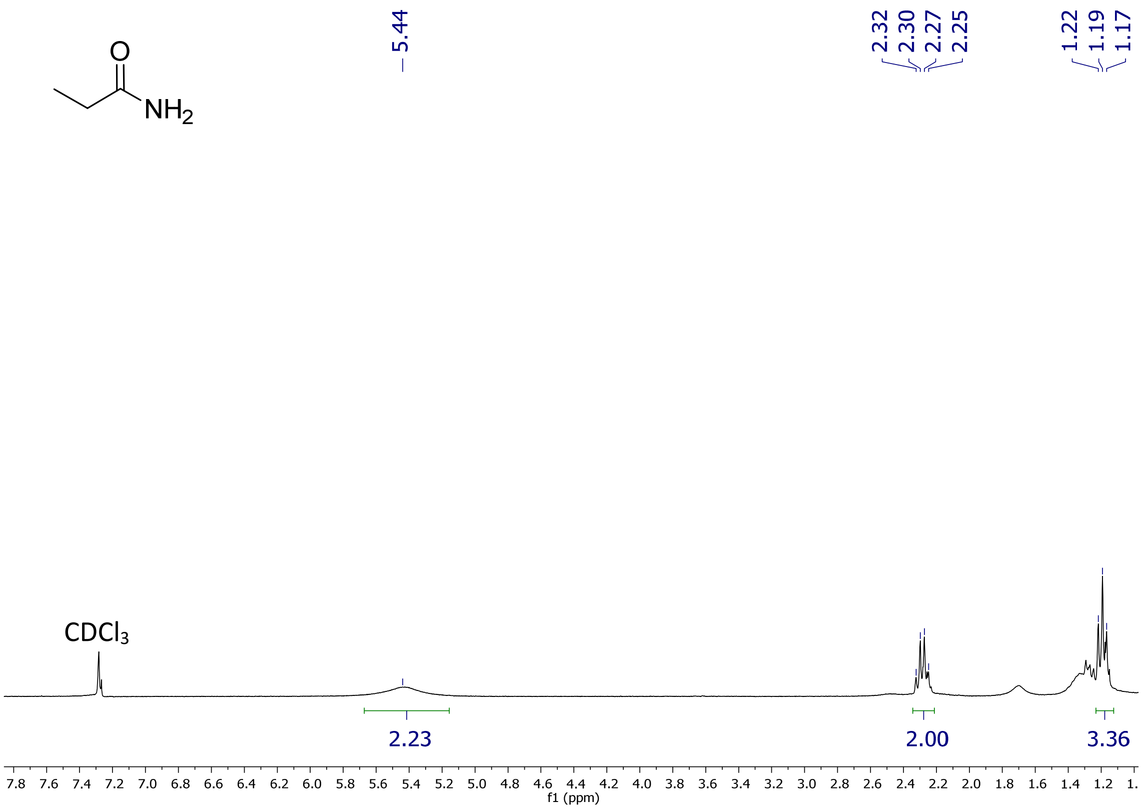

Figure S6. ^{1}H NMR (300.13 MHz, $CDCl_3$, 298 K) spectrum of propanamide.

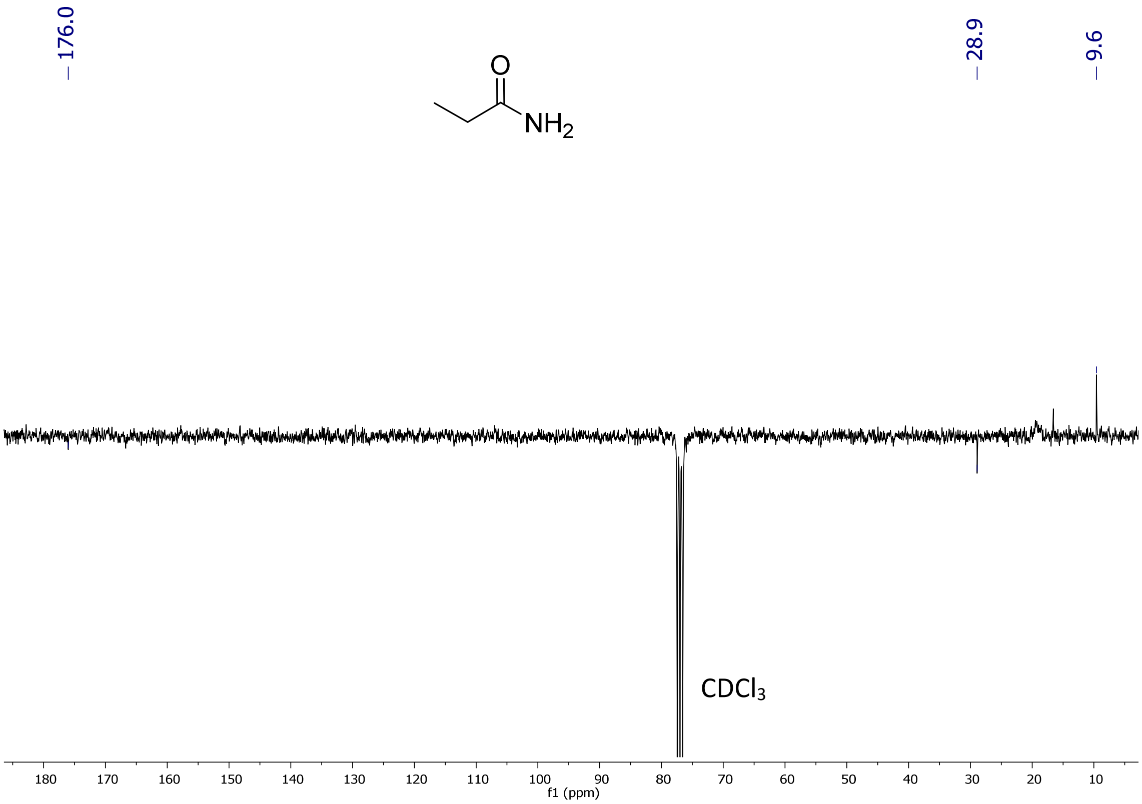

Figure S7. $^{13}C\{^{1}H\}$ APT NMR (75.48 MHz, $CDCl_3$, 298 K) spectrum of propanamide.

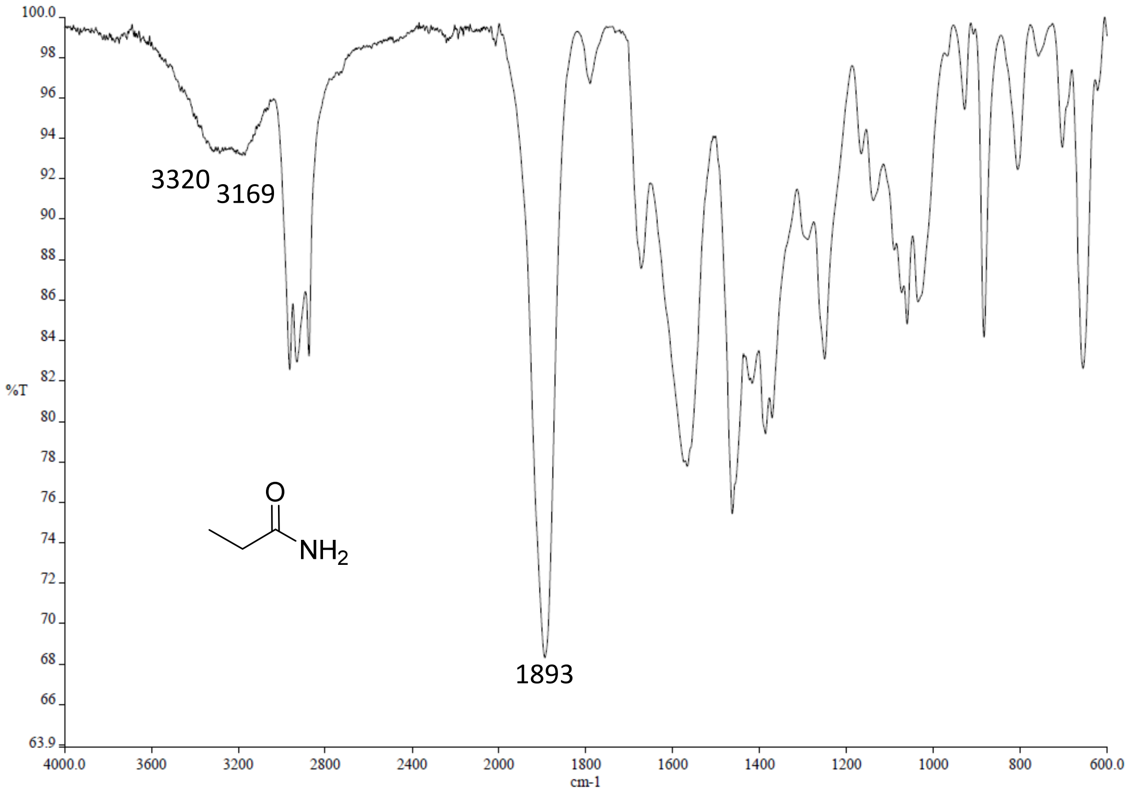

Figure S8. IR ATR spectrum of propanamide.

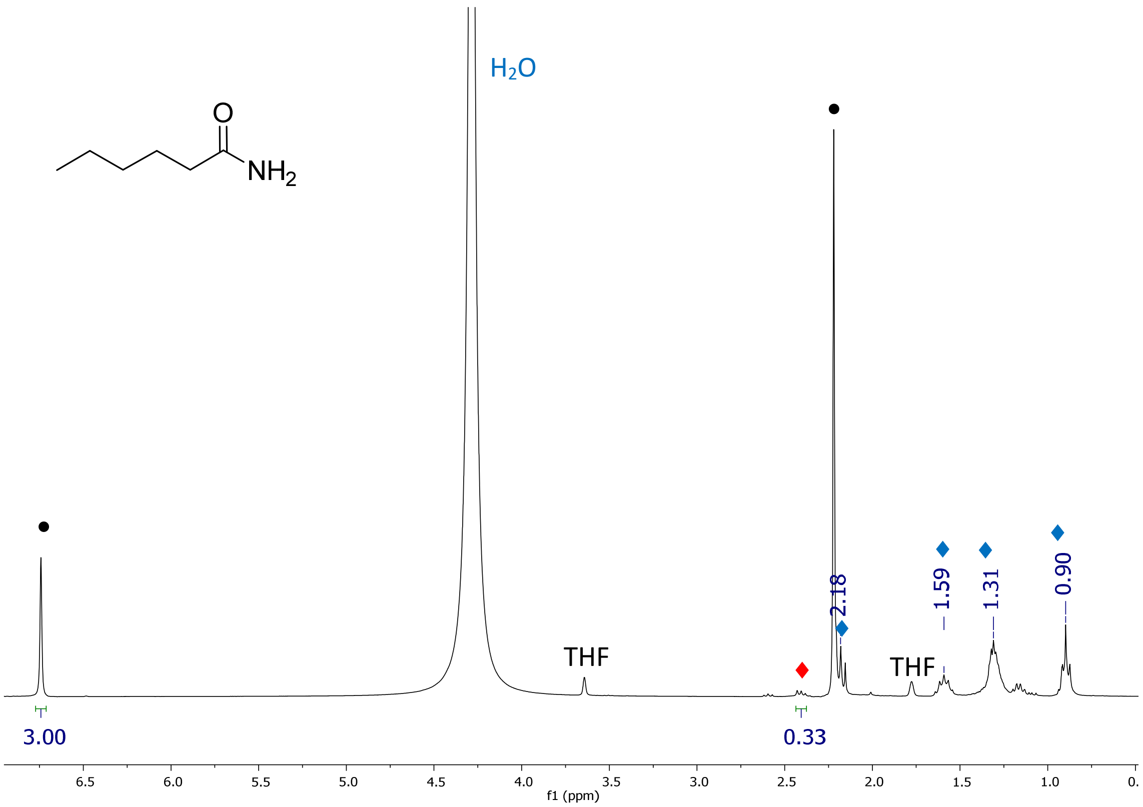

Figure S9. ^{1}H NMR (300.13 MHz, THF-d_8, 298 K) spectrum of the reaction mixture of the hydration of hexanenitrile (♦): formation of hexanamide (♦). • Mesitylene.

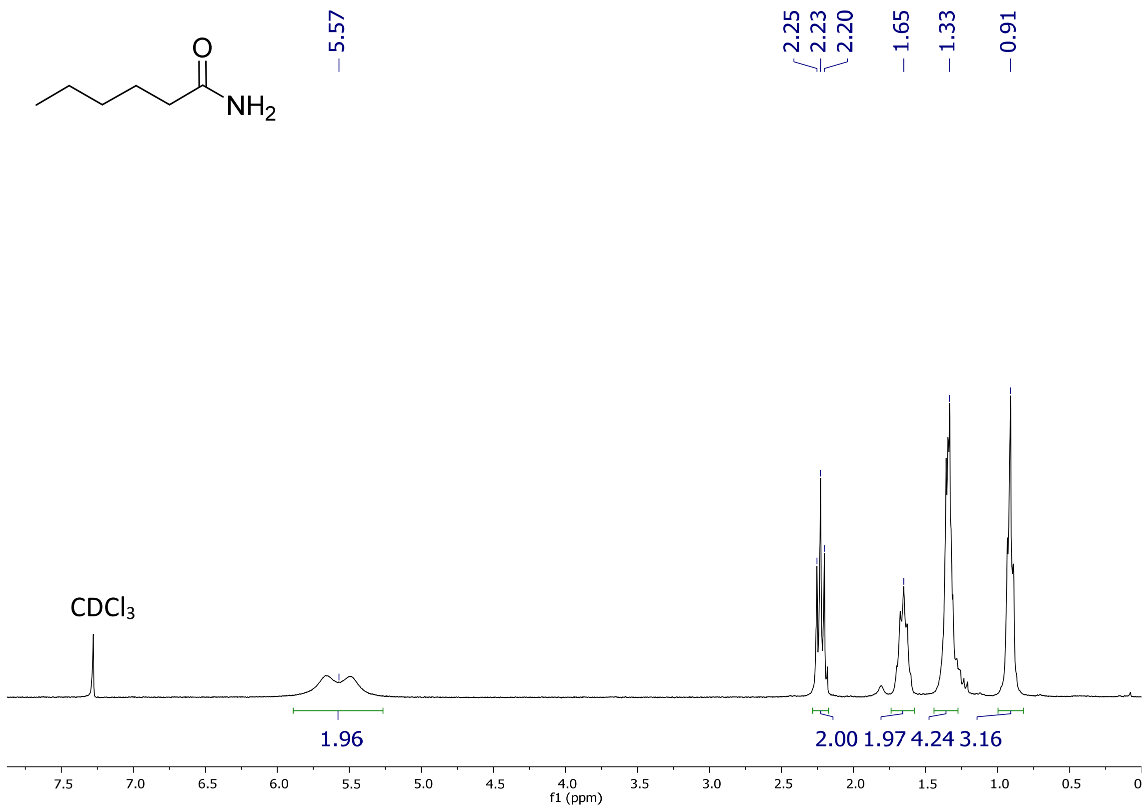

Figure S10. ^{1}H NMR (300.13 MHz, $CDCl_3$, 298 K) spectrum of hexanamide.

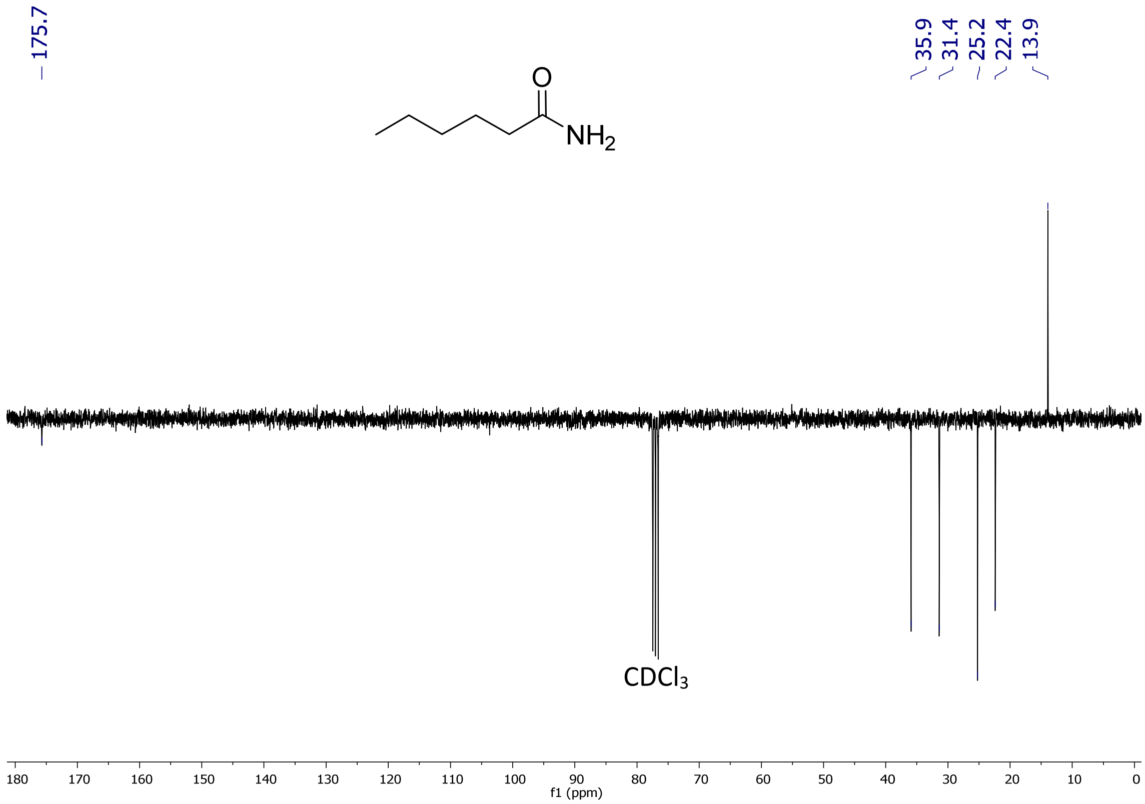

Figure S11. $^{13}C\{^{1}H\}$ APT NMR (75.48 MHz, $CDCl_3$, 298 K) spectrum of hexanamide.

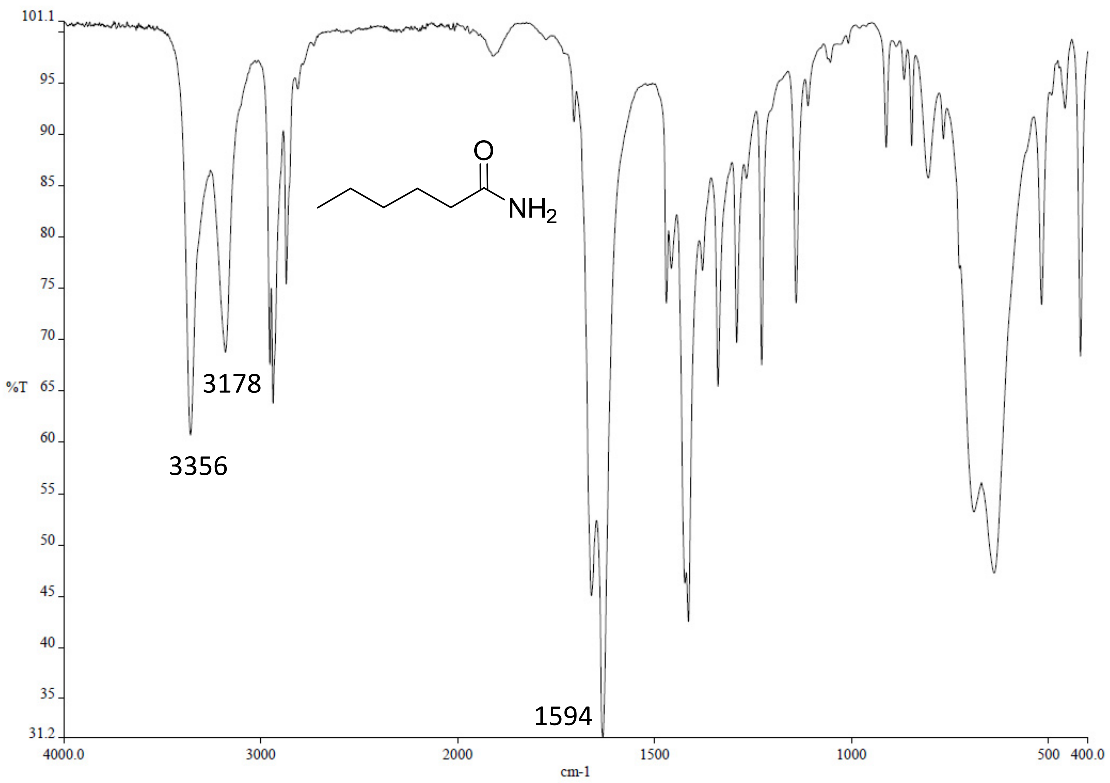

Figure S12. IR ATR spectrum of hexanamide.

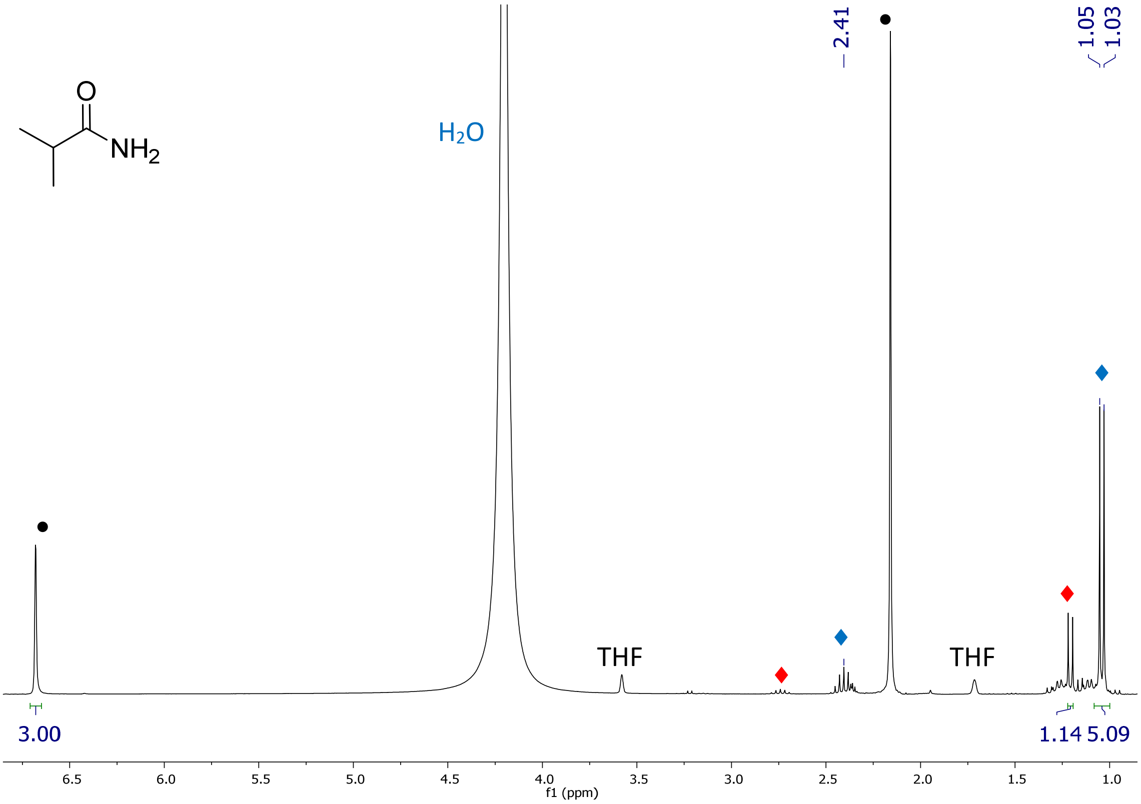

Figure S13. ^{1}H NMR (300.13 MHz, THF-*d*8, 298 K) spectrum of the reaction mixture of the hydration of 2-methylpropionitrile (♦): formation of 2-methylpropanamide (♦). • Mesitylene.

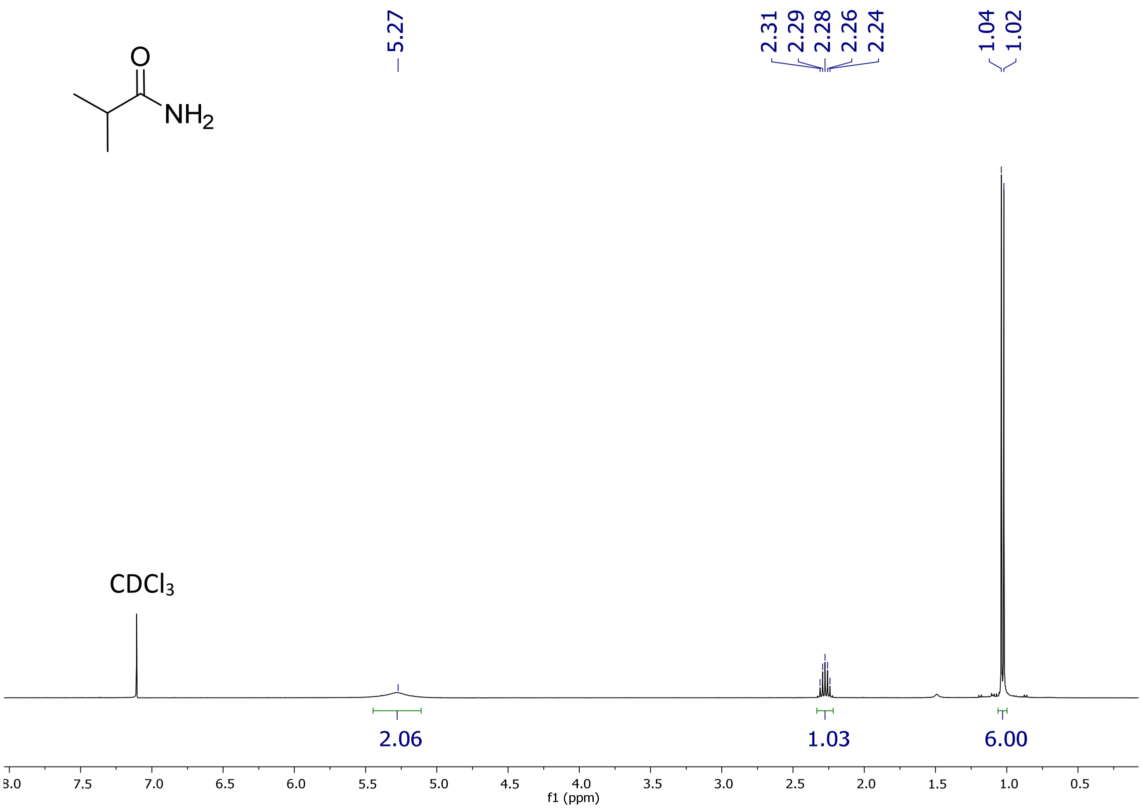

Figure S14. ^{1}H NMR (400.16 MHz, $CDCl_3$, 298 K) spectrum of 2-methylpropanamide.

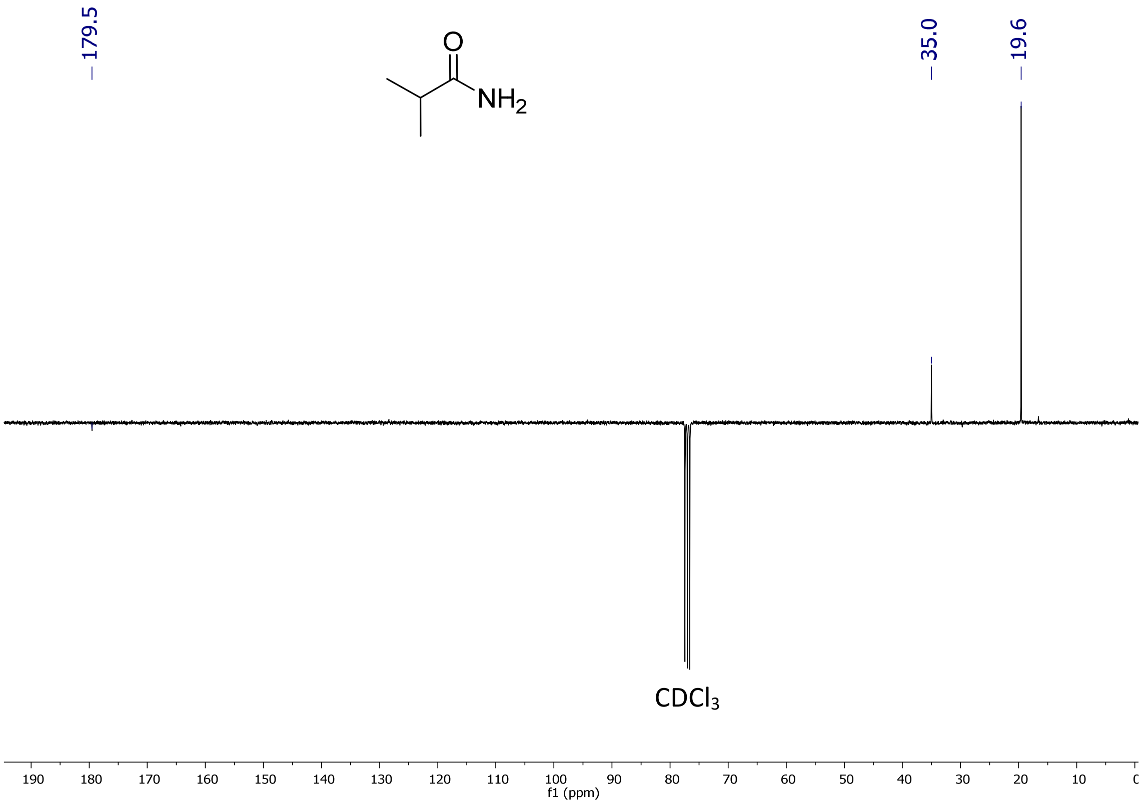

Figure S15. $^{13}C\{^{1}H\}$ APT NMR (75.48 MHz, $CDCl_3$, 298 K) spectrum of 2-methylpropanamide.

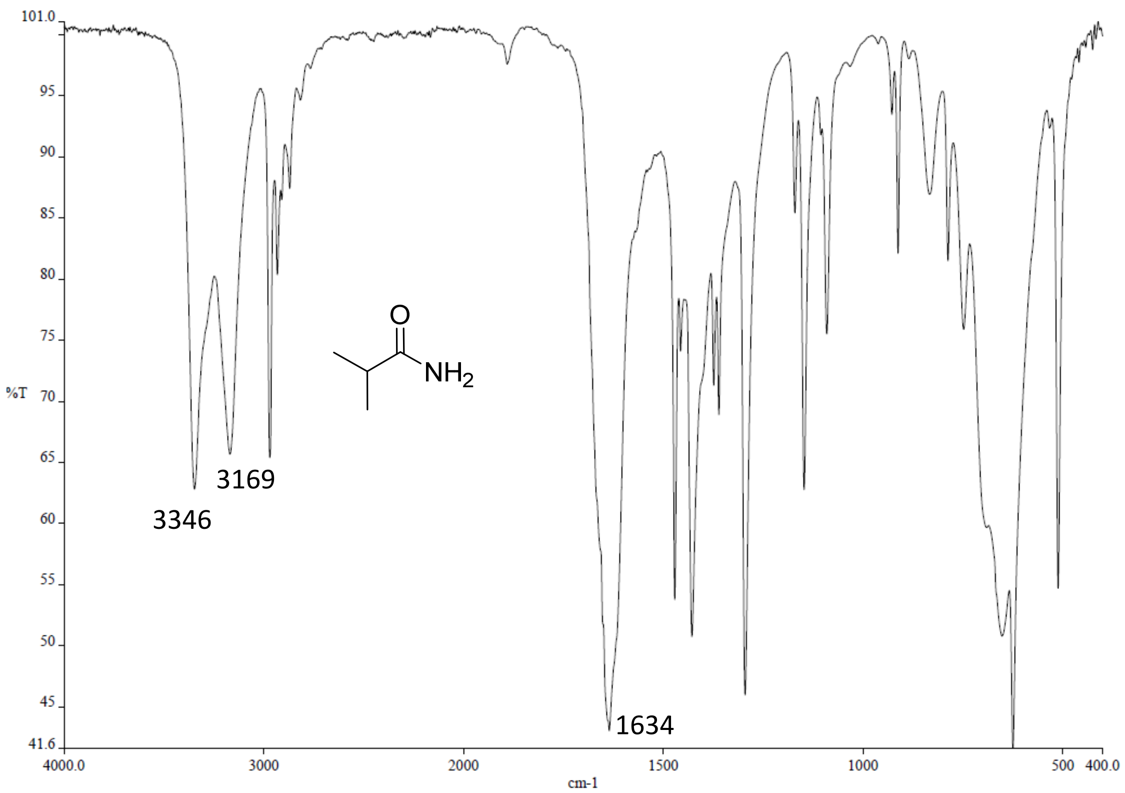

Figure S16. IR ATR spectrum of 2-methylpropanamide.

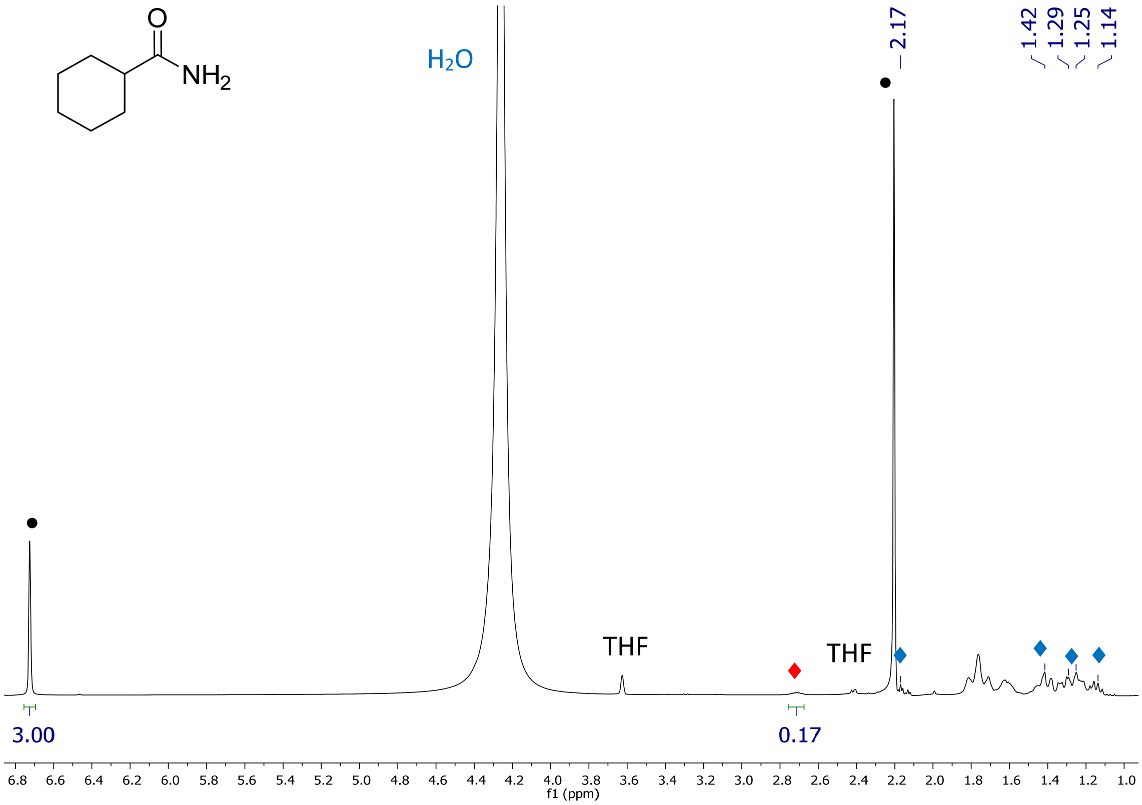

Figure S17. ^{1}H NMR (300.13 MHz, THF-d_8, 298 K) spectrum of the reaction mixture of the hydration of cyclohexanecarbonitrile (♦): formation of cyclohexanecarbamide (♦). • Mesitylene.

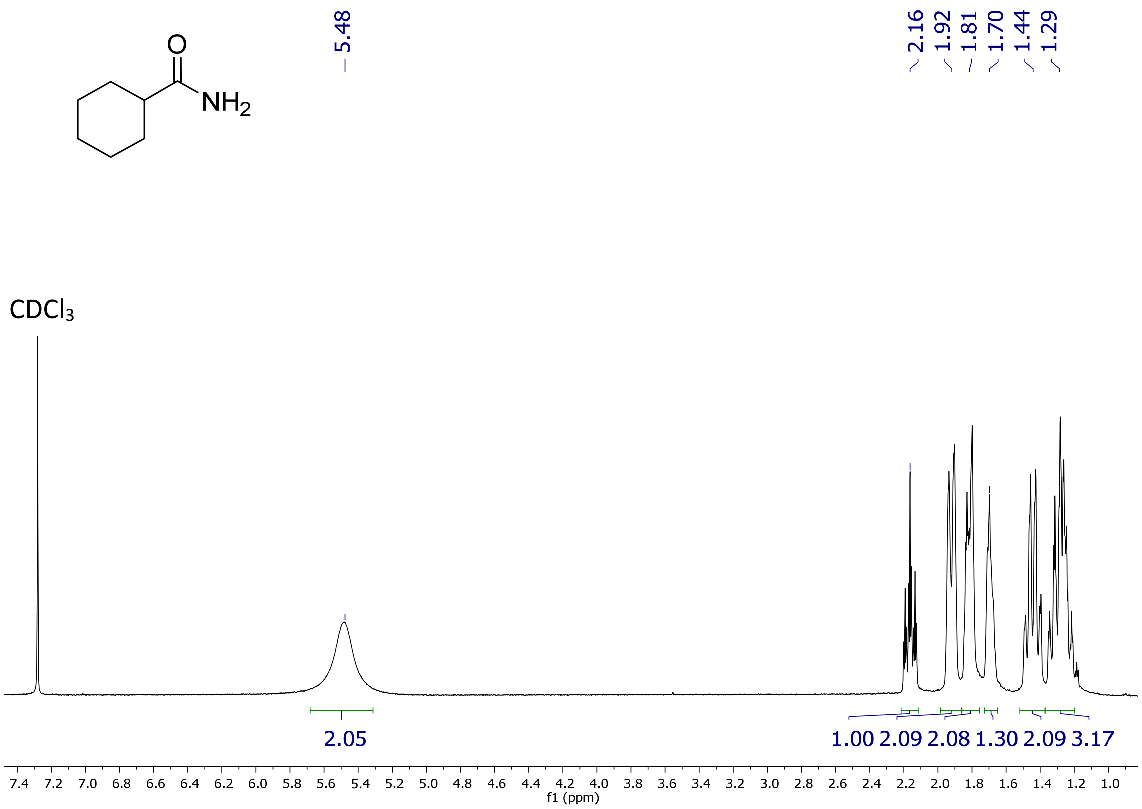

Figure S18. ^{1}H NMR (400.16 MHz, $CDCl_3$, 298 K) spectrum of cyclohexanecarbamide.

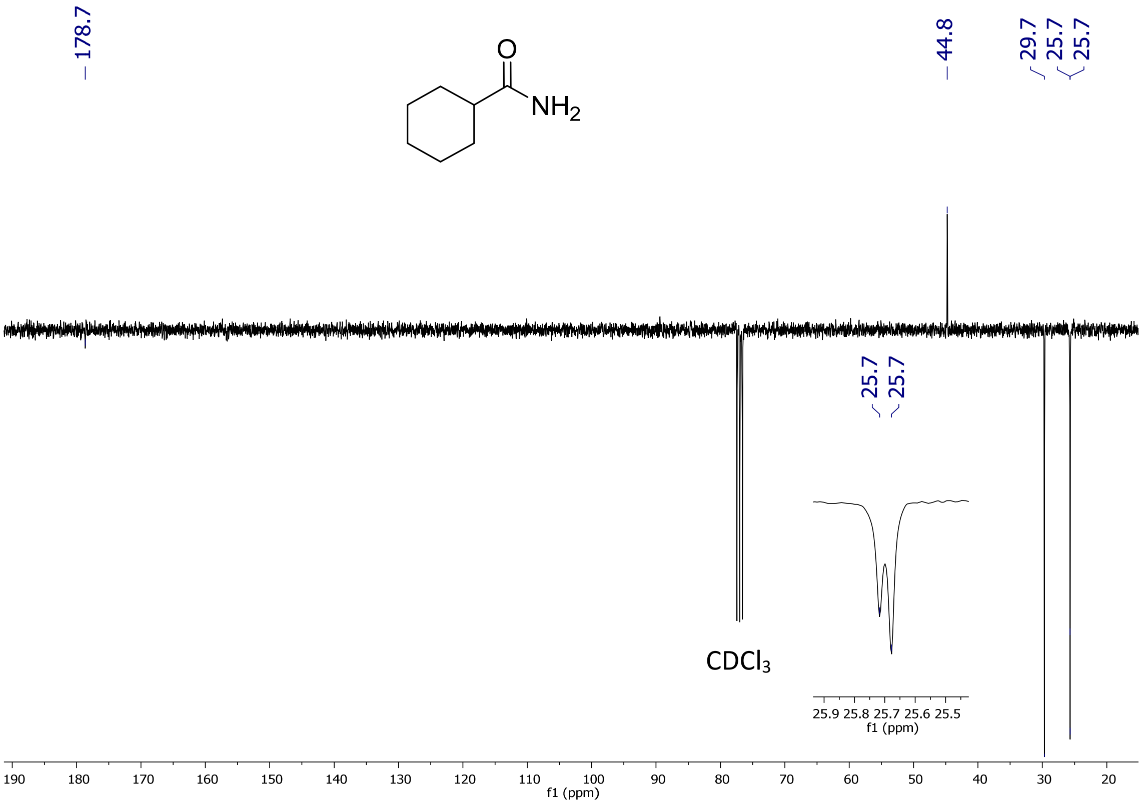

Figure S19. $^{13}C\{^1H\}$ APT NMR (75.48 MHz, $CDCl_3$, 298 K) spectrum of cyclohexanecarbamide.

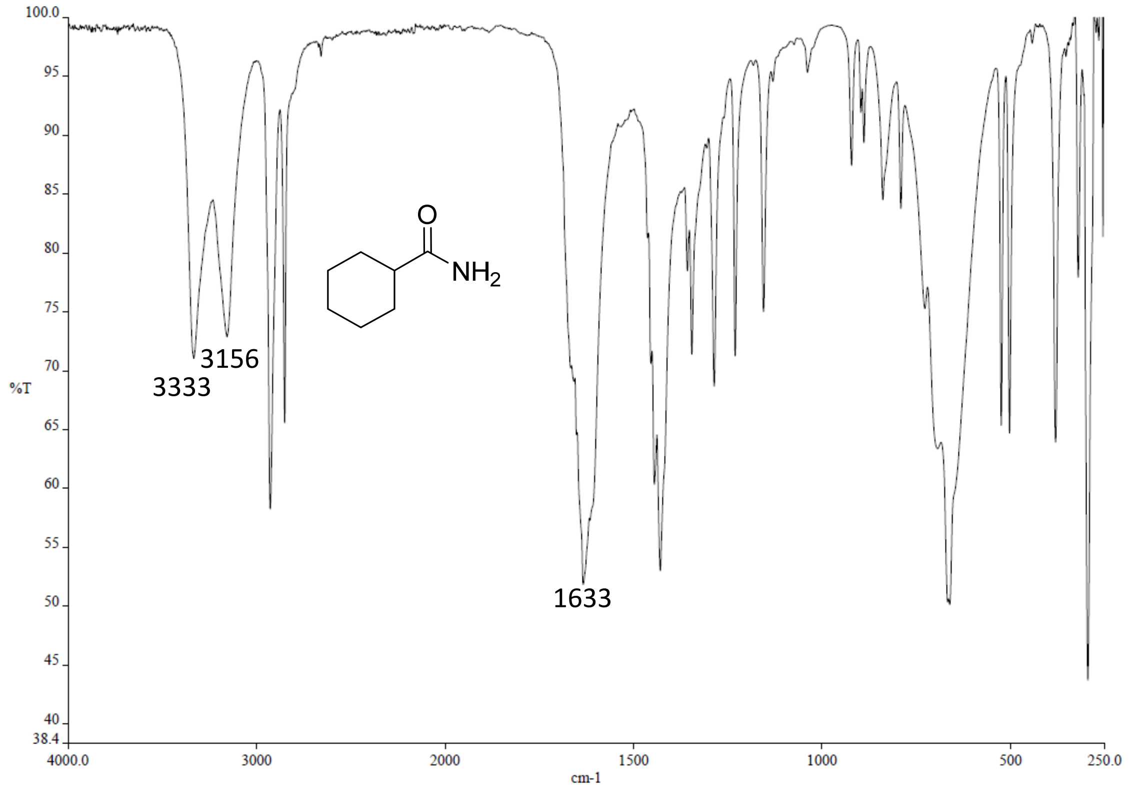

Figure S20. IR ATR spectrum of cyclohexanecarbamide.

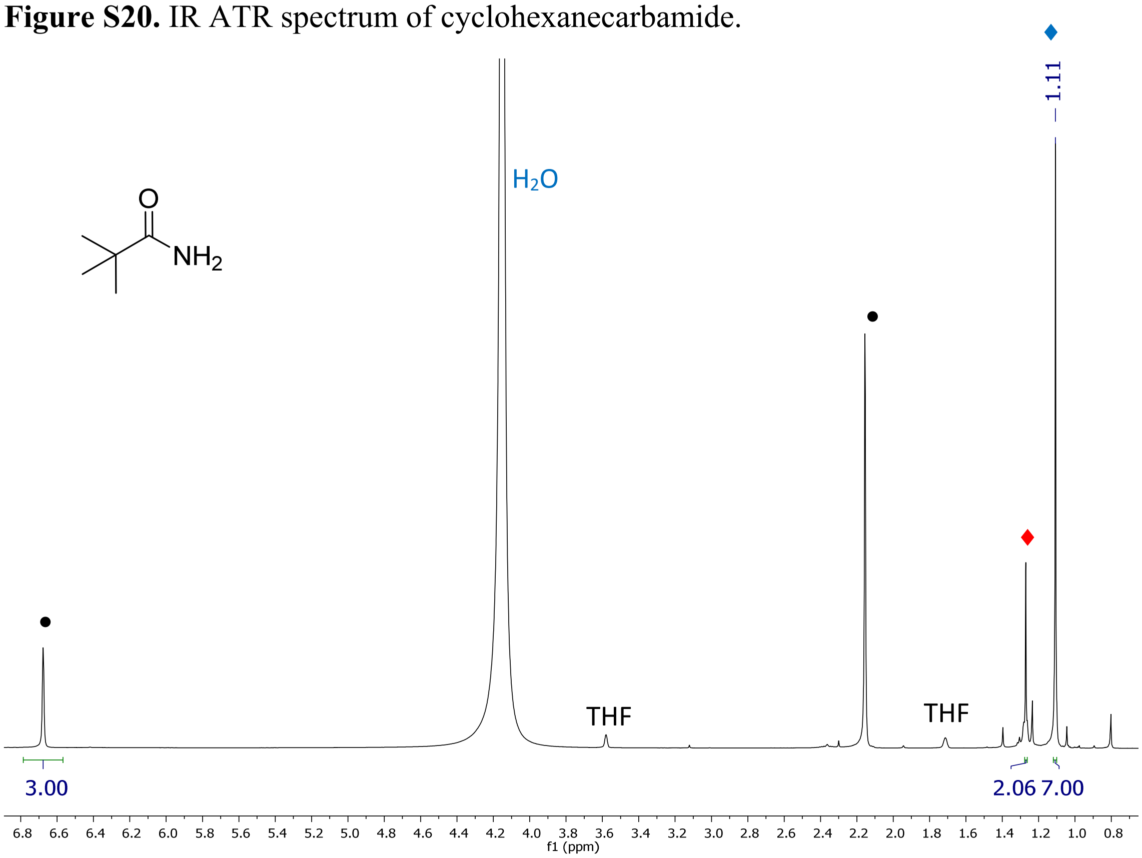

Figure S21. ^{1}H NMR (300.13 MHz, THF-d_8, 298 K) spectrum of the reaction mixture of the hydration of pivalonitrile (♦): formation of pivalamide (♦). • Mesitylene.

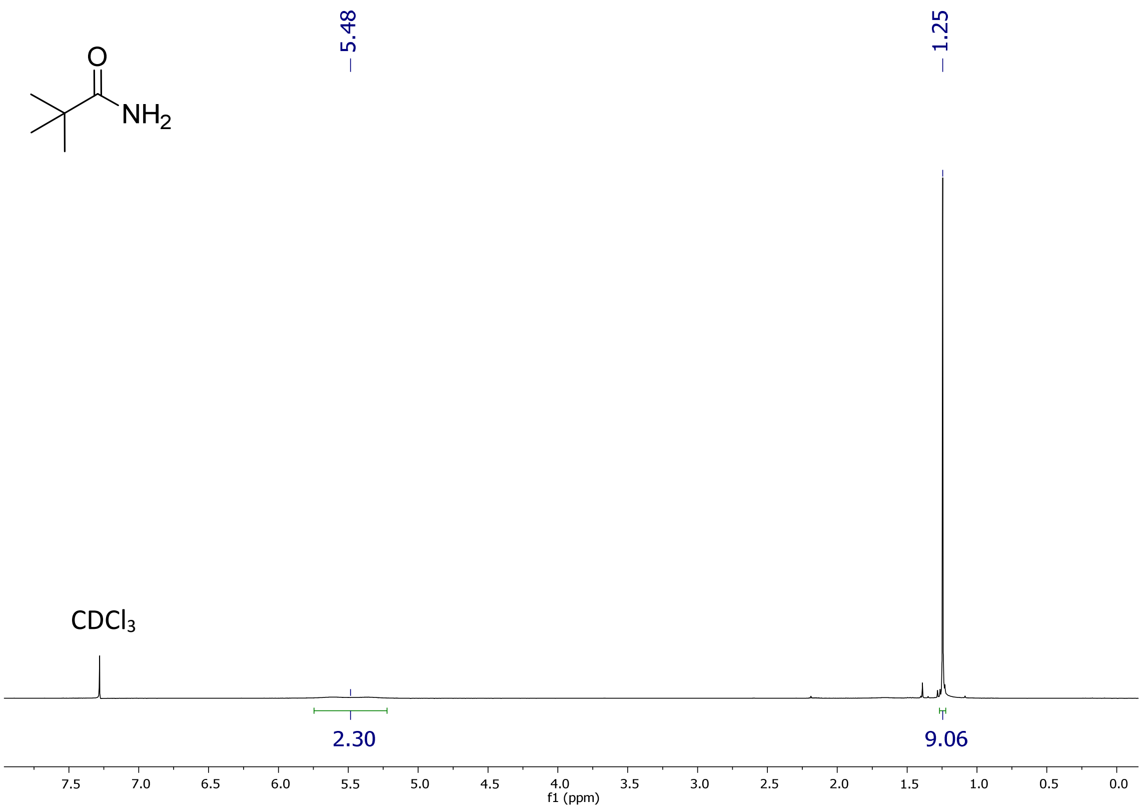

Figure S22. ^{1}H NMR (400.16 MHz, $CDCl_3$, 298 K) spectrum of pivalamide.

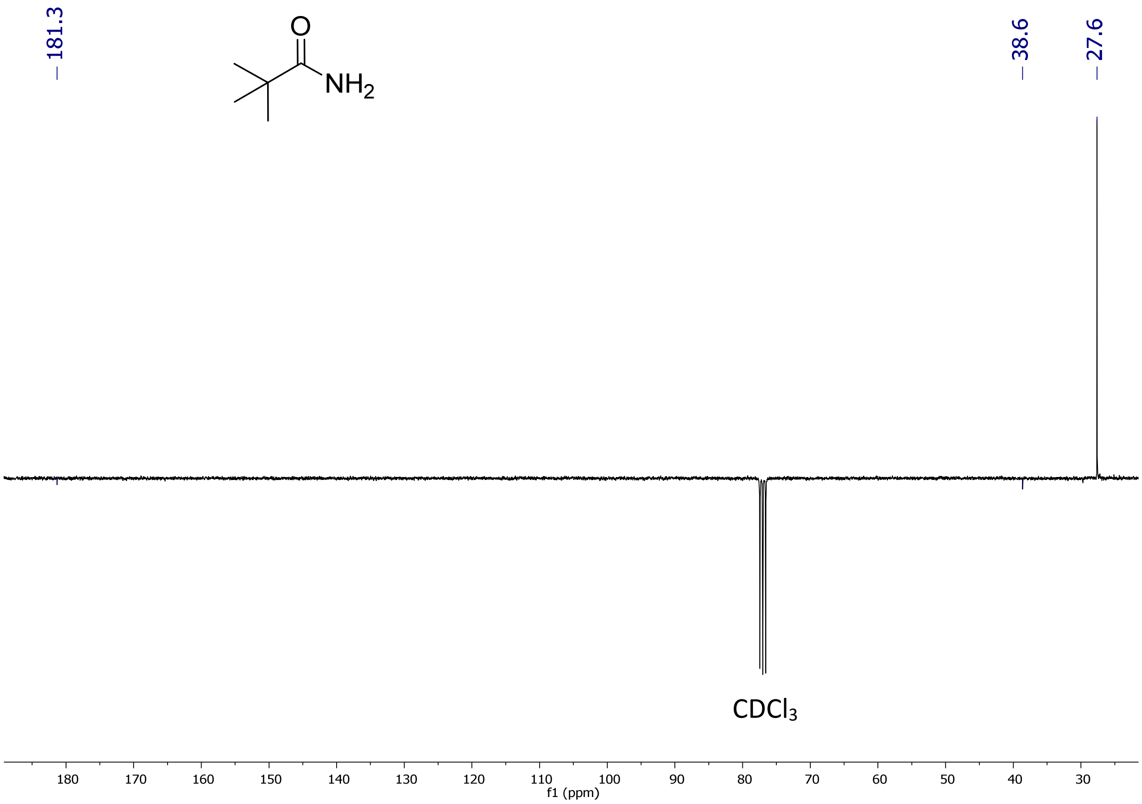

Figure S23. $^{13}C\{^{1}H\}$ APT NMR (75.48 MHz, $CDCl_3$, 298 K) spectrum of pivalamide.

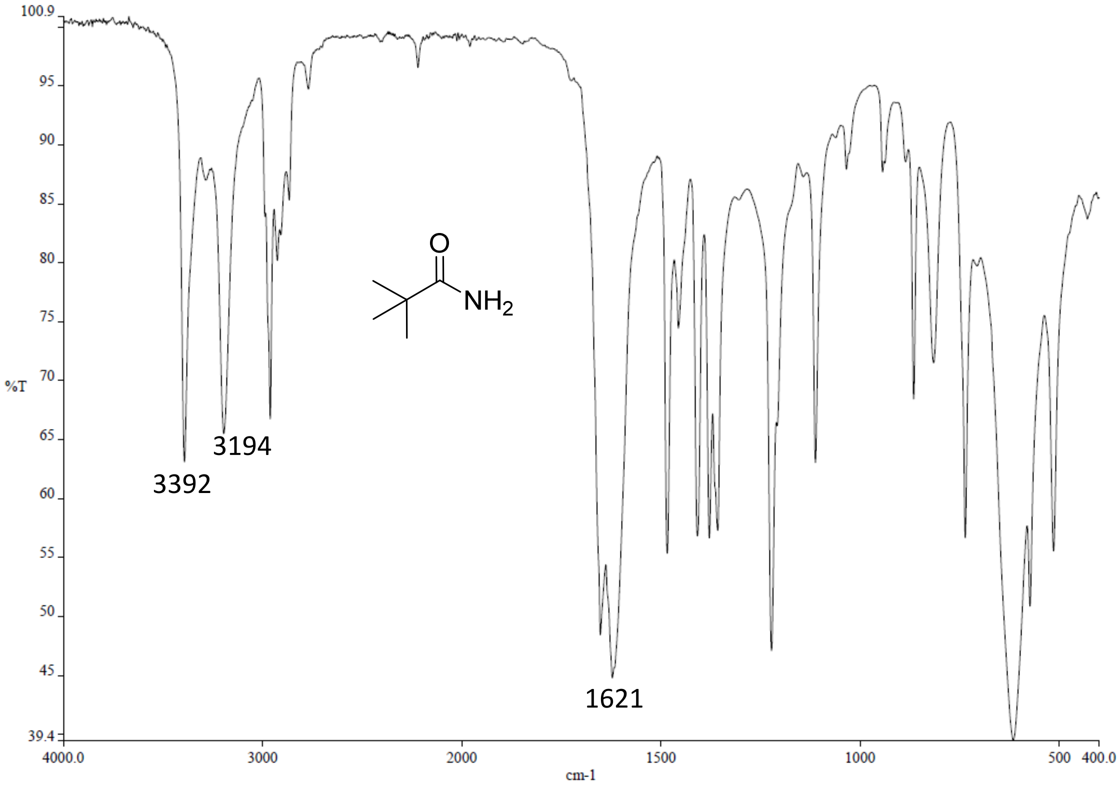

Figure S24. IR ATR spectrum of pivalamide.

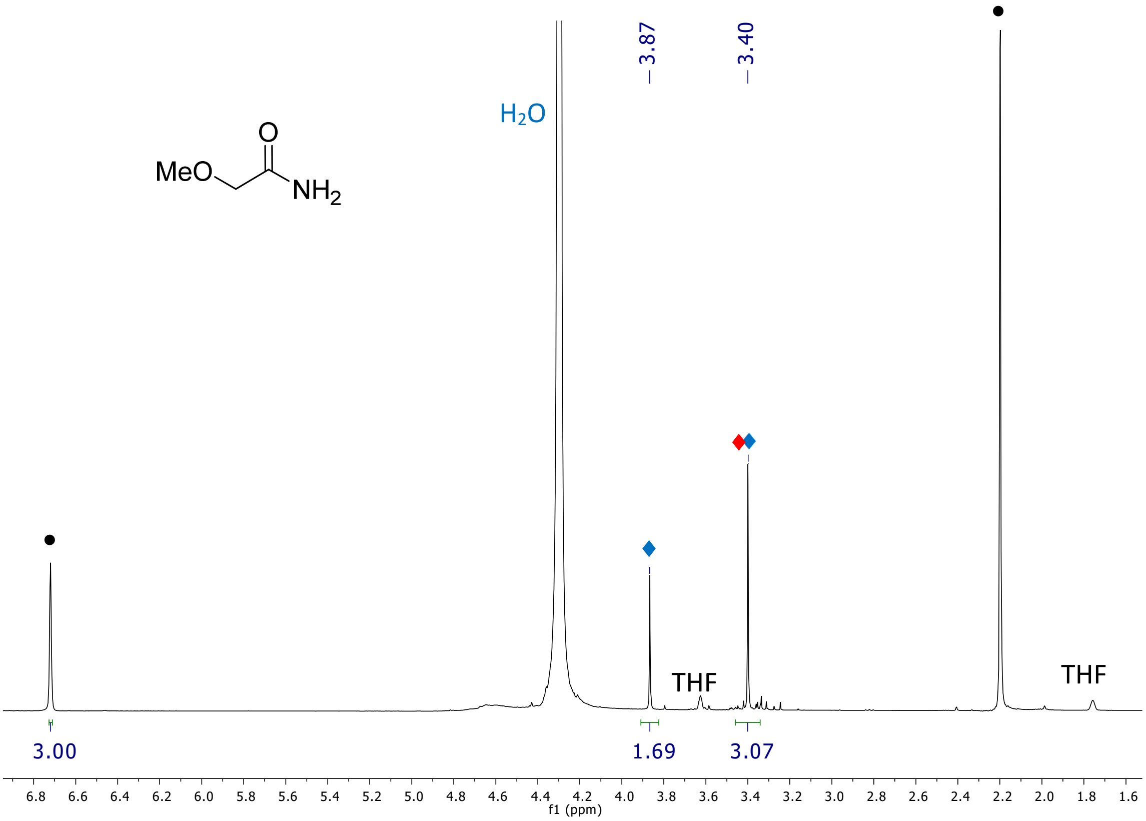

Figure S25. ^{1}H NMR (300.13 MHz, THF-d_8, 298 K) spectrum of the reaction mixture of the hydration of 2-methoxyacetonitrile (♦): formation of 2-methoxyacetamide (♦). • Mesitylene.

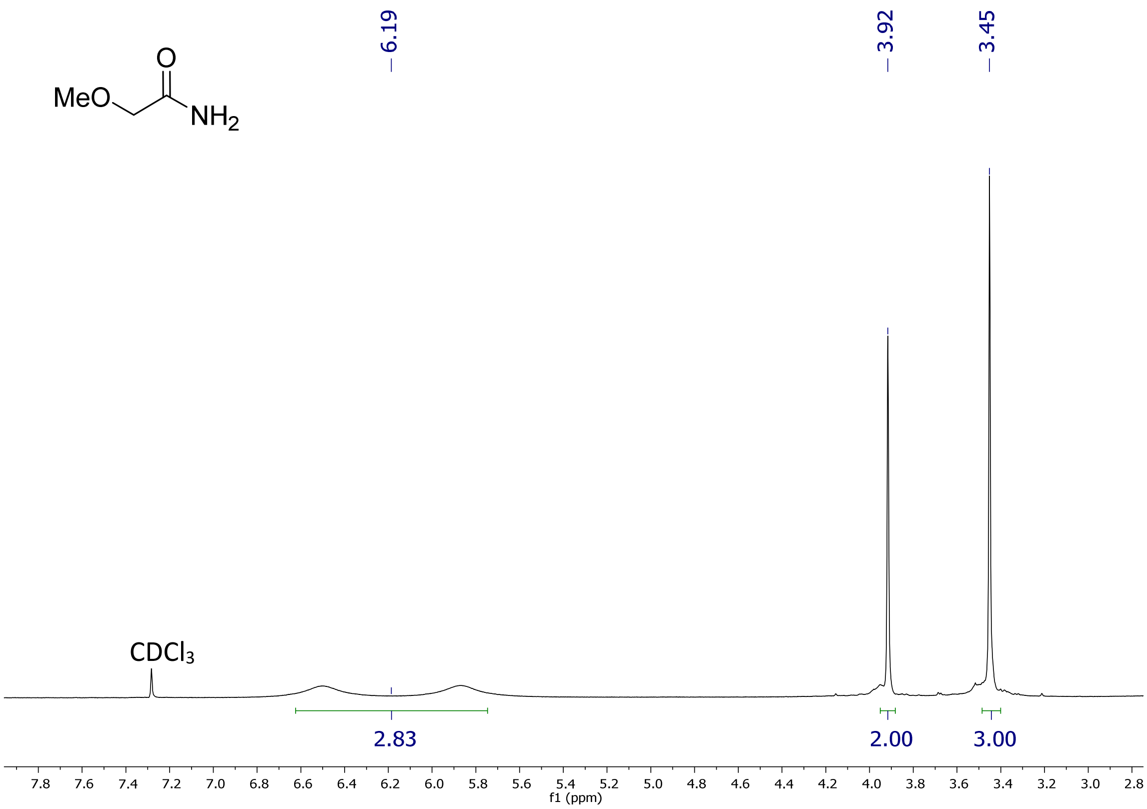

Figure S26. ^{1}H NMR (300.13 MHz, $CDCl_3$, 298 K) spectrum of methoxyacetamide.

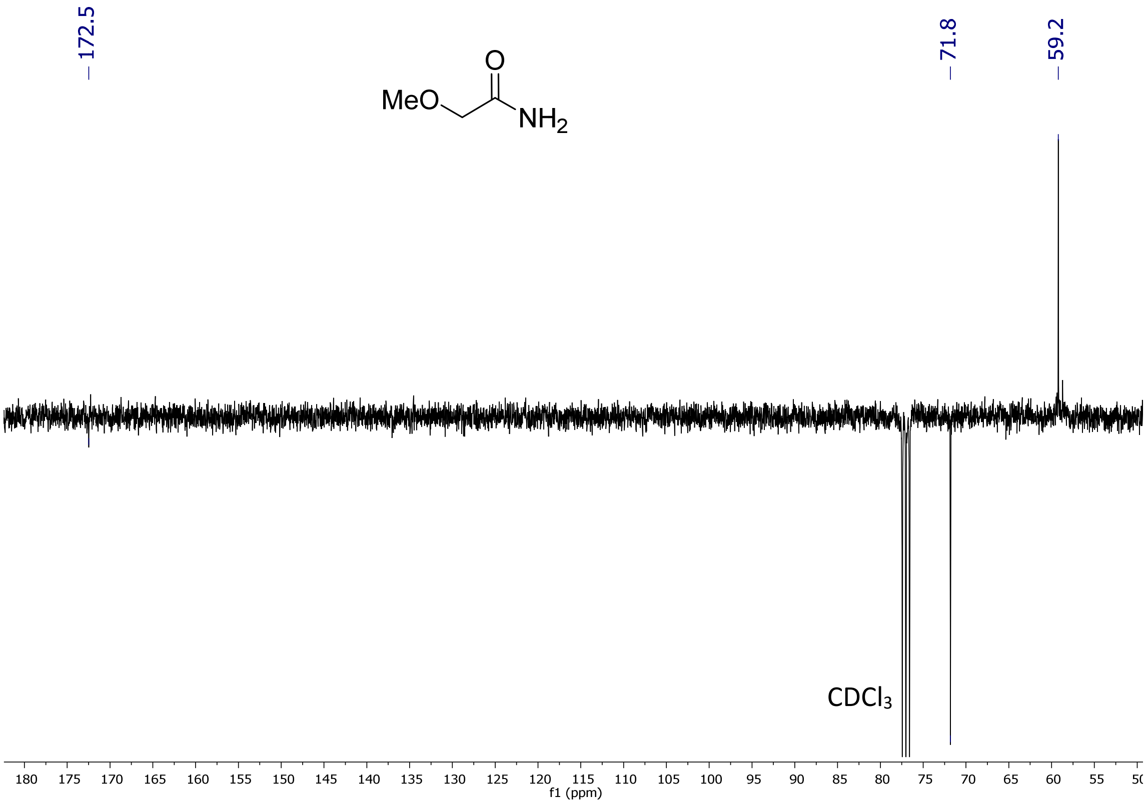

Figure S27. $^{13}C\{^{1}H\}$ APT NMR (75.48 MHz, $CDCl_3$, 298 K) spectrum of methoxyacetamide.

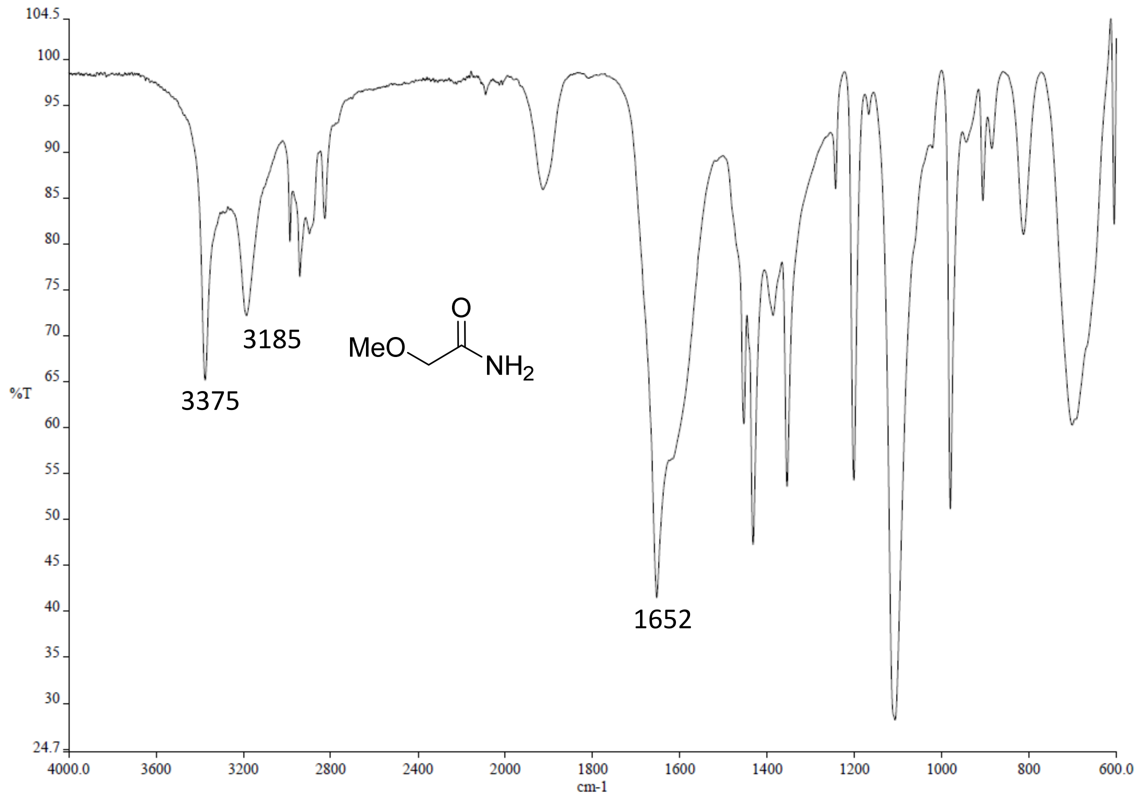

Figure S28. IR ATR spectrum of methoxyacetamide.

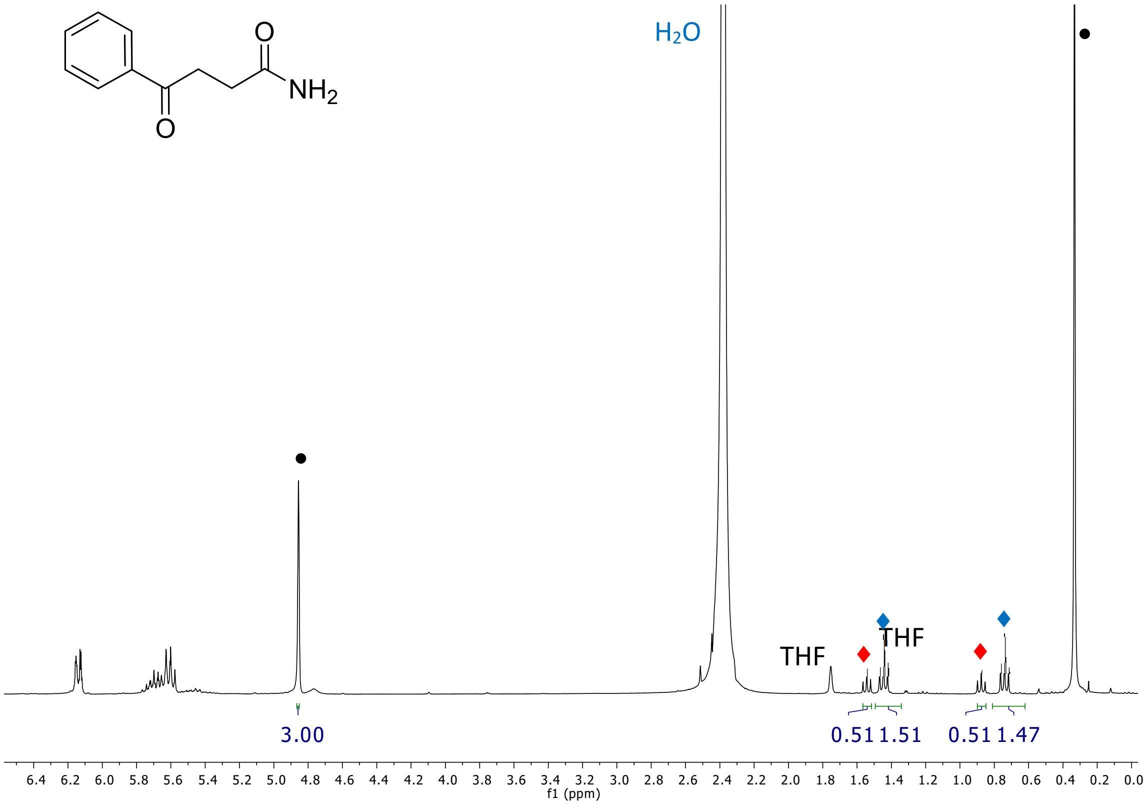

Figure S29. ^{1}H NMR (300.13 MHz, THF-d_8, 298 K) spectrum of the reaction mixture of the hydration of 4-oxo-4-phenylbutanonitrile (♦): formation of 4-oxo-4-phenylbutanamide (♦). • Mesitylene.

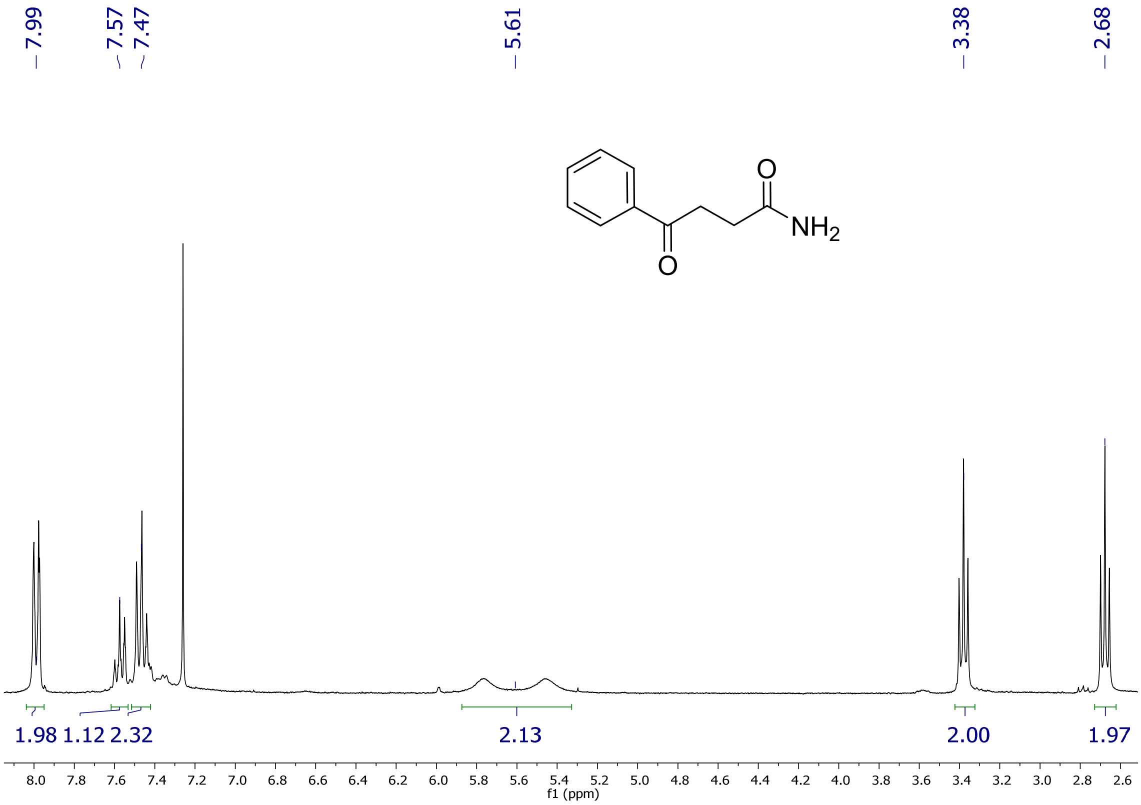

Figure S30. ^{1}H NMR (300.13 MHz, $CDCl_3$, 298 K) spectrum of 4-oxo-4-phenylbutanamide.

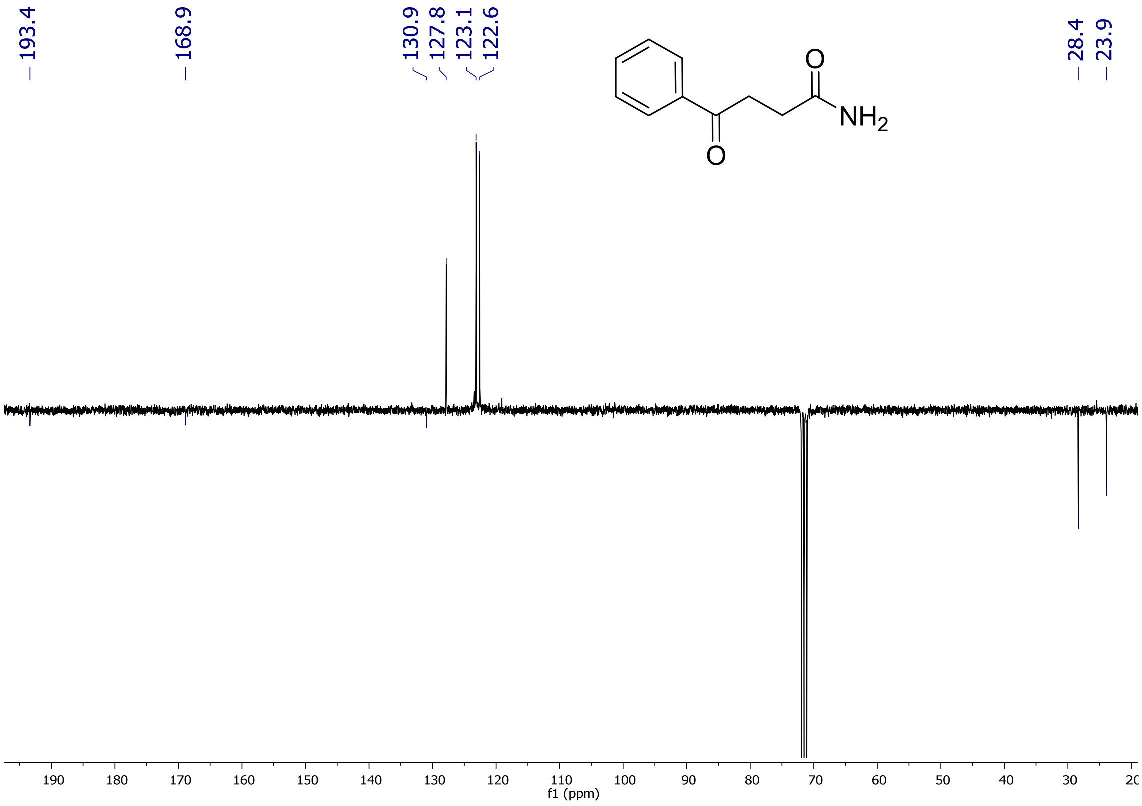

Figure S31. $^{13}C\{^{1}H\}$ APT NMR (75.48 MHz, $CDCl_3$, 298 K) spectrum of 4-oxo-4-phenylbutanamide.

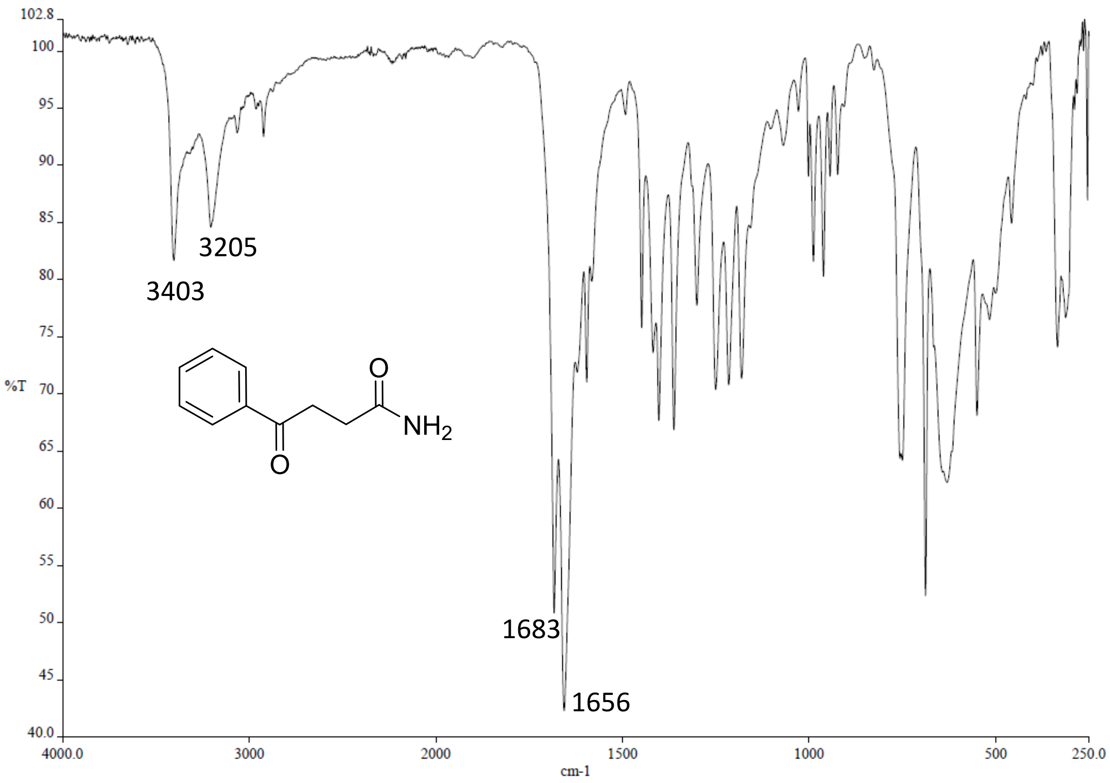

Figure S32. IR ATR spectrum of 4-oxo-4-phenylbutanamide.

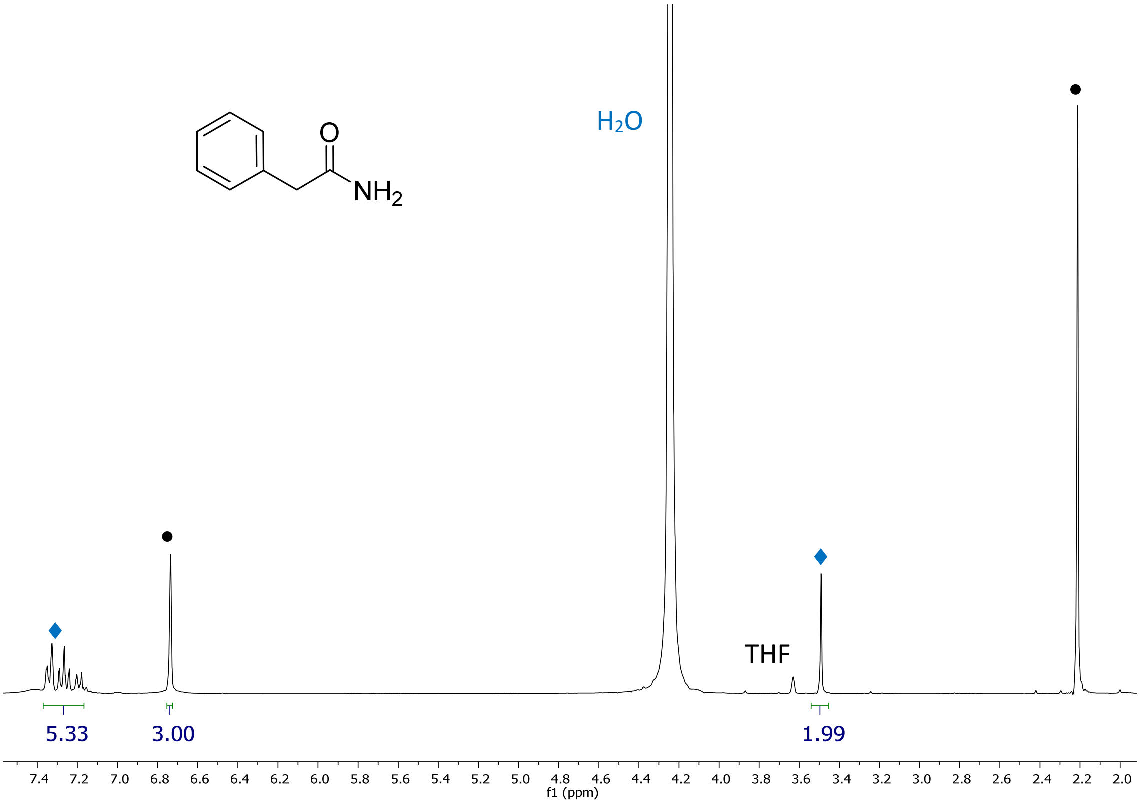

Figure S33. ^{1}H NMR (300.13 MHz, THF-d_8, 298 K) spectrum of the reaction mixture of the hydration of 2-phenylacetonitrile (♦): formation of 2-phenylacetamide (♦). • Mesitylene.

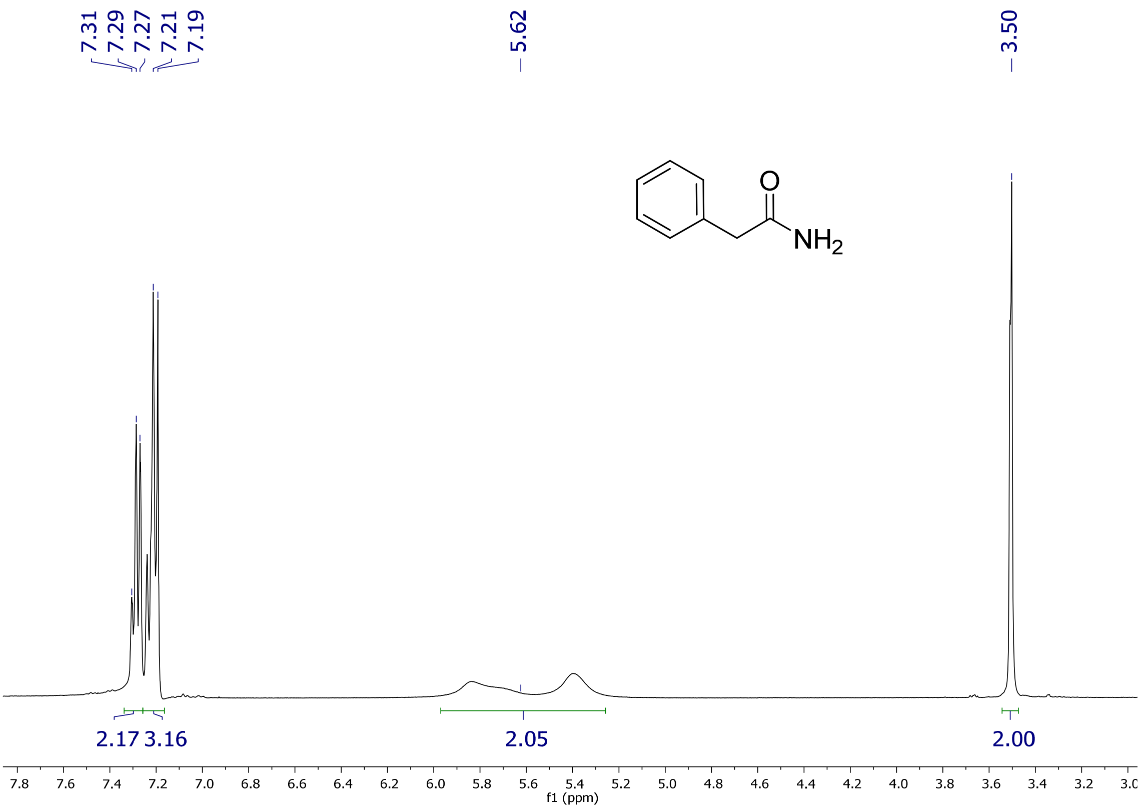

Figure S34. ^{1}H NMR (400.16 MHz, $CDCl_3$, 298 K) spectrum of 2-phenylacetamide.

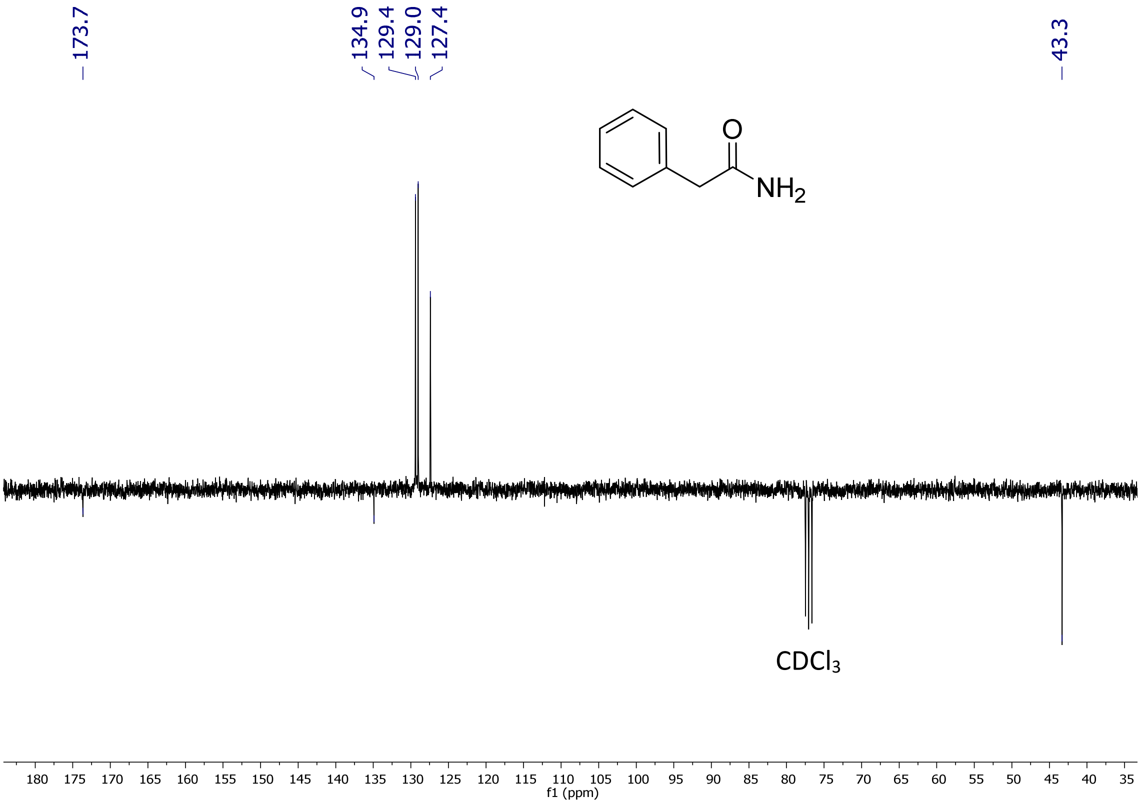

Figure S35. $^{13}C\{^{1}H\}$ APT NMR (75.48 MHz, $CDCl_3$, 298 K) spectrum of 2-phenylacetamide.

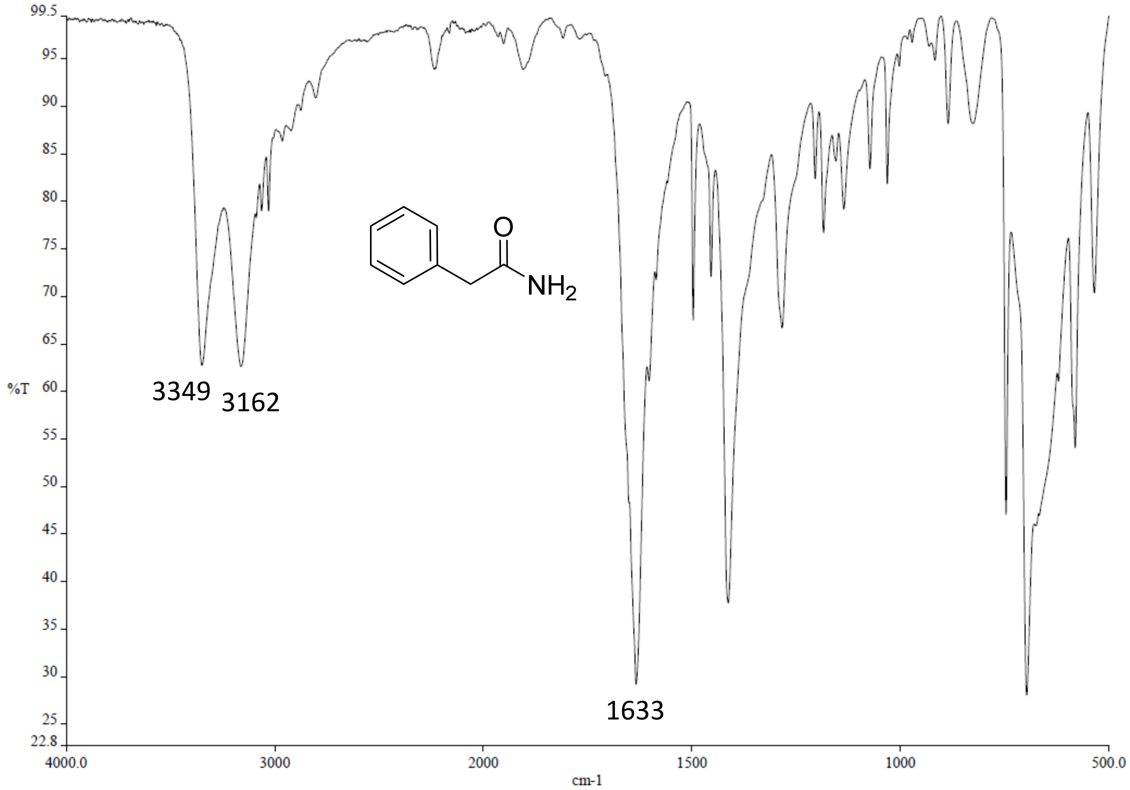

Figure S36. IR ATR spectrum of 2-phenylacetamide.

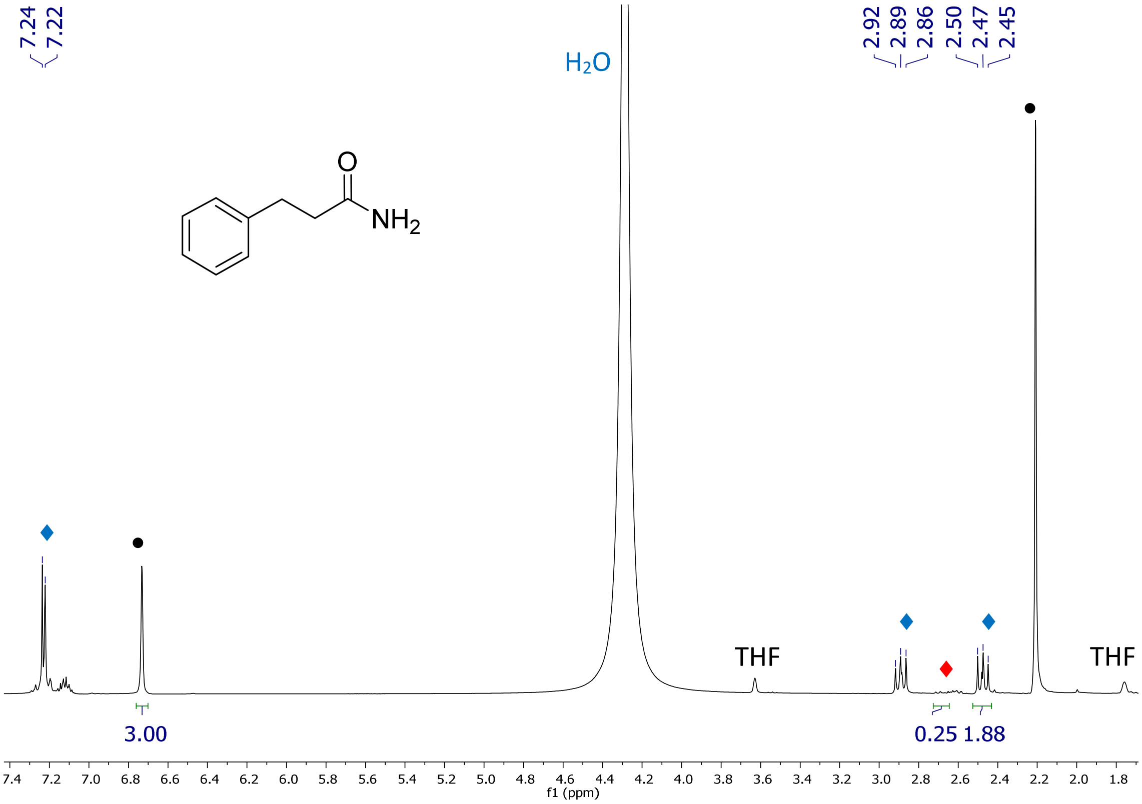

Figure S37. ^{1}H NMR (300.13 MHz, THF-d_8, 298 K) spectrum of the reaction mixture of the hydration of 3-phenylpropanenitrile (♦): formation of 3-phenylpropanamide (♦). • Mesitylene.

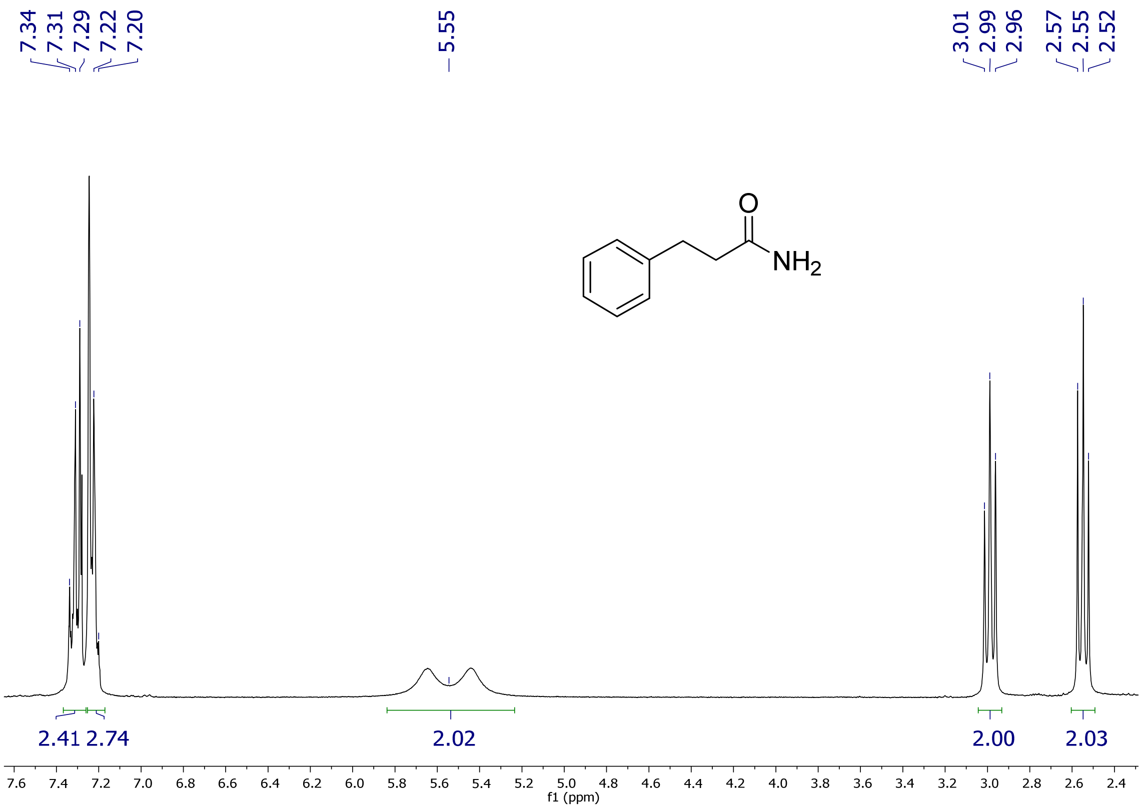

Figure S38. ^{1}H NMR (300.13 MHz, $CDCl_3$, 298 K) spectrum of 3-phenylpropanamide.

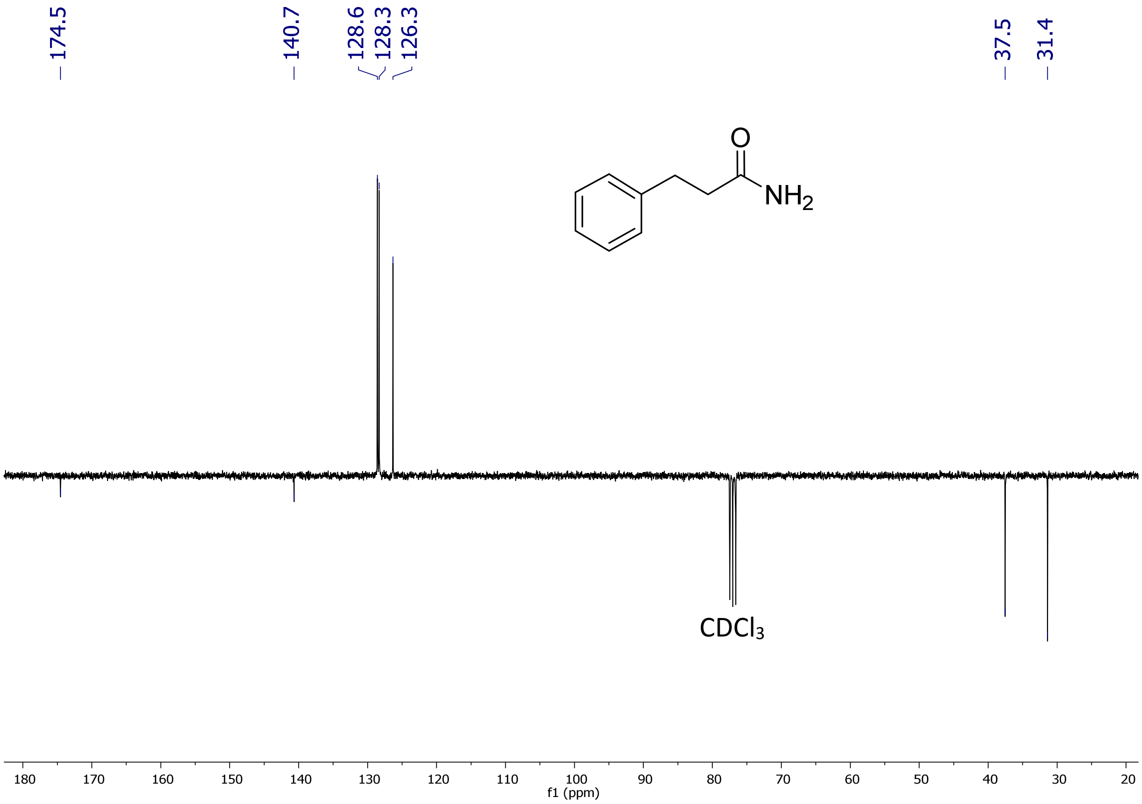

Figure S39. $^{13}C\{^{1}H\}$ APT NMR (75.48 MHz, $CDCl_3$, 298 K) spectrum of 3-phenylpropanamide.

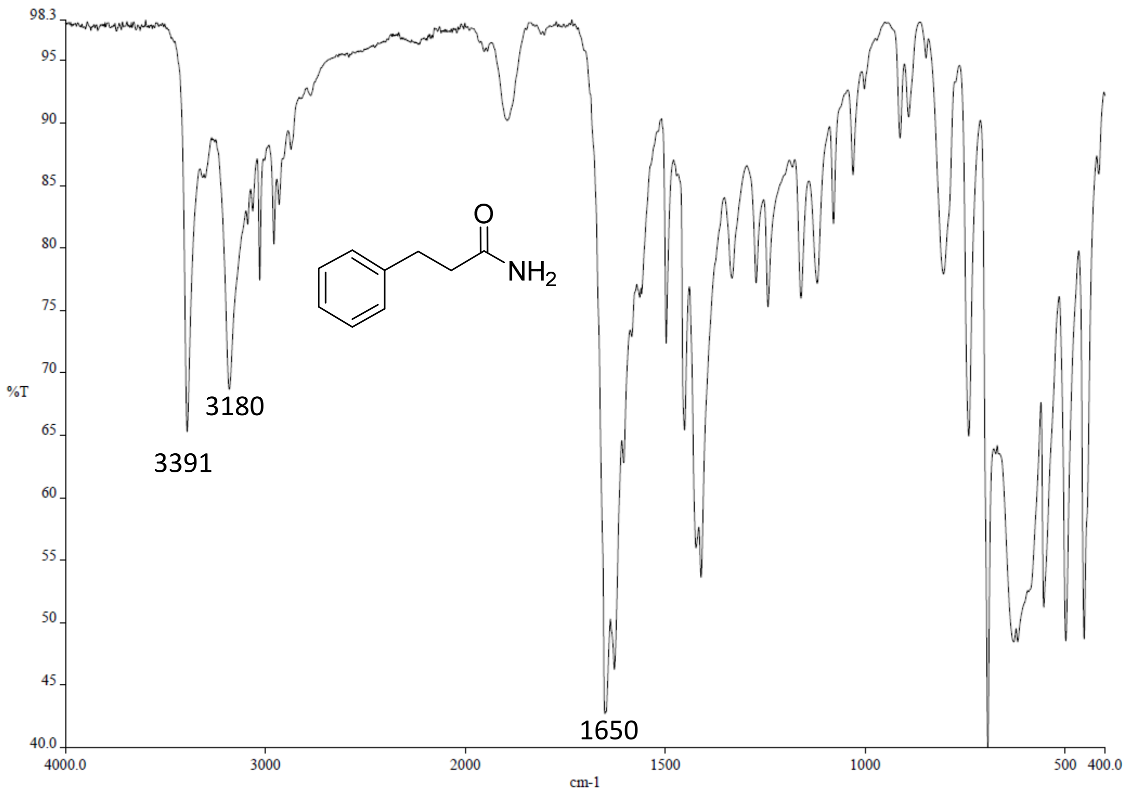

Figure S40. IR ATR spectrum of 3-phenylpropanamide.

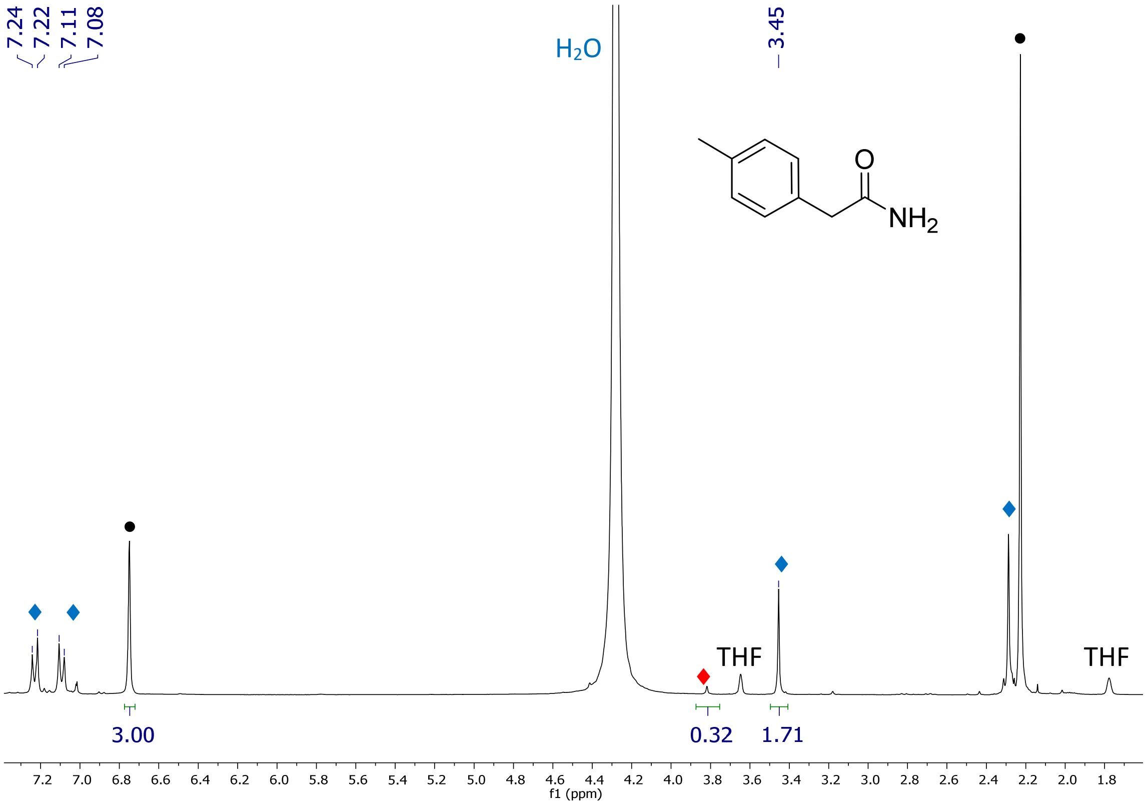

Figure S41. ^{1}H NMR (300.13 MHz, THF-d_8, 298 K) spectrum of the reaction mixture of the hydration of *p*-tolylacetonitrile (♦): formation of *p*-tolylacetamide (♦). • Mesitylene.

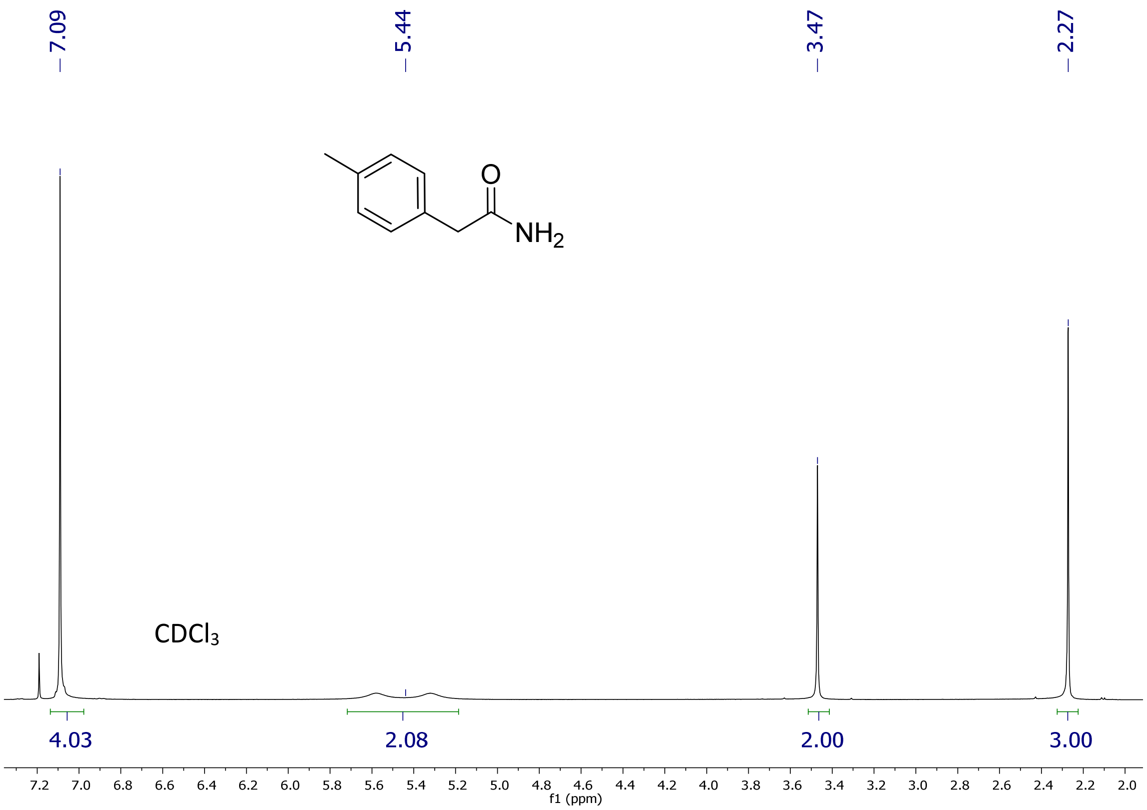

Figure S42. ^{1}H NMR (400.16 MHz, $CDCl_3$, 298 K) spectrum of *p*-tolylacetamide.

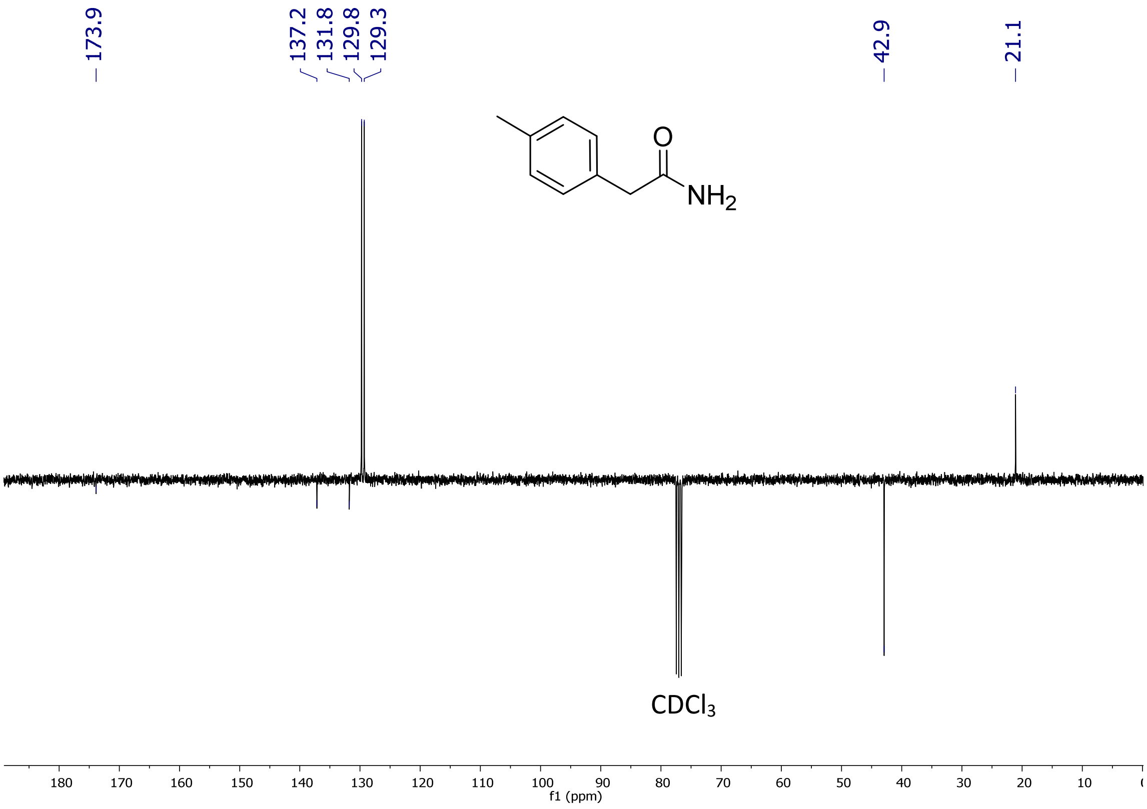

Figure S43. $^{13}C\{^{1}H\}$ APT NMR (75.48 MHz, $CDCl_3$, 298 K) spectrum of *p*-tolylacetamide.

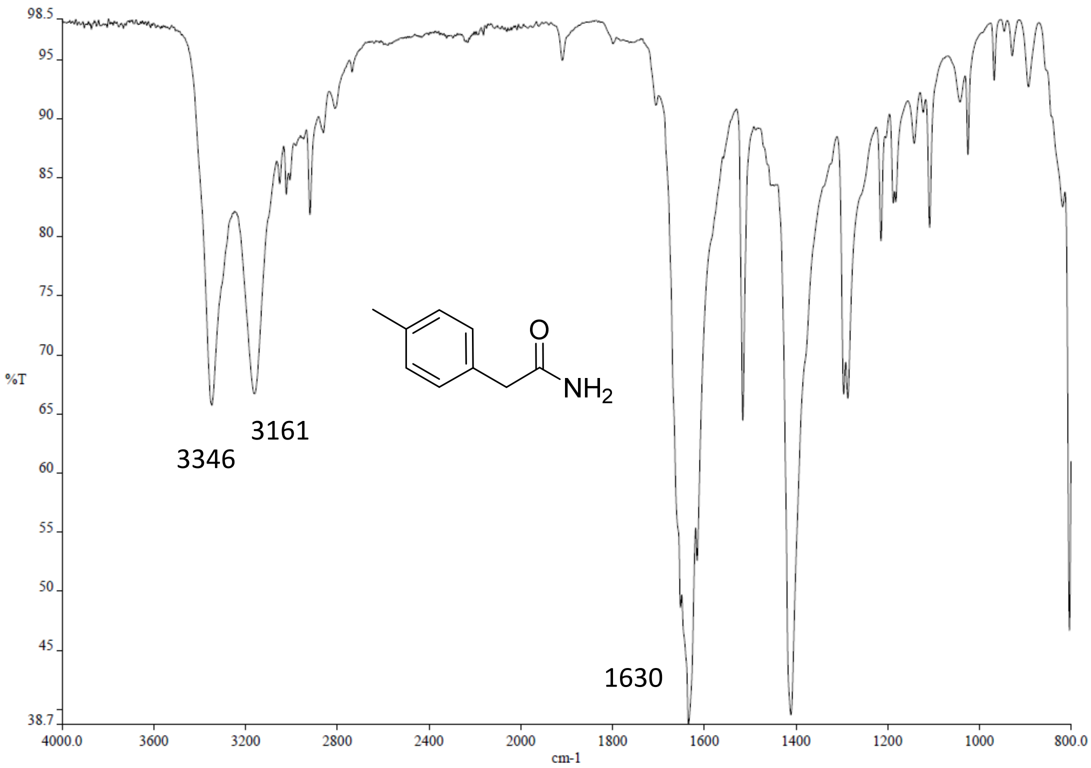

Figure S44. IR ATR spectrum of *p*-tolylacetamide.

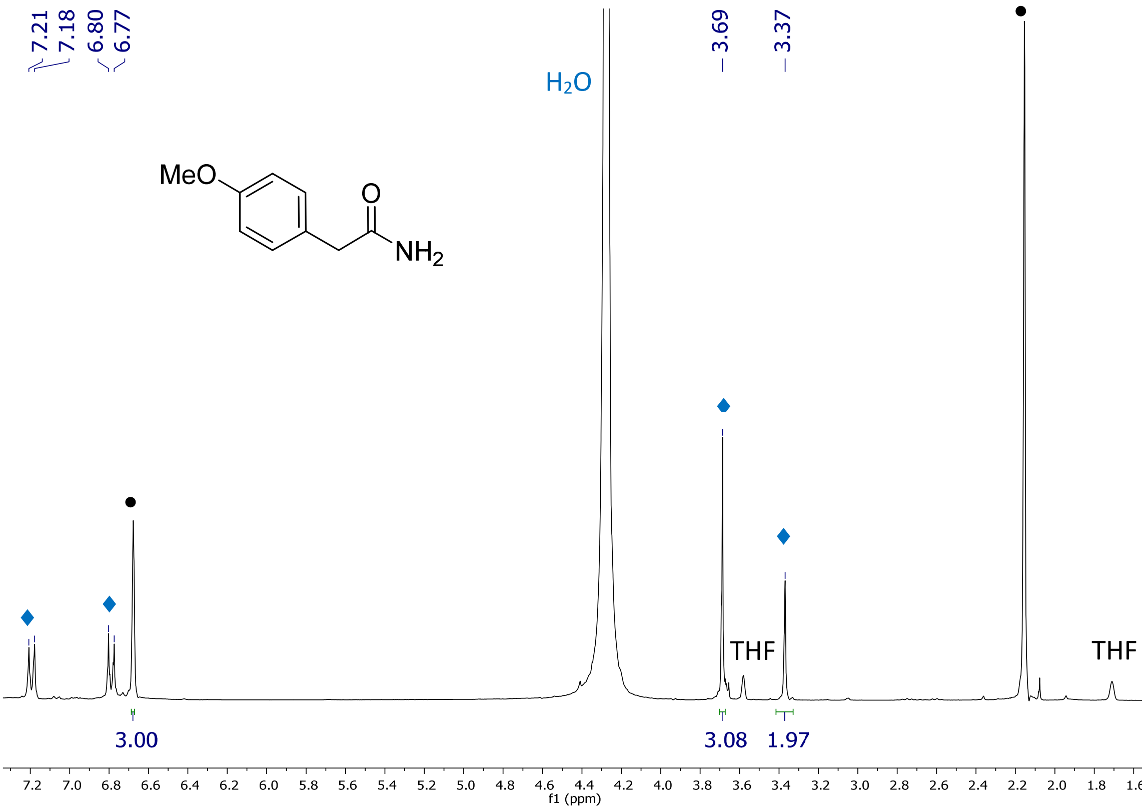

Figure S45. ^{1}H NMR (300.13 MHz, THF-*d*8, 298 K) spectrum of the reaction mixture of the hydration of 4-methoxyphenylacetonitrile (♦): formation of 4-methoxyphenylacetamide (♦). • Mesitylene.

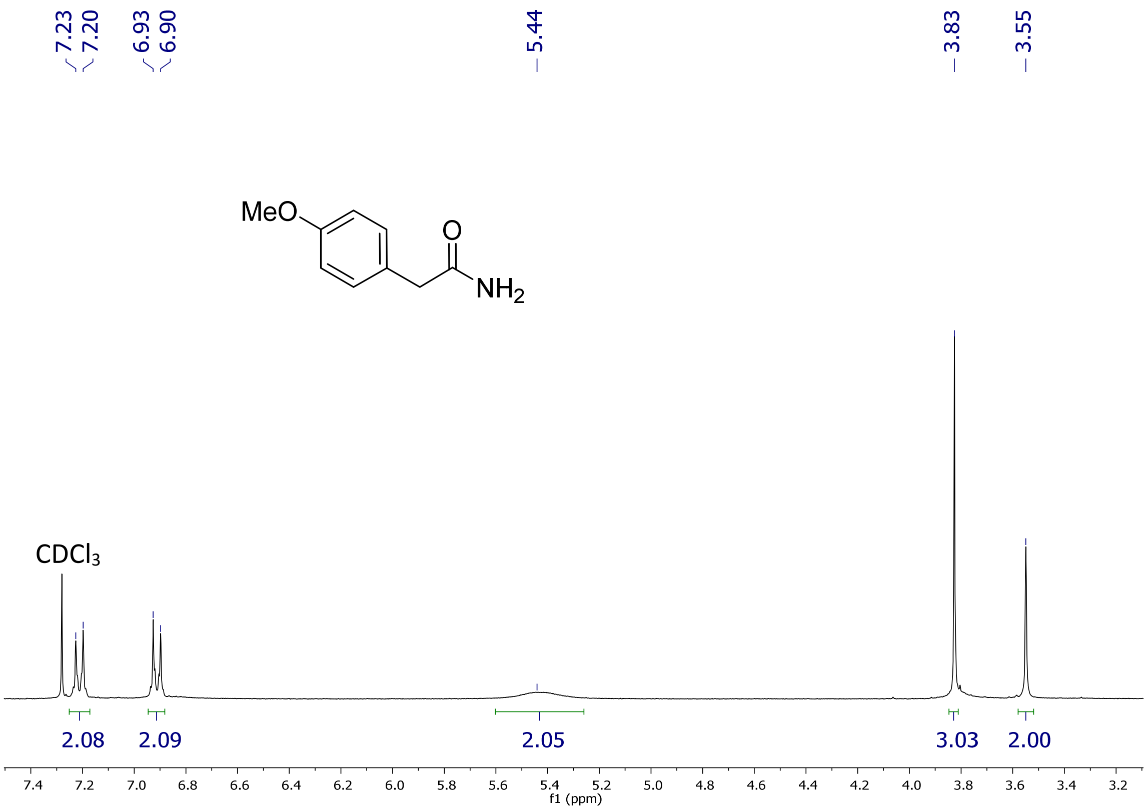

Figure S46. ^{1}H NMR (300.13 MHz, $CDCl_3$, 298 K) spectrum of 4-methoxyphenylacetamide.

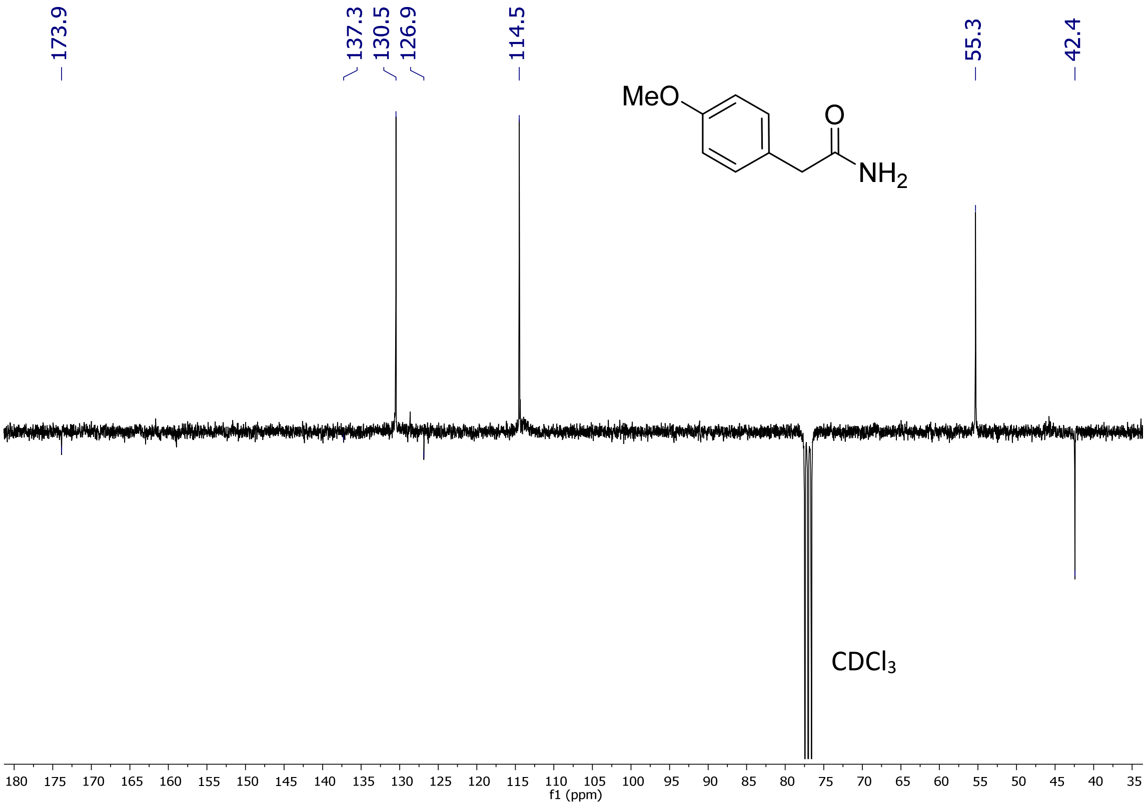

Figure S47. $^{13}C\{^{1}H\}$ APT NMR (75.48 MHz, $CDCl_3$, 298 K) spectrum of 4-methoxyphenylacetamide.

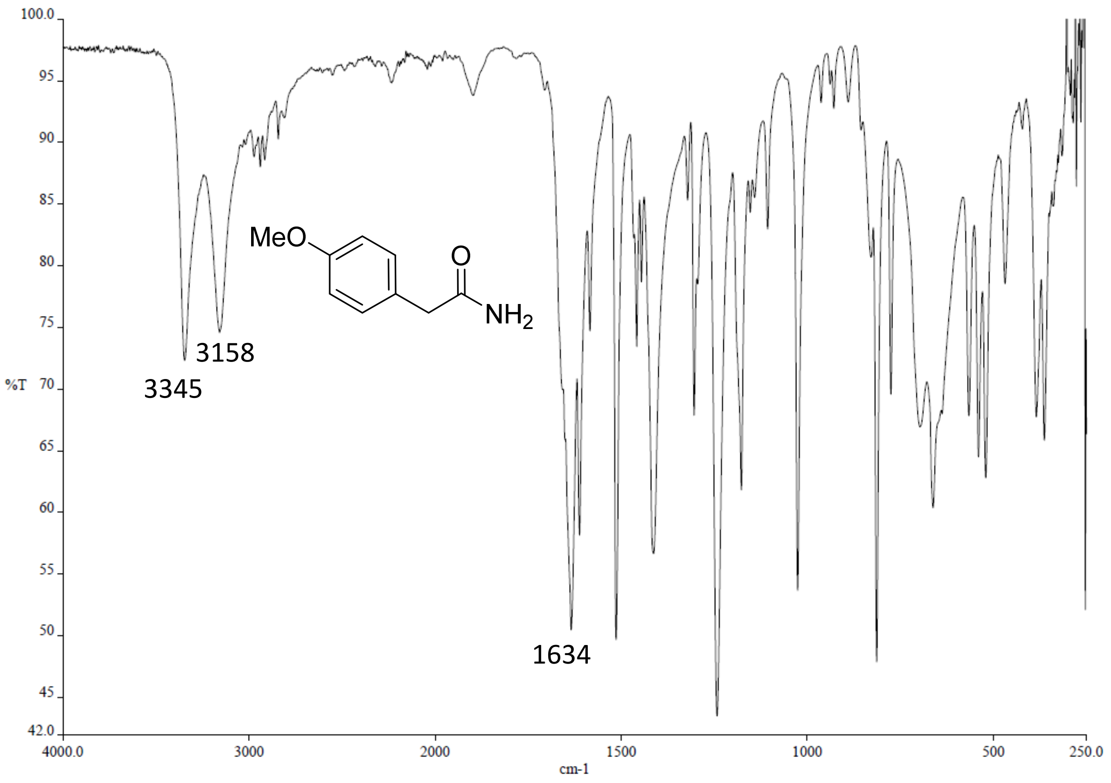

Figure S48. IR ATR spectrum of 4-methoxyphenylacetamide.

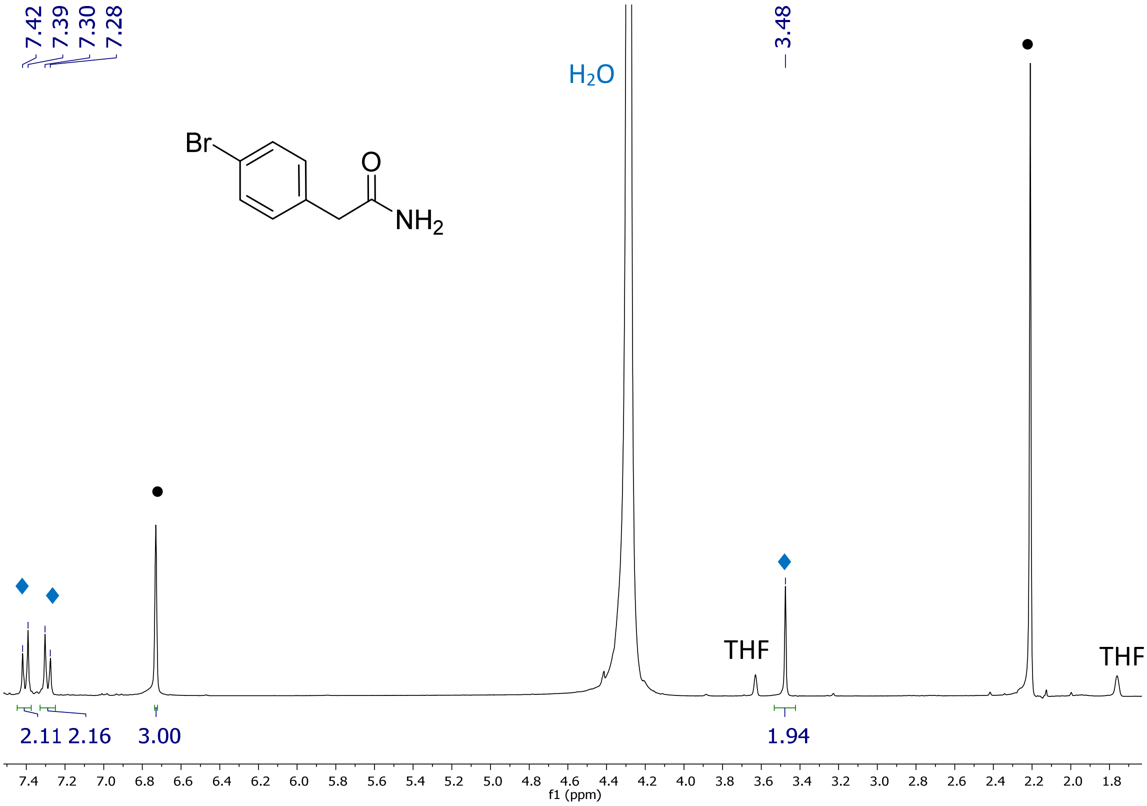

Figure S49. ^{1}H NMR (300.13 MHz, THF-d_8, 298 K) spectrum of the reaction mixture of the hydration of 4-bromoyphenylacetonitrile (♦): formation of 4-bromophenylacetamide (♦). • Mesitylene.

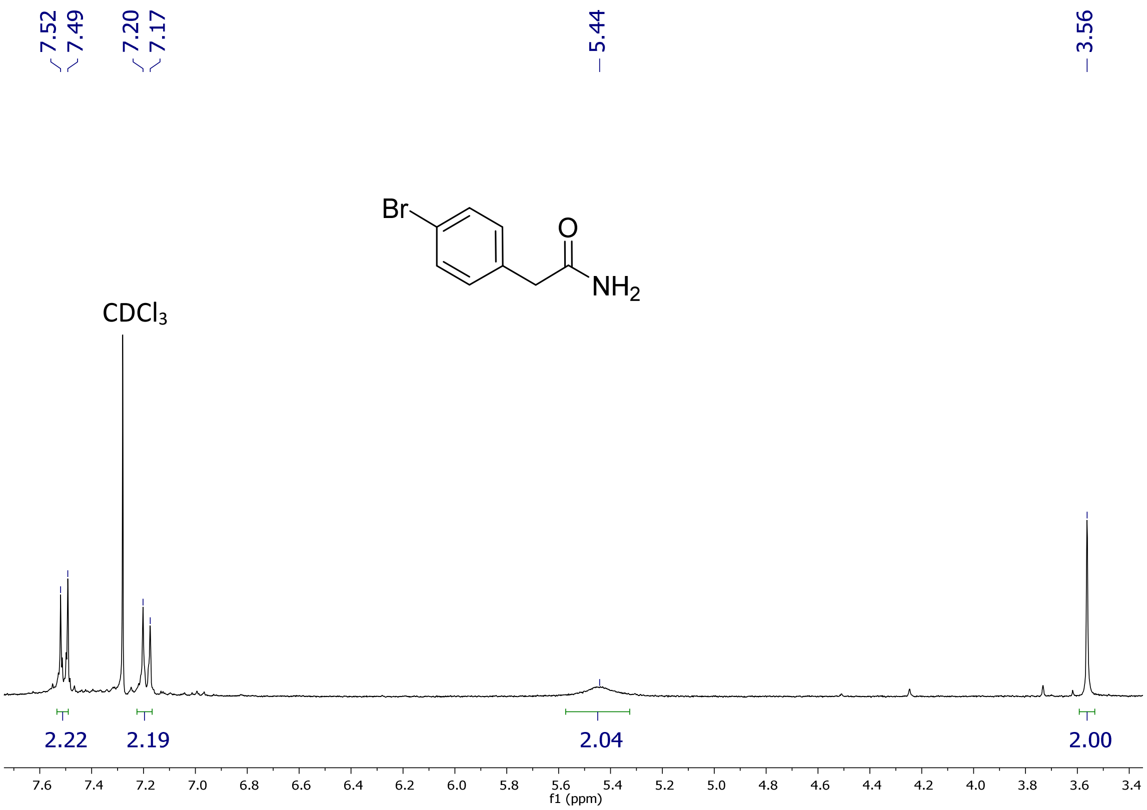

Figure S50. ^{1}H NMR (300.13 MHz, $CDCl_3$, 298 K) spectrum of 4-bromophenylacetamide.

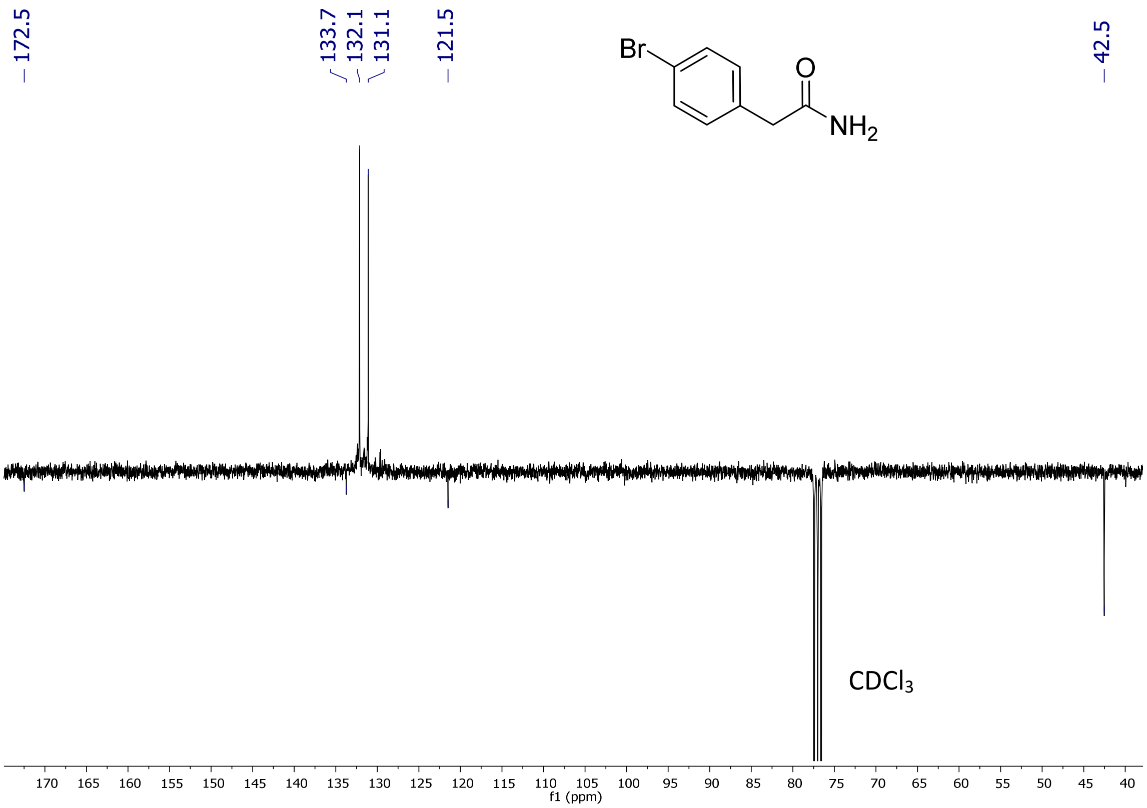

Figure S51. $^{13}C\{^{1}H\}$ APT NMR (75.48 MHz, $CDCl_3$, 298 K) spectrum of 4-bromophenylacetamide.

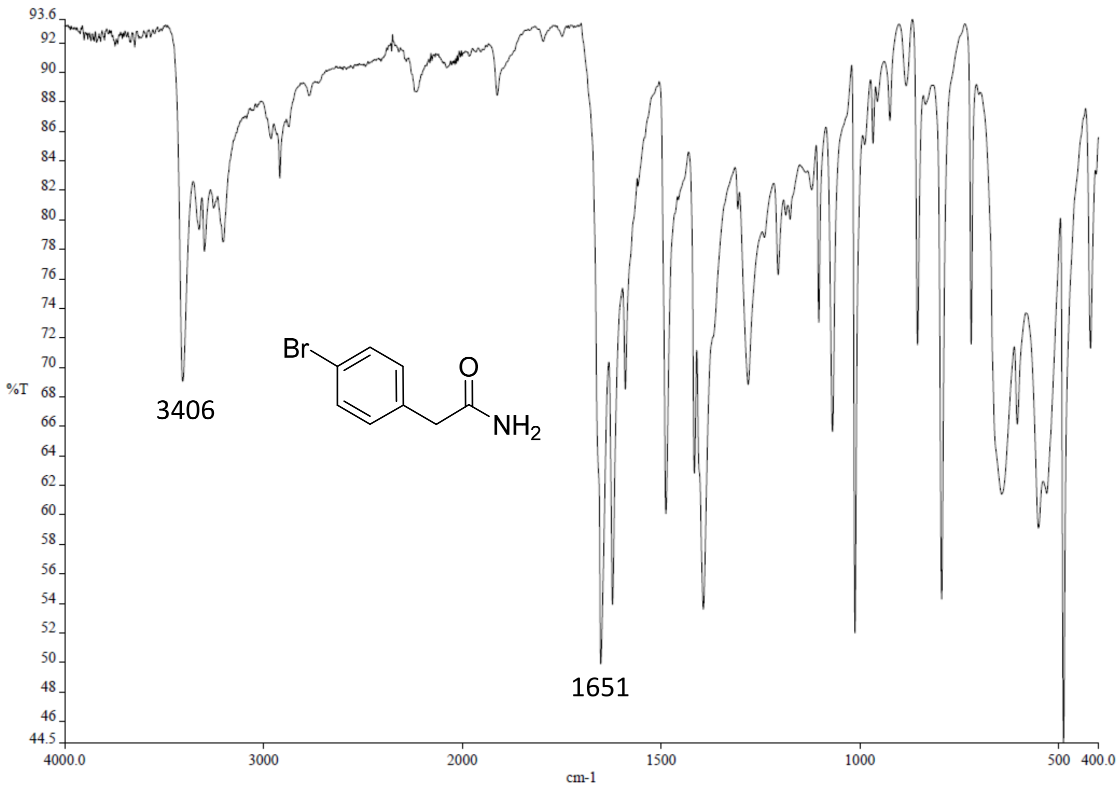

Figure S52. IR ATR spectrum of 4-bromophenylacetamide.

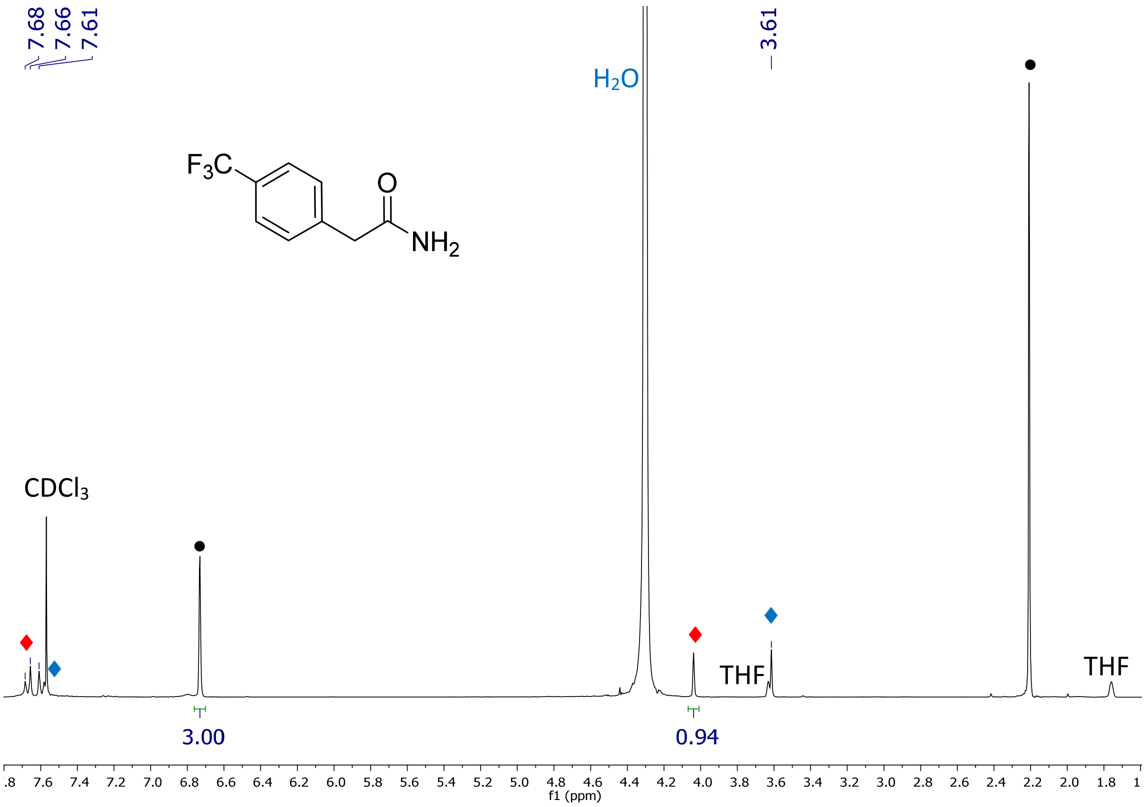

Figure S53. ^{1}H NMR (300.13 MHz, THF-d_8, 298 K) spectrum of the reaction mixture of the hydration of 4-(trifluoromethyl)phenylacetonitrile (♦): formation of 4-(trifluoromethyl)phenylacetamide (♦). • Mesitylene.

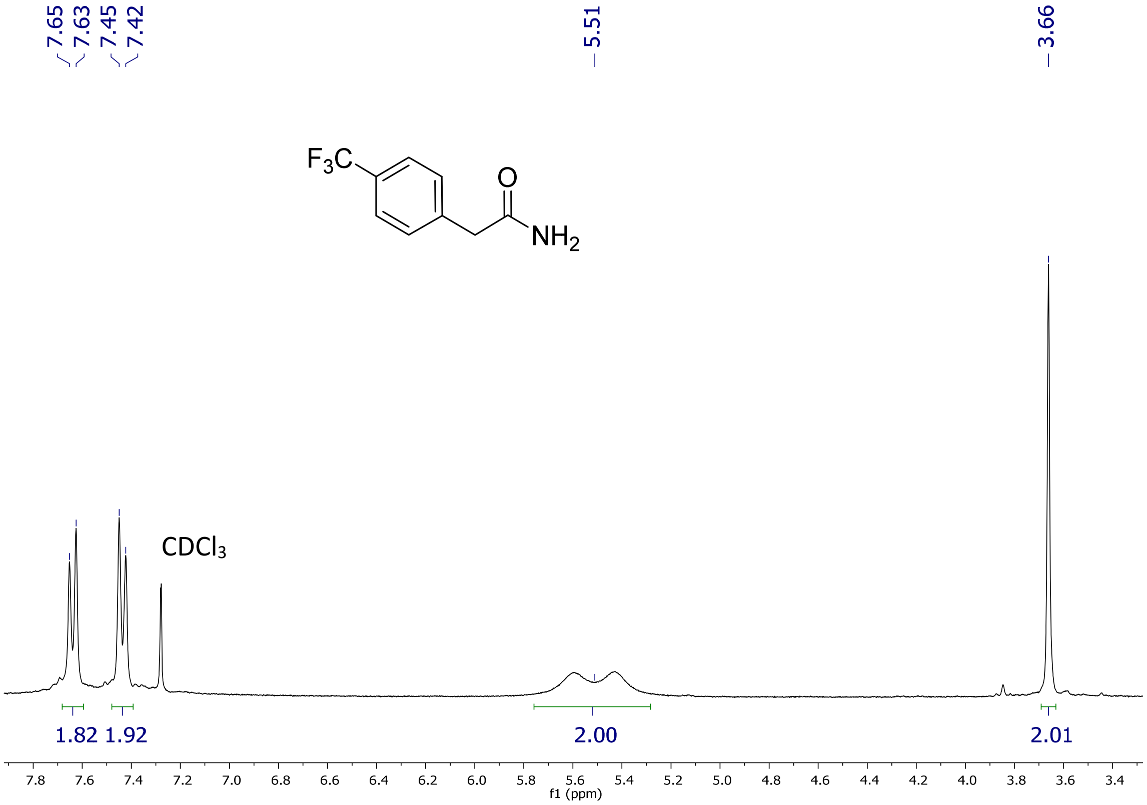

Figure S54. ^{1}H NMR (300.13 MHz, $CDCl_3$, 298 K) spectrum of 4-(trifluoromethyl)phenylacetamide.

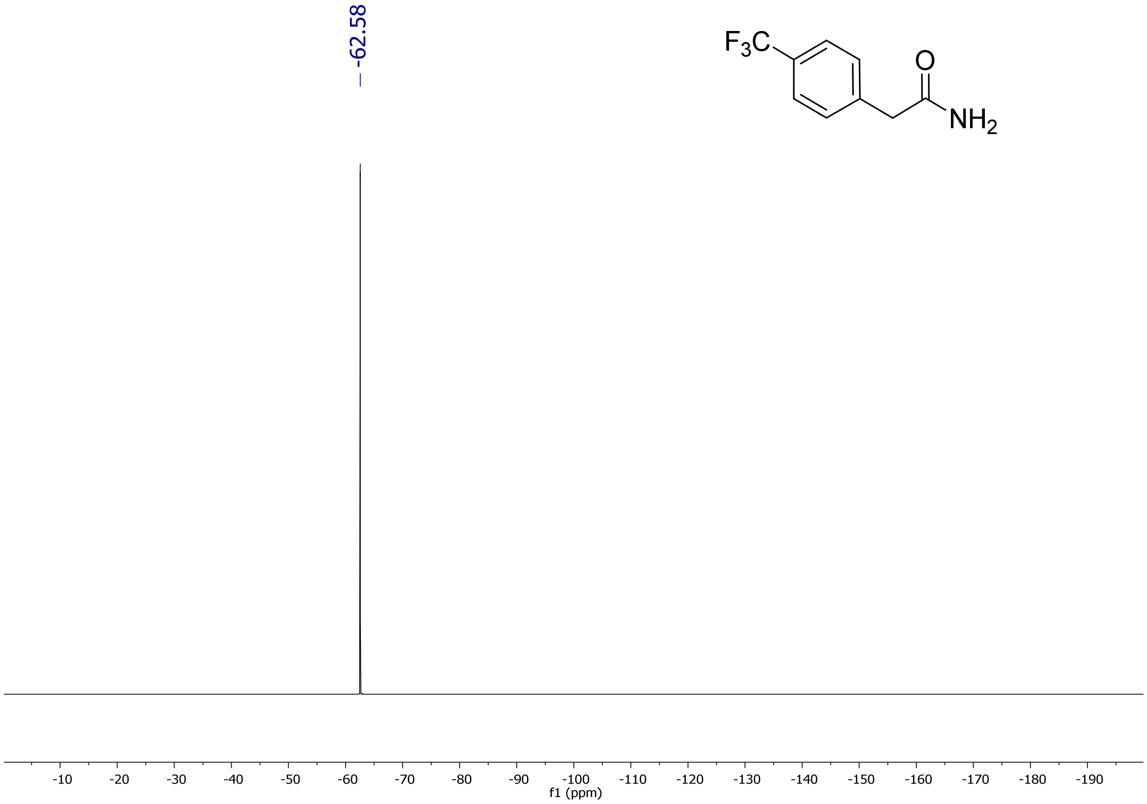

Figure S55. ^{19}F NMR (376.49 MHz, $CDCl_3$, 298 K) spectrum of 4-(trifluoromethyl)phenylacetamide.

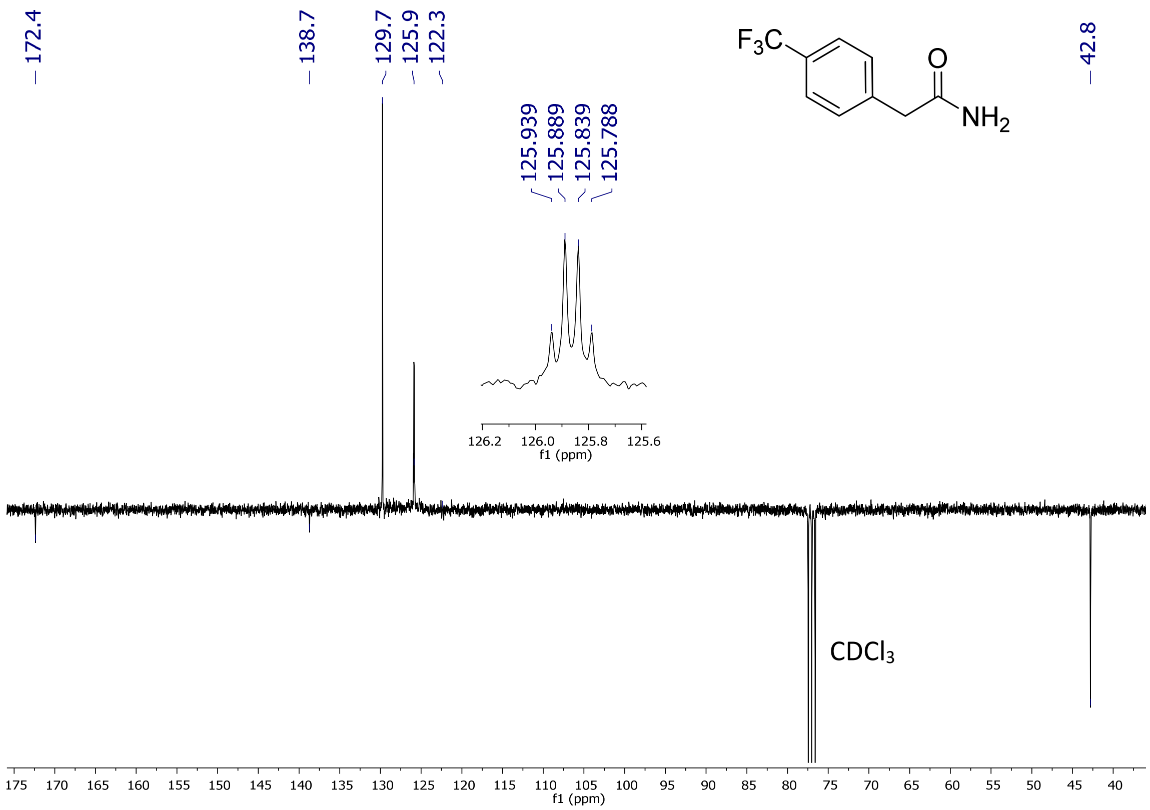

Figure S56. $^{13}C\{^1H\}$ APT NMR (75.48 MHz, $CDCl_3$, 298 K) spectrum of 4-(trifluoromethyl)phenylacetamide.

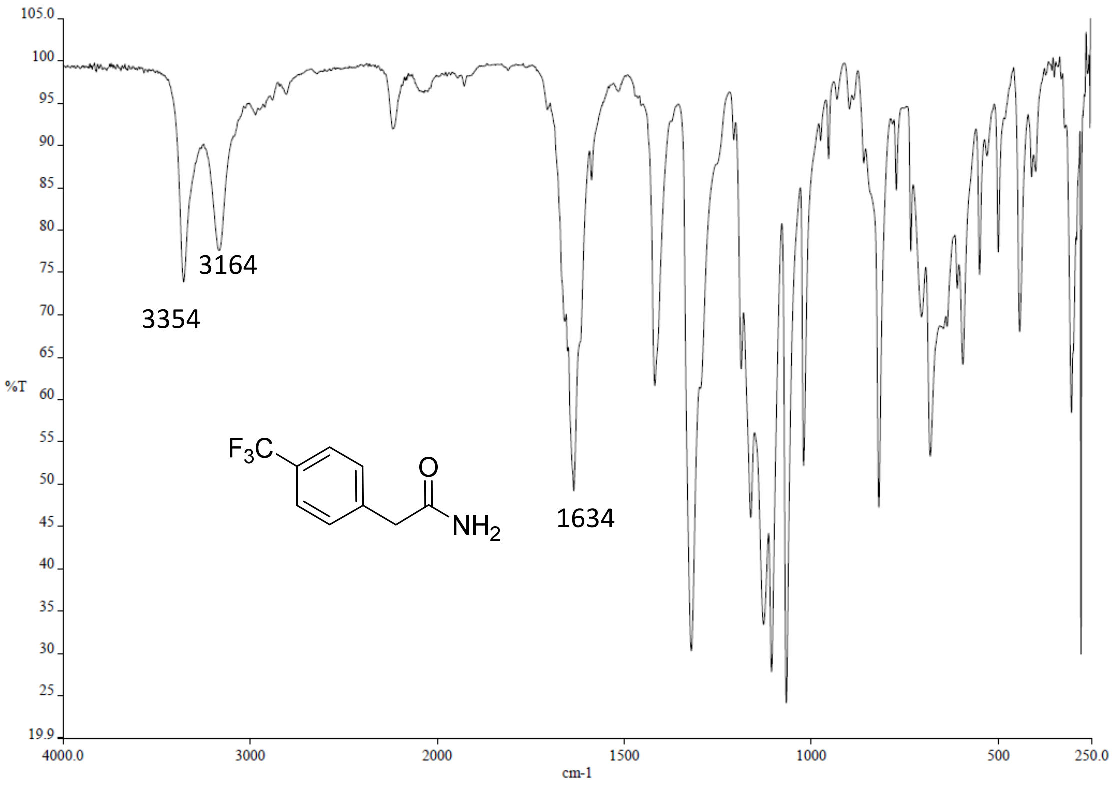

Figure S57. IR ATR spectrum of 4-(trifluoromethyl)phenylacetamide.

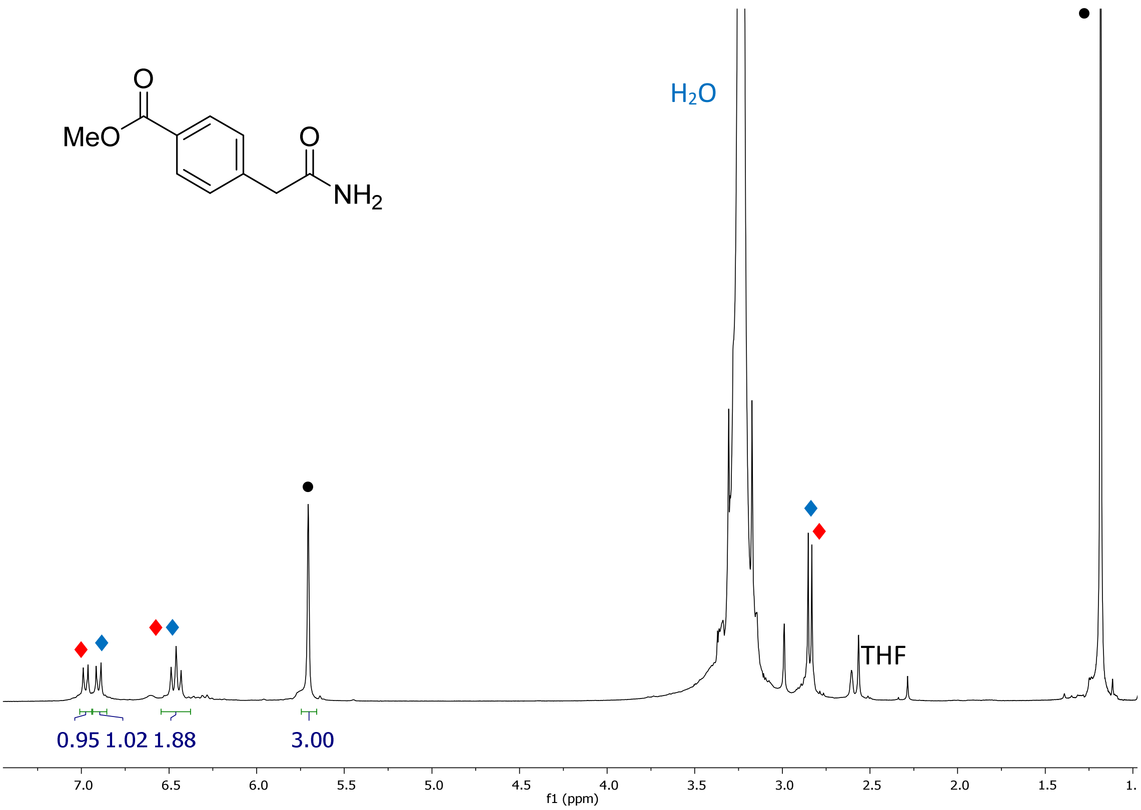

Figure S58. ^{1}H NMR (300.13 MHz, THF-*d*8, 298 K) spectrum of the reaction mixture of the hydration of methyl-4-(cyanomethyl)benzoate (♦): formation of methyl-4-(2-amino-2-oxoethyl)benzoate (♦). • Mesitylene.

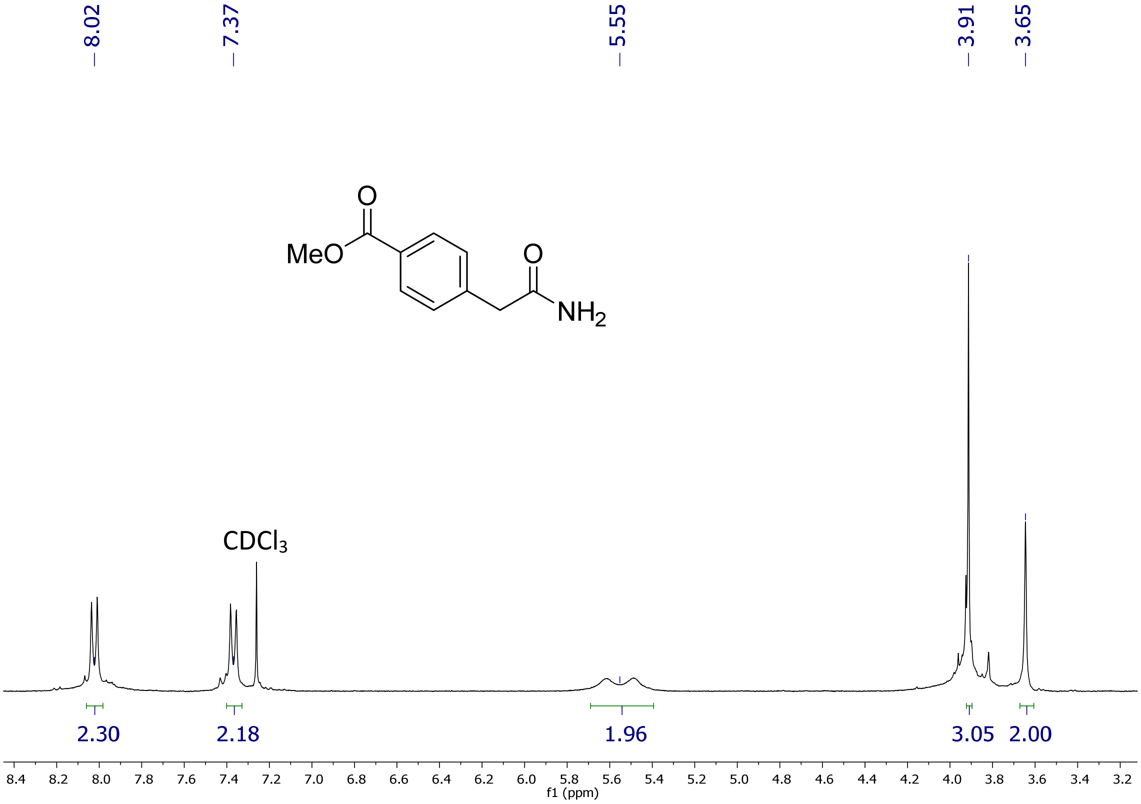

Figure S59. ^{1}H NMR (300.13 MHz, $CDCl_3$, 298 K) spectrum of methyl-4-(2-amino-2-oxoethyl)benzoate.

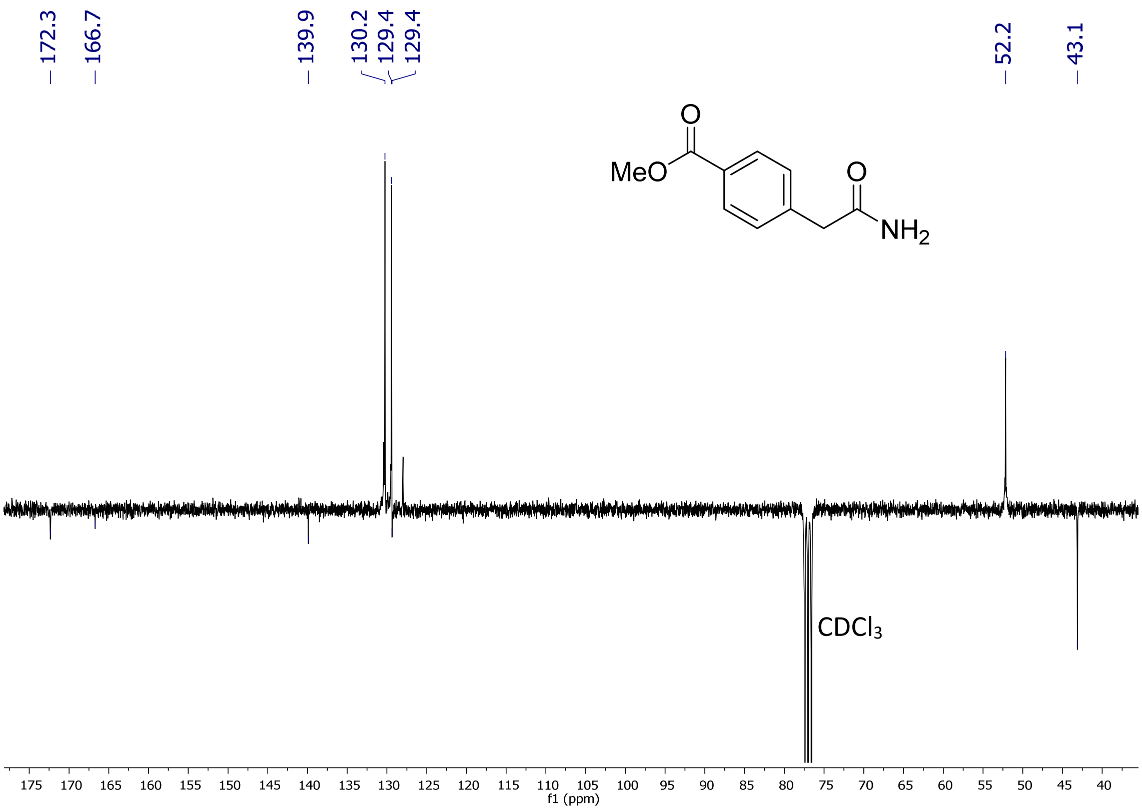

Figure S60. $^{13}C\{^{1}H\}$ APT NMR (75.48 MHz, $CDCl_3$, 298 K) spectrum of methyl-4-(2-amino-2-oxoethyl)benzoate.

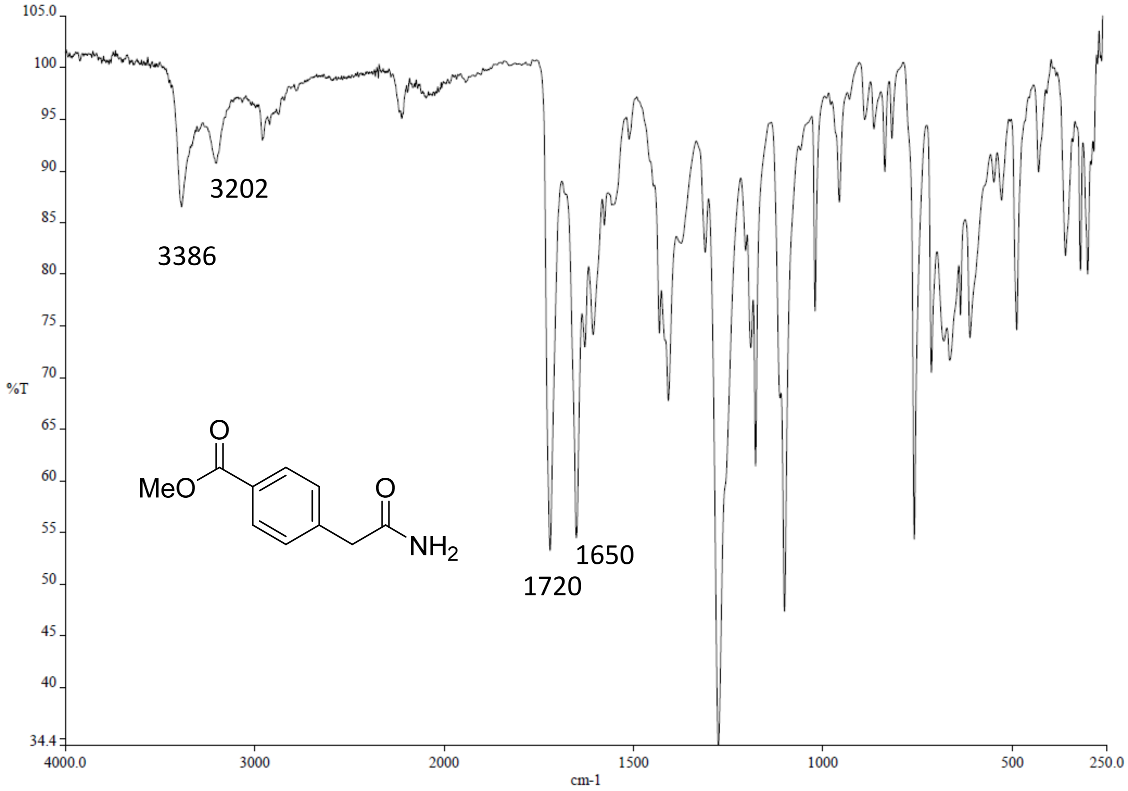

Figure S61. IR ATR spectrum of methyl-4-(2-amino-2-oxoethyl)benzoate.

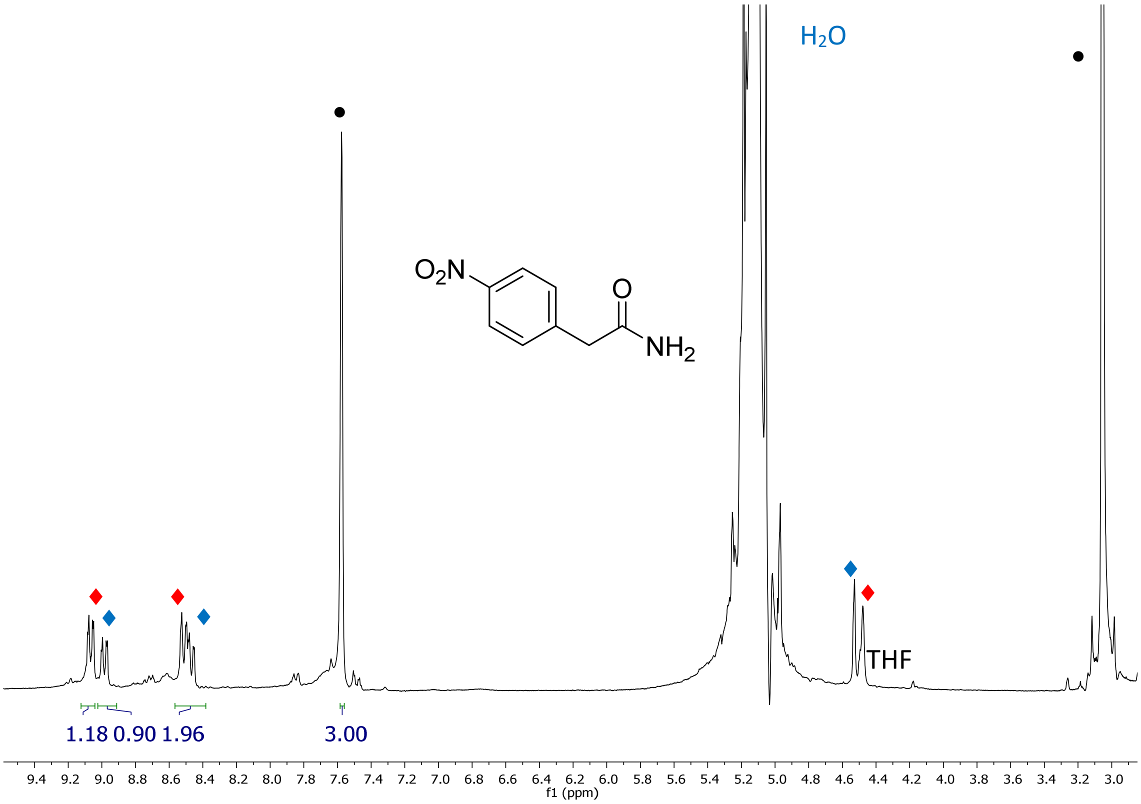

Figure S62. ^{1}H NMR (300.13 MHz, THF-d_8, 298 K) spectrum of the reaction mixture of the hydration of 4-nitrophenylacetonitrile (♦): formation of 4-nitrophenylacetamide (♦). • Mesitylene.

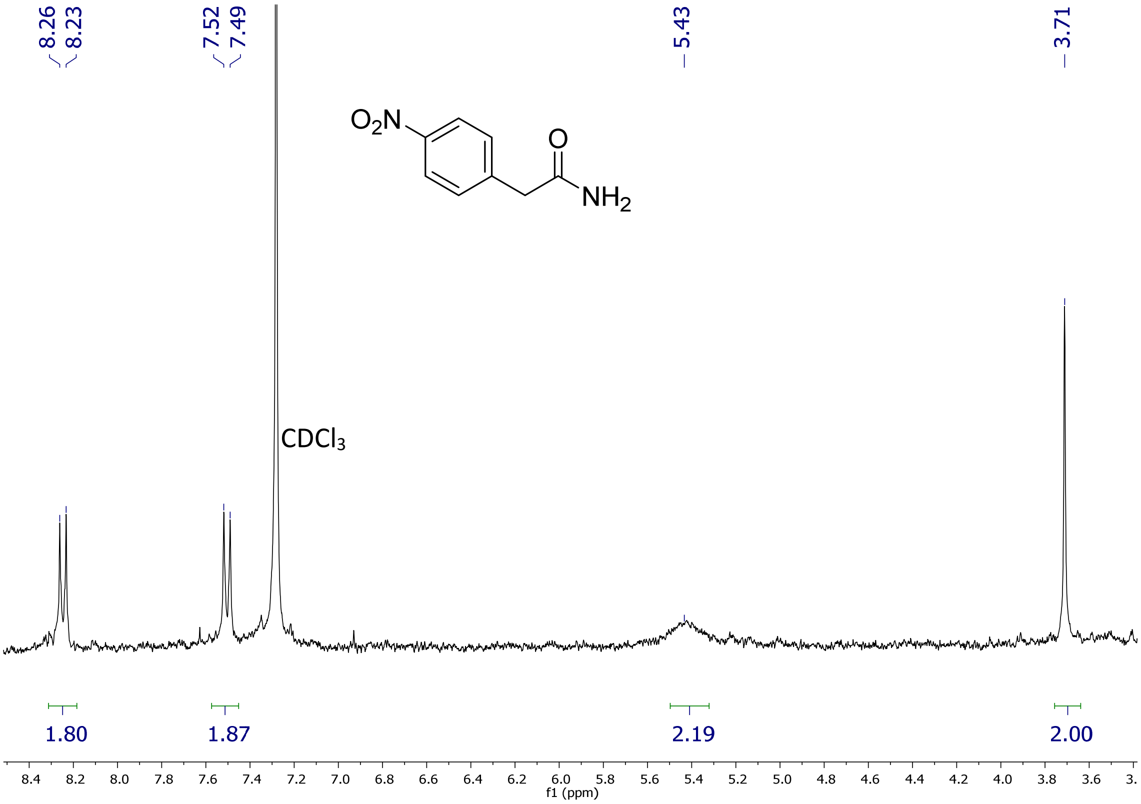

Figure S63. ^{1}H NMR (300.13 MHz, $CDCl_3$, 298 K) spectrum of 4-nitrophenylacetamide.

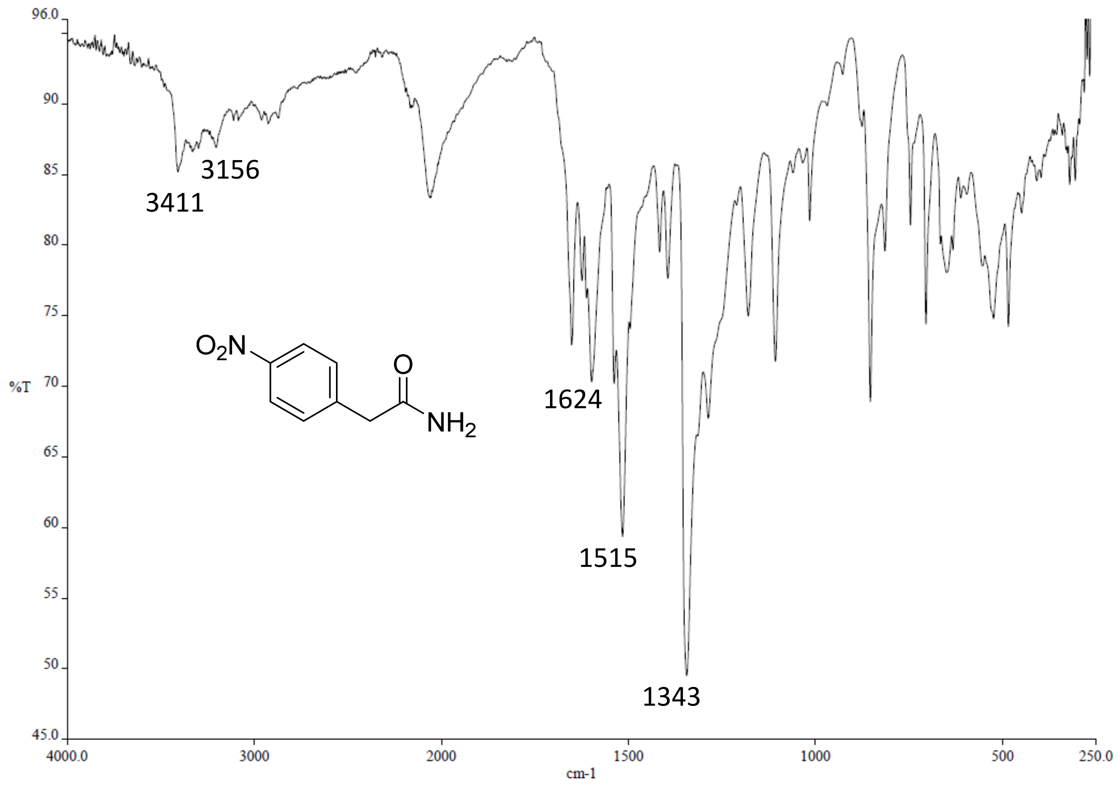

Figure S64. IR ATR spectrum of 4-nitrophenylacetamide.

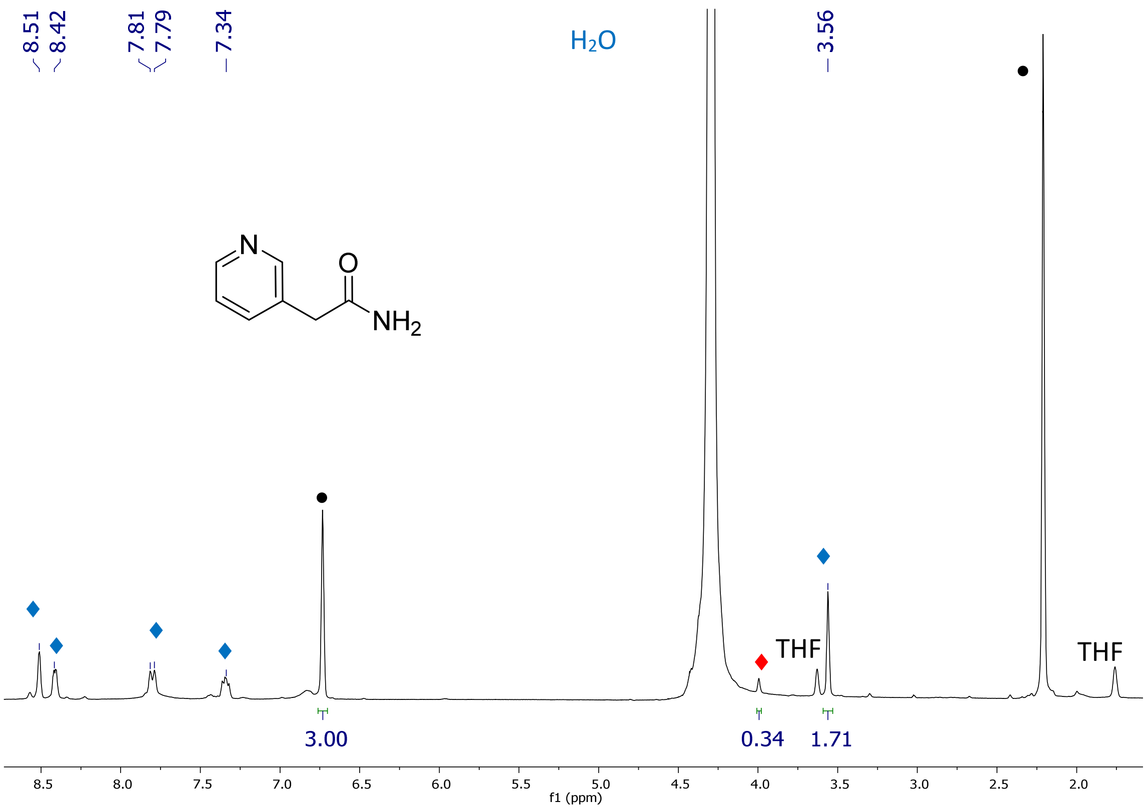

Figure S65. ^{1}H NMR (300.13 MHz, THF-d_8, 298 K) spectrum of the reaction mixture of the hydration of (pyridin-3-yl)acetonitrile (♦): formation of (pyridin-3-yl)acetamide (♦). ● Mesitylene.

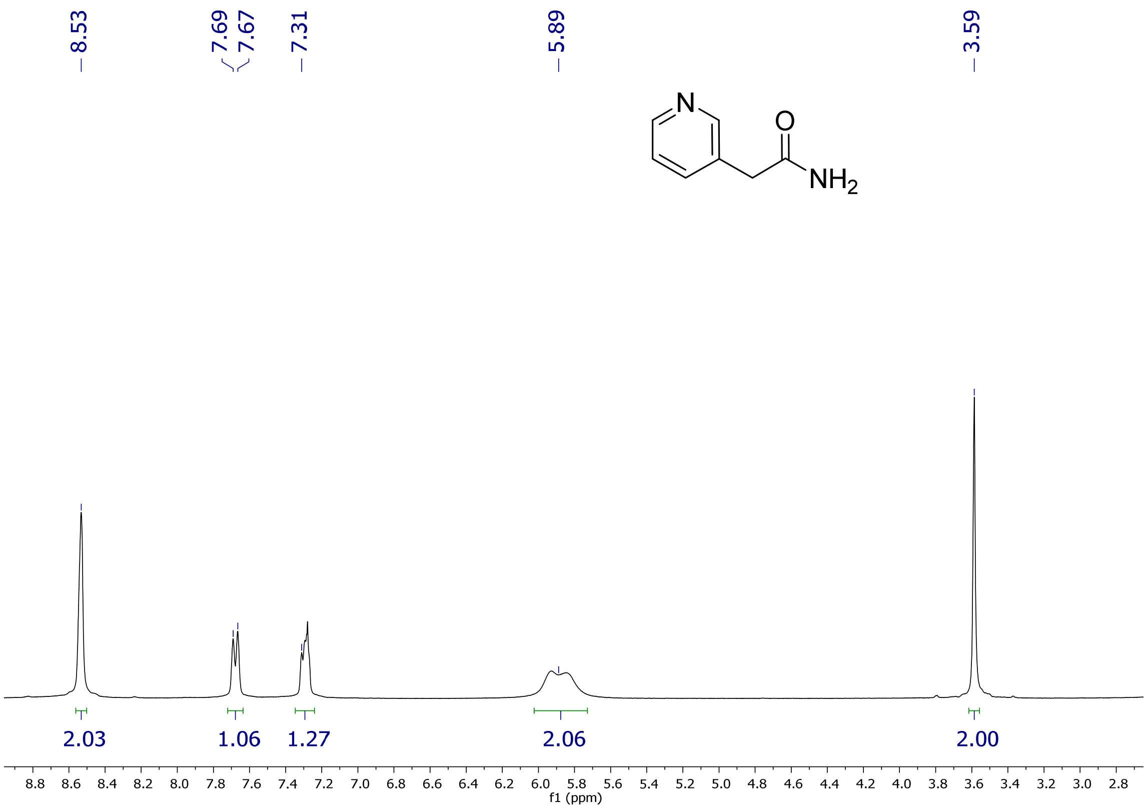

Figure S66. ^{1}H NMR (300.13 MHz, $CDCl_3$, 298 K) spectrum of (pyridin-3-yl)acetamide.

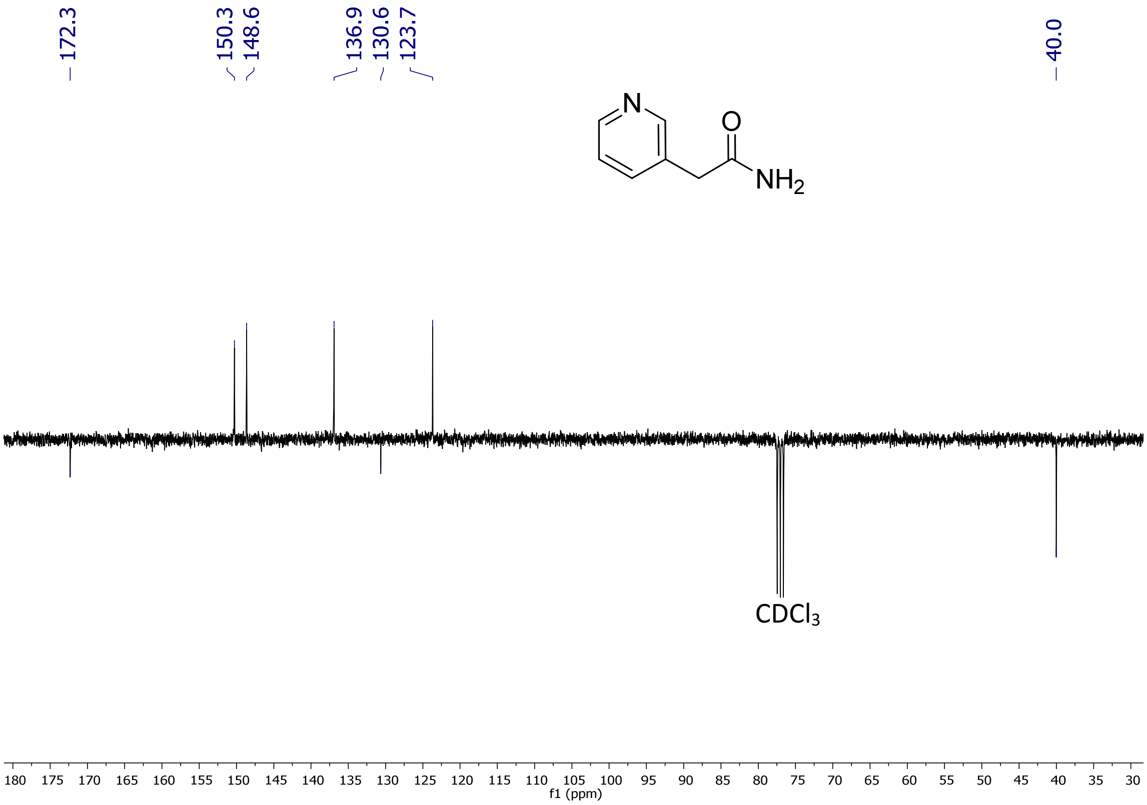

Figure S67. ^{13}C{^{1}H} APT NMR (75.48 MHz, $CDCl_3$, 298 K) spectrum of (pyridin-3-yl)acetamide.

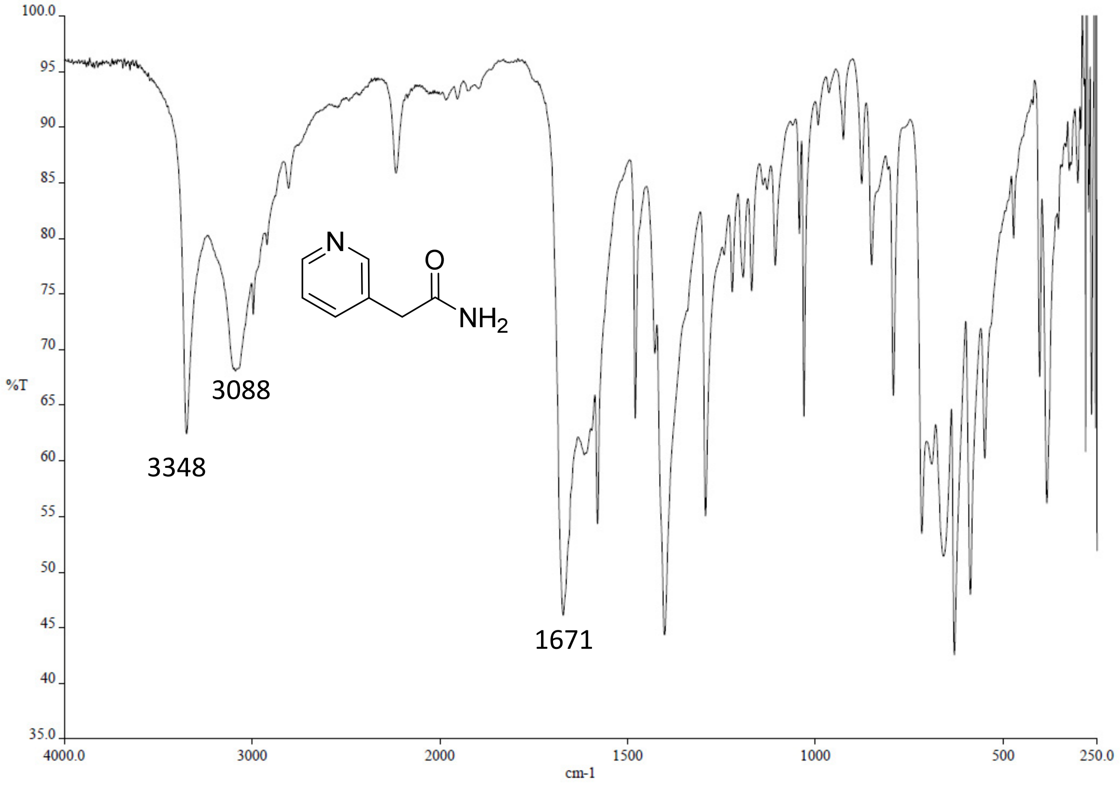

Figure S68. IR ATR spectrum of 2-(pyridin-3-yl)acetamide.

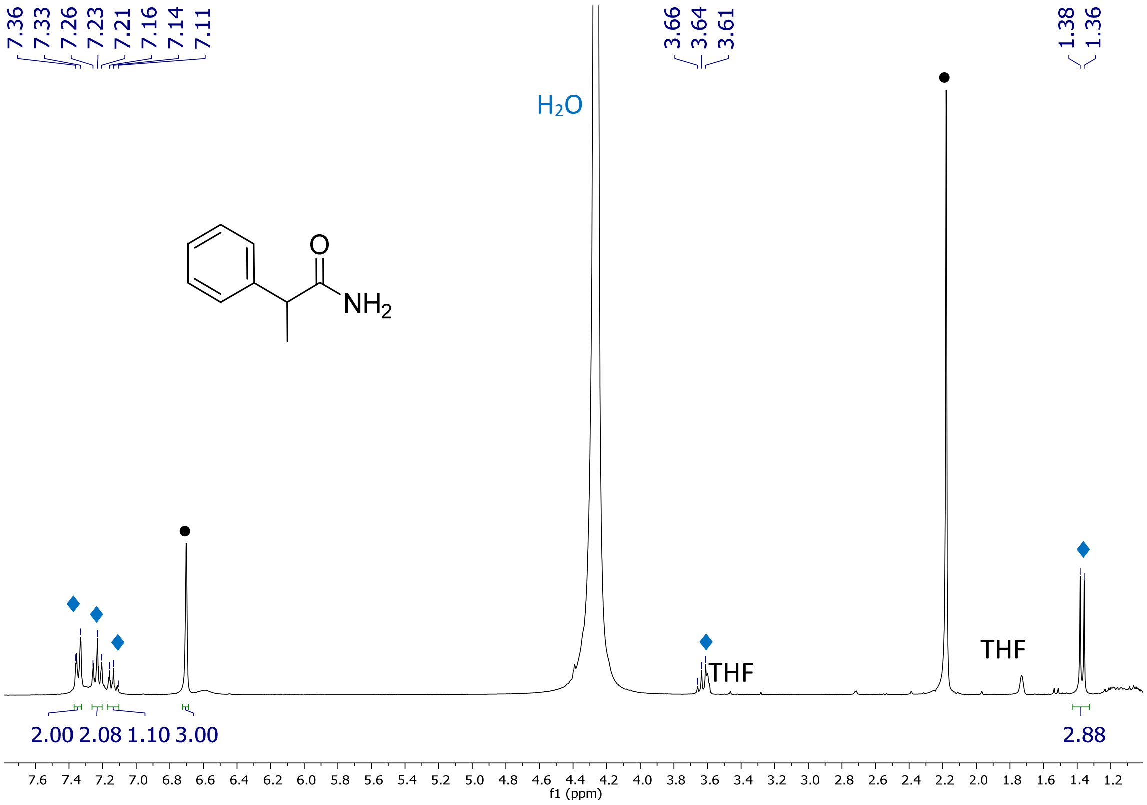

Figure S69. ^{1}H NMR (300.13 MHz, THF-d_8, 298 K) spectrum of the reaction mixture of the hydration of 2-phenylpropionitrile (♦): formation of 2-phenylpropanamide (♦). • Mesitylene.

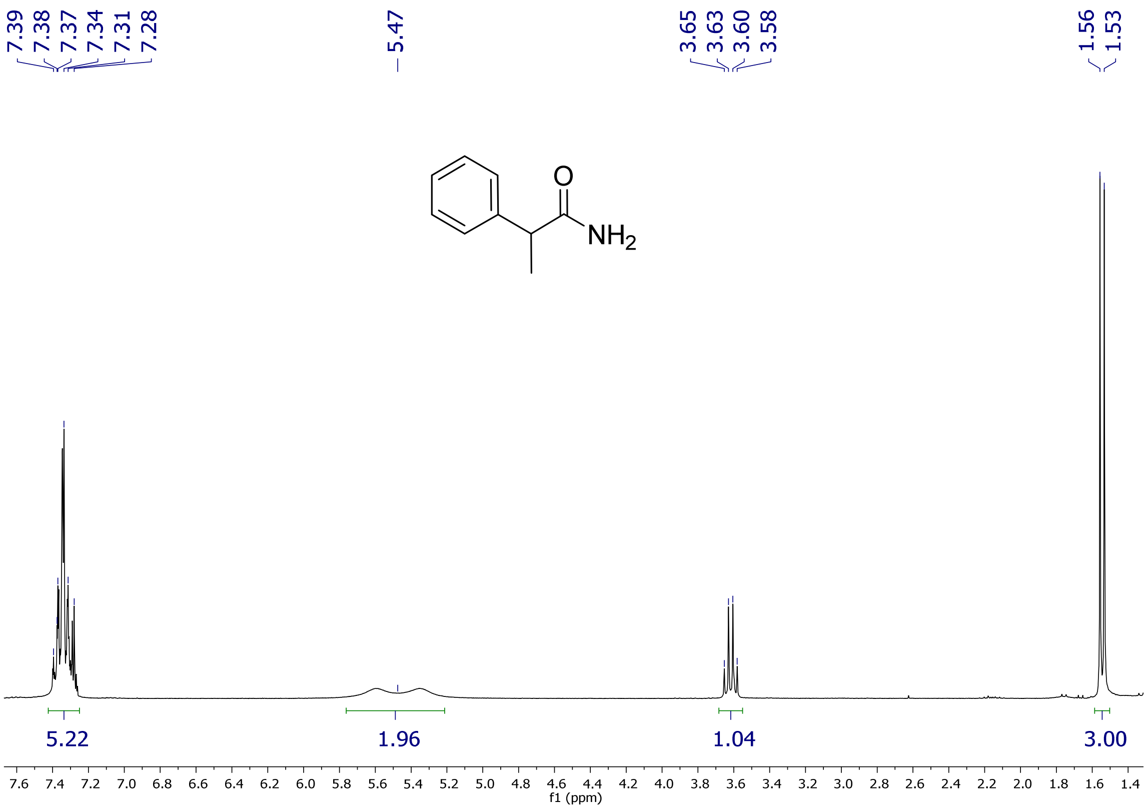

Figure S70. ^{1}H NMR (300.13 MHz, $CDCl_3$, 298 K) spectrum of 2-phenylpropanamide.

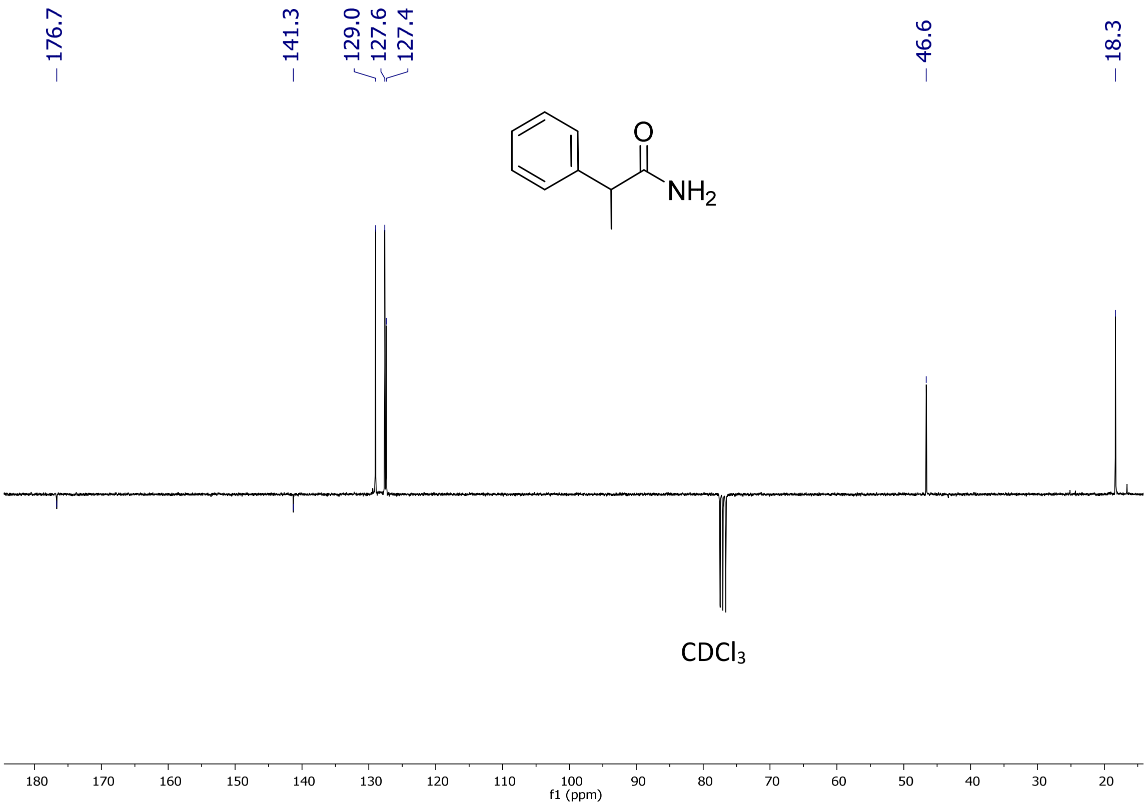

Figure S71. $^{13}C\{^{1}H\}$ APT NMR (75.48 MHz, $CDCl_3$, 298 K) spectrum of 2-phenylpropanamide.

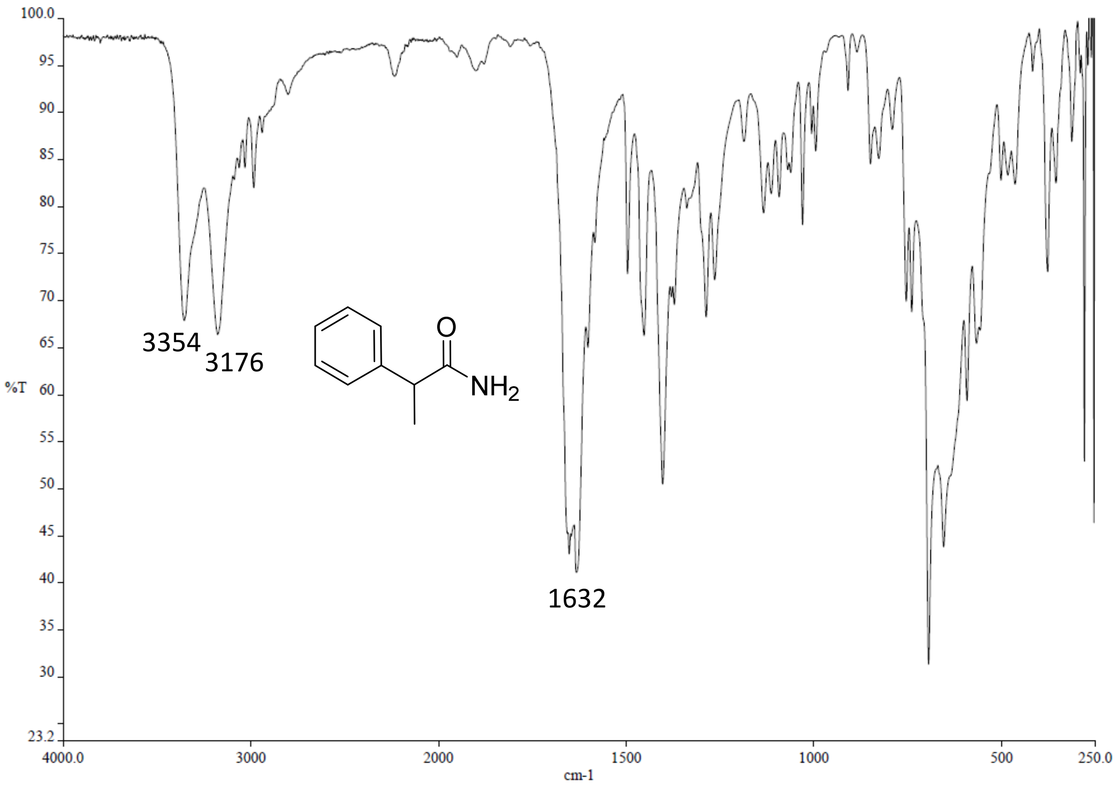

Figure S72. IR ATR spectrum of 2-phenylpropanamide.

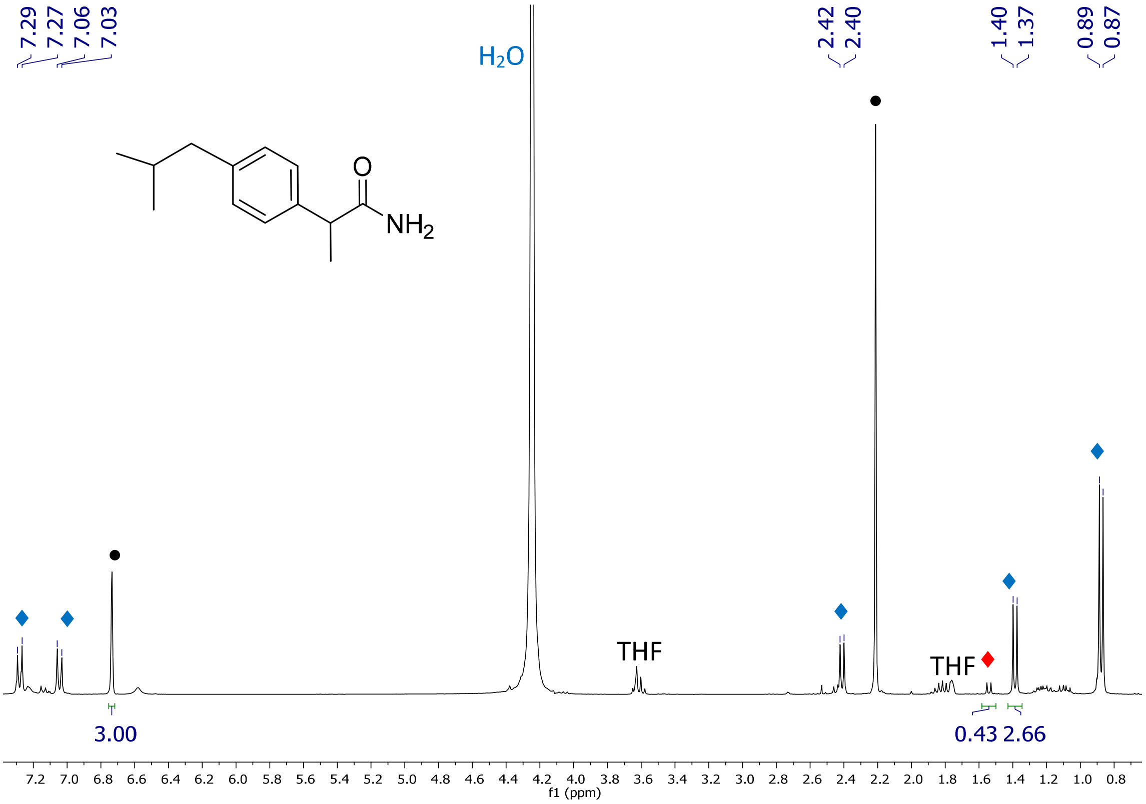

Figure S73. ^{1}H NMR (300.13 MHz, THF-d_8, 298 K) spectrum of the reaction mixture of the hydration of 2-(3-isobutylphenyl)propanenitrile (♦): formation of 2-(3-isobutylphenyl)propanamide (♦). • Mesitylene

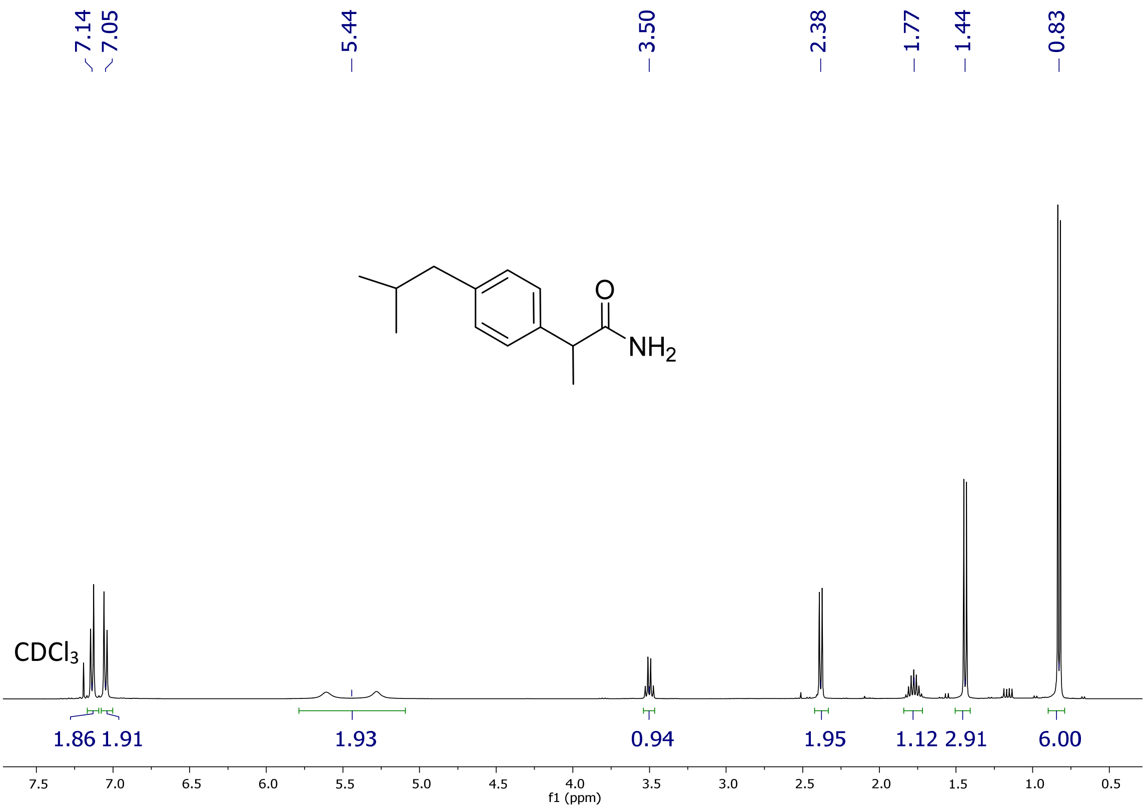

Figure S74. ^{1}H NMR (400.16 MHz, $CDCl_3$, 298 K) spectrum of 2-(3-isobutylphenyl)propanamide.

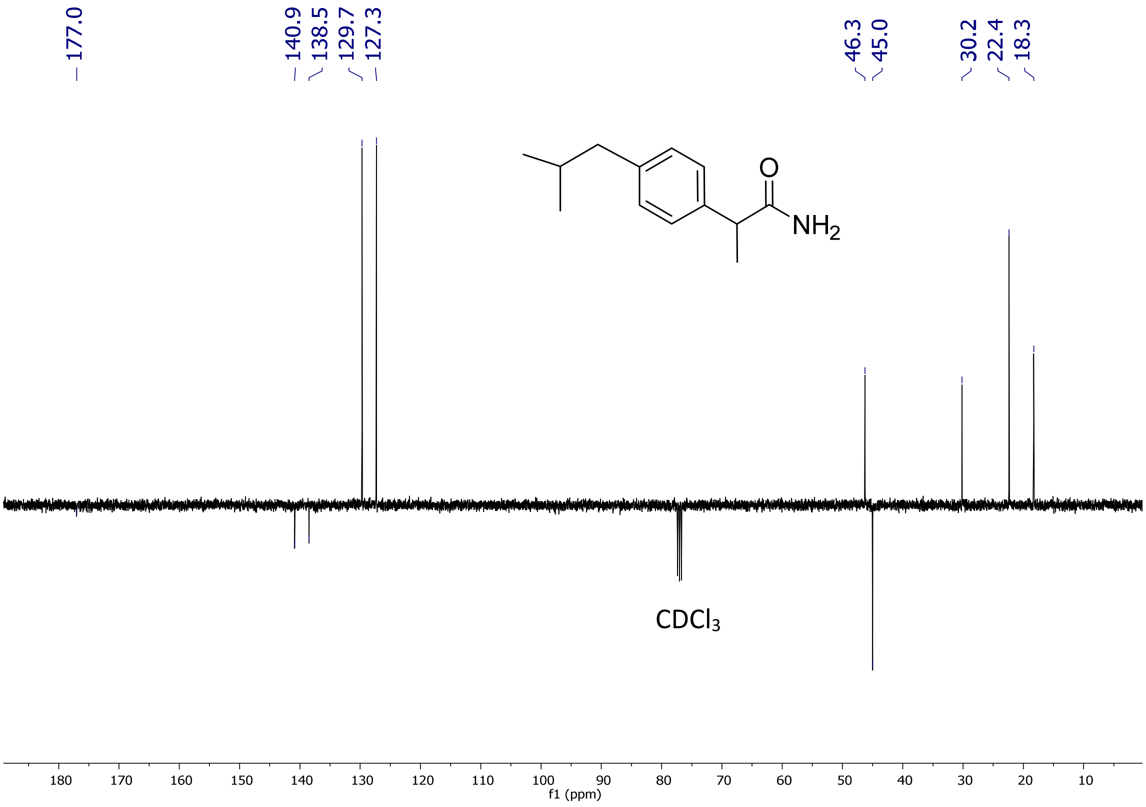

Figure S75. $^{13}C\{^1H\}$ APT NMR (100.63 MHz, $CDCl_3$, 298 K) spectrum of 2-(3-isobutylphenyl)propanamide.

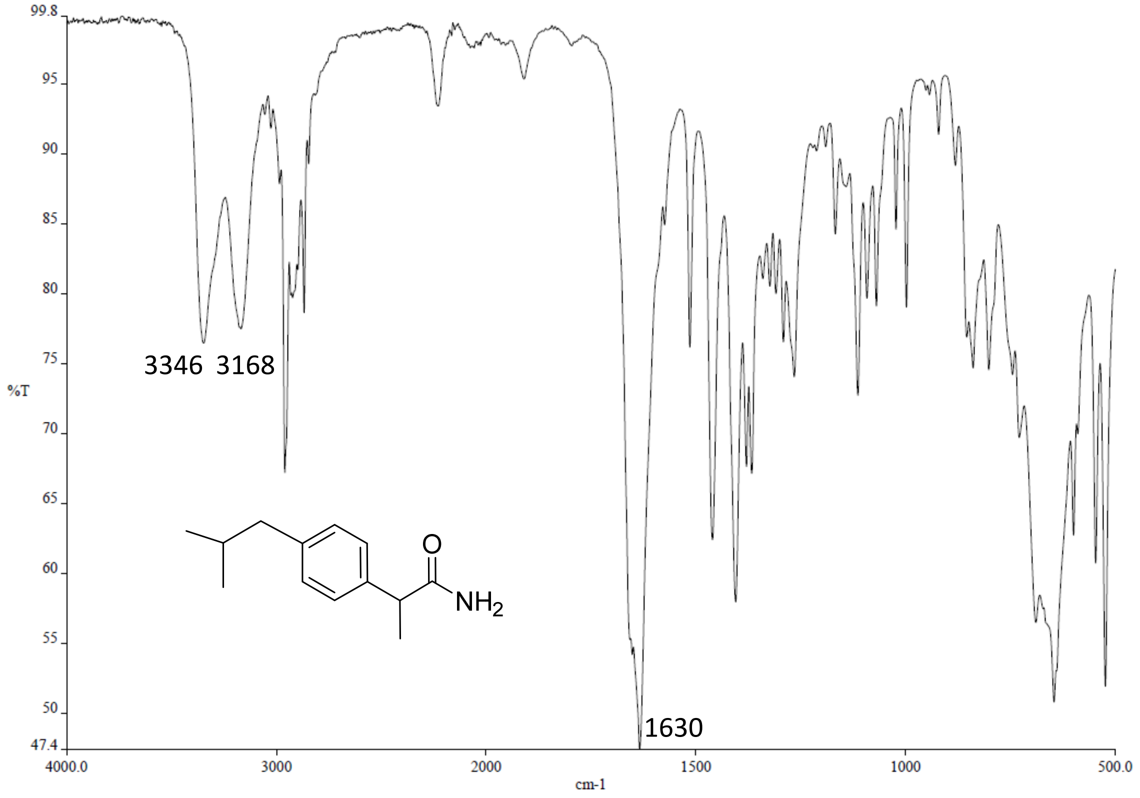

Figure S76. IR ATR spectrum of 2-(3-isobutylphenyl)propanamide.

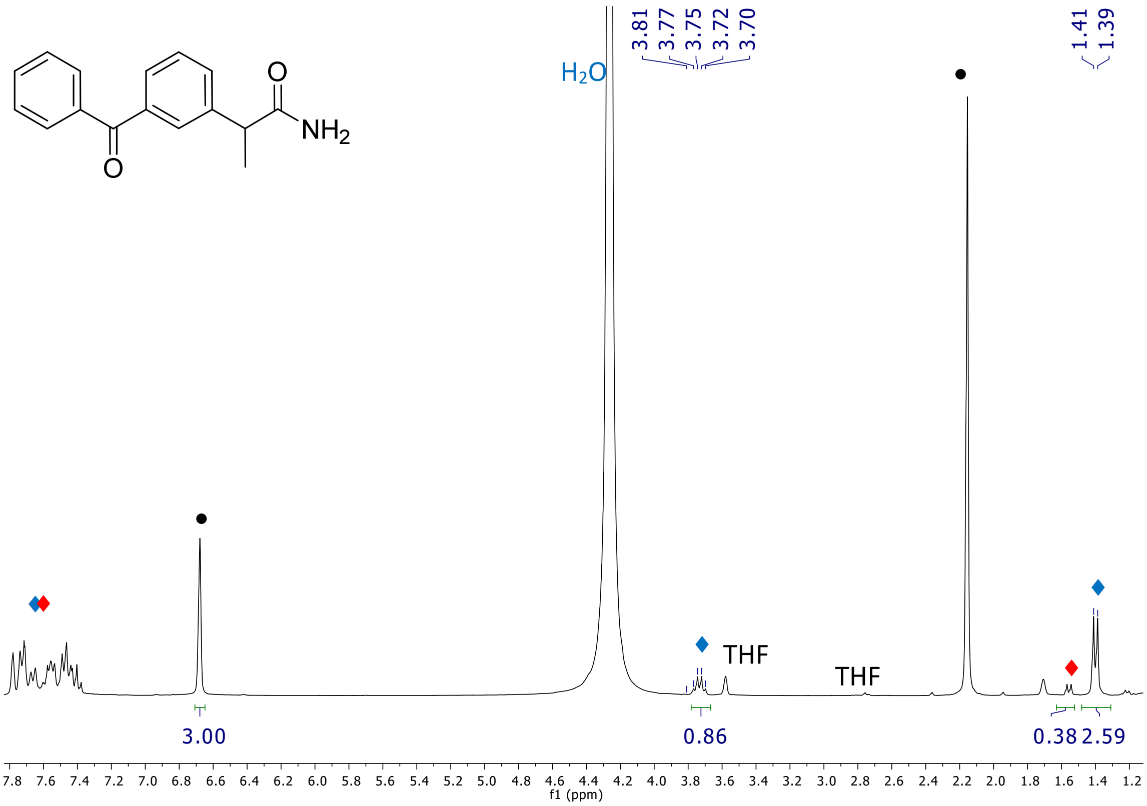

Figure S77. ^{1}H NMR (300.13 MHz, THF-d_8, 298 K) spectrum of the reaction mixture of the hydration of 2-(3-benzoylphenyl)propionitrile (♦): formation of 2-(3-benzoylphenyl)-propanamide (♦). • Mesitylene

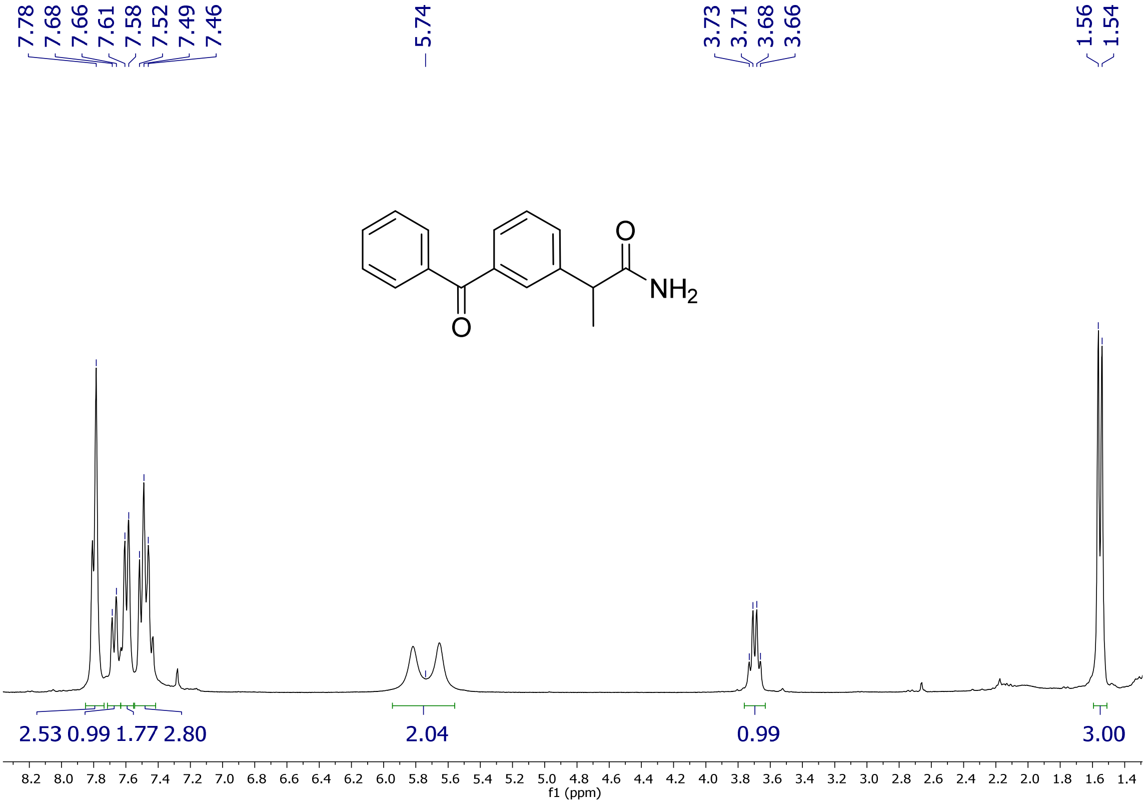

Figure S78. ^{1}H NMR (300.13 MHz, $CDCl_3$, 298 K) spectrum of 2-(3-benzoylphenyl)propanamide.

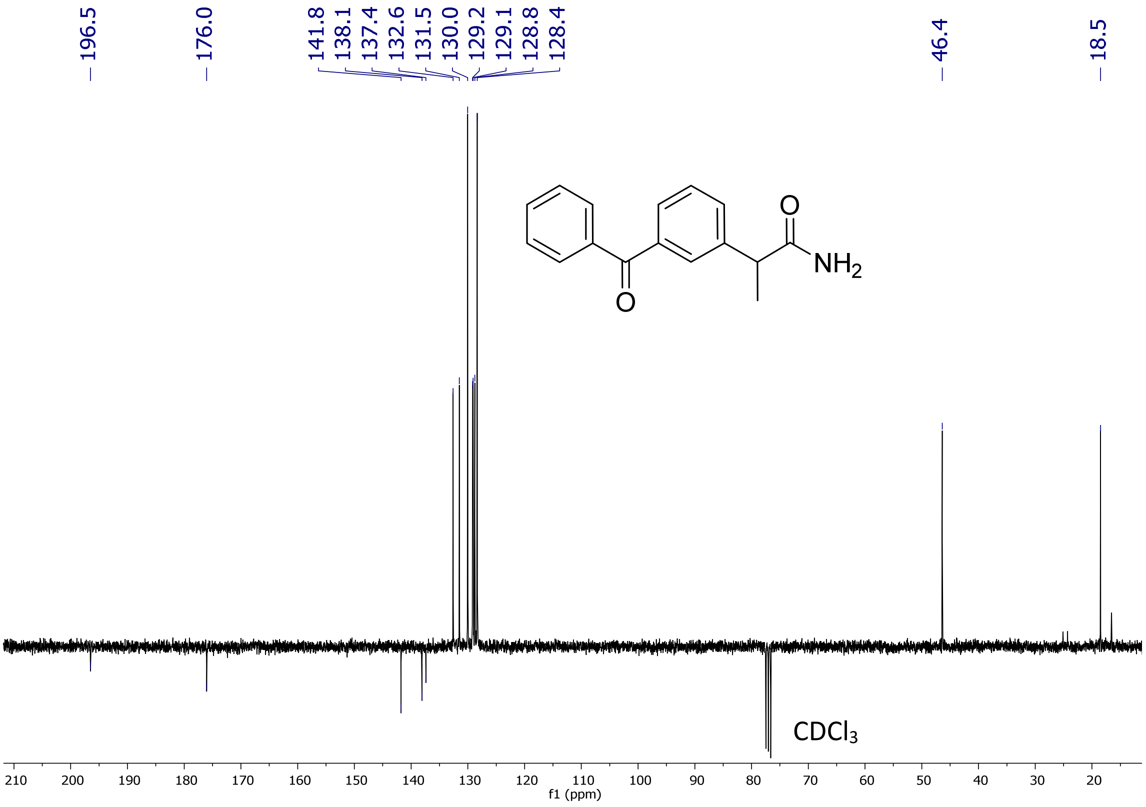

Figure S79. $^{13}C\{^{1}H\}$ APT NMR (75.48 MHz, $CDCl_3$, 298 K) spectrum of 2-(3-benzoylphenyl)propanamide.

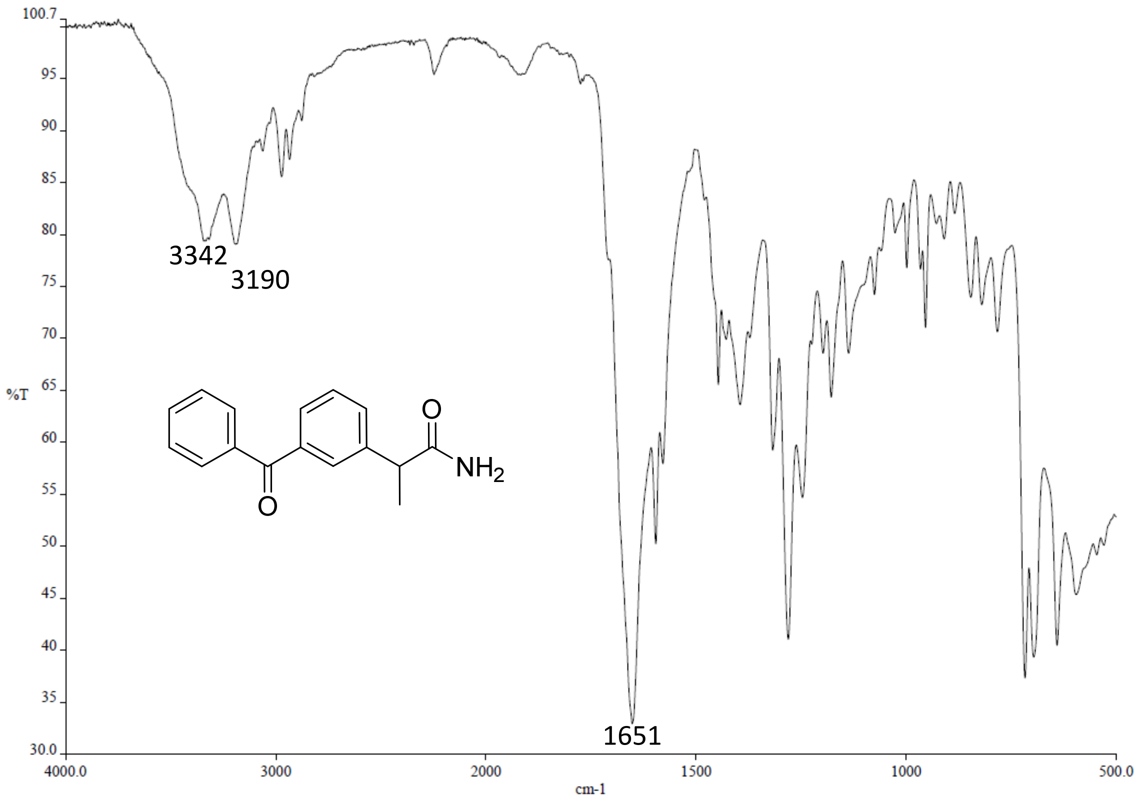

Figure S80. IR ATR spectrum of 2-(3-benzoylphenyl)propanamide.

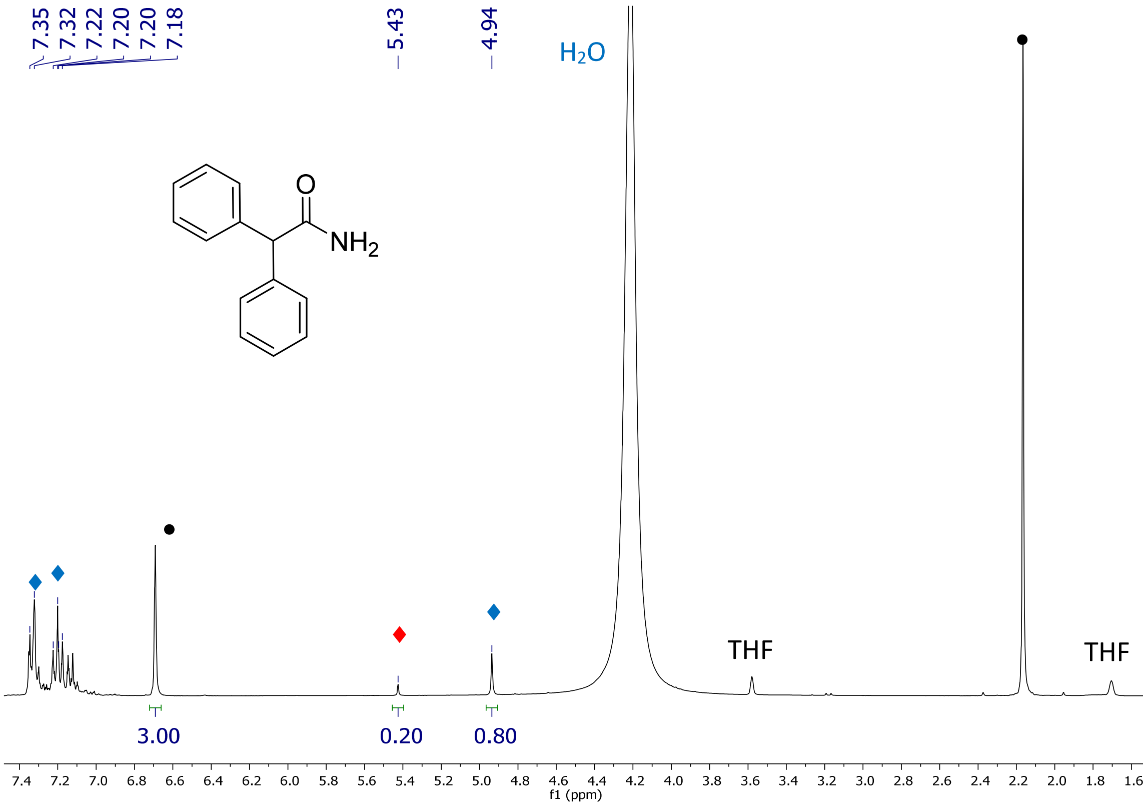

Figure S81. ^{1}H NMR (300.13 MHz, THF-d_8, 298 K) spectrum of the reaction mixture of the hydration of 2,2-diphenylacetonitrile (♦): formation of 2,2-diphenylacetamide (♦). • Mesitylene.

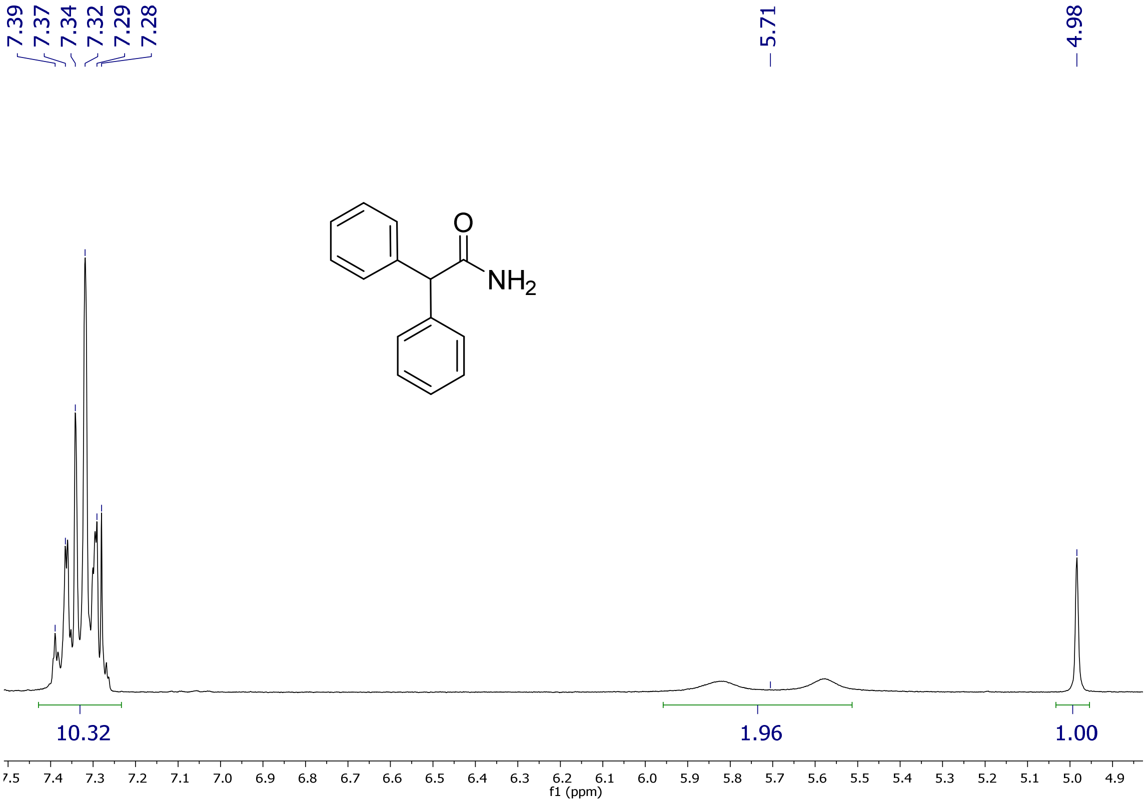

Figure S82. ^{1}H NMR (300.13 MHz, $CDCl_3$, 298 K) spectrum of 2,2-diphenylacetamide.

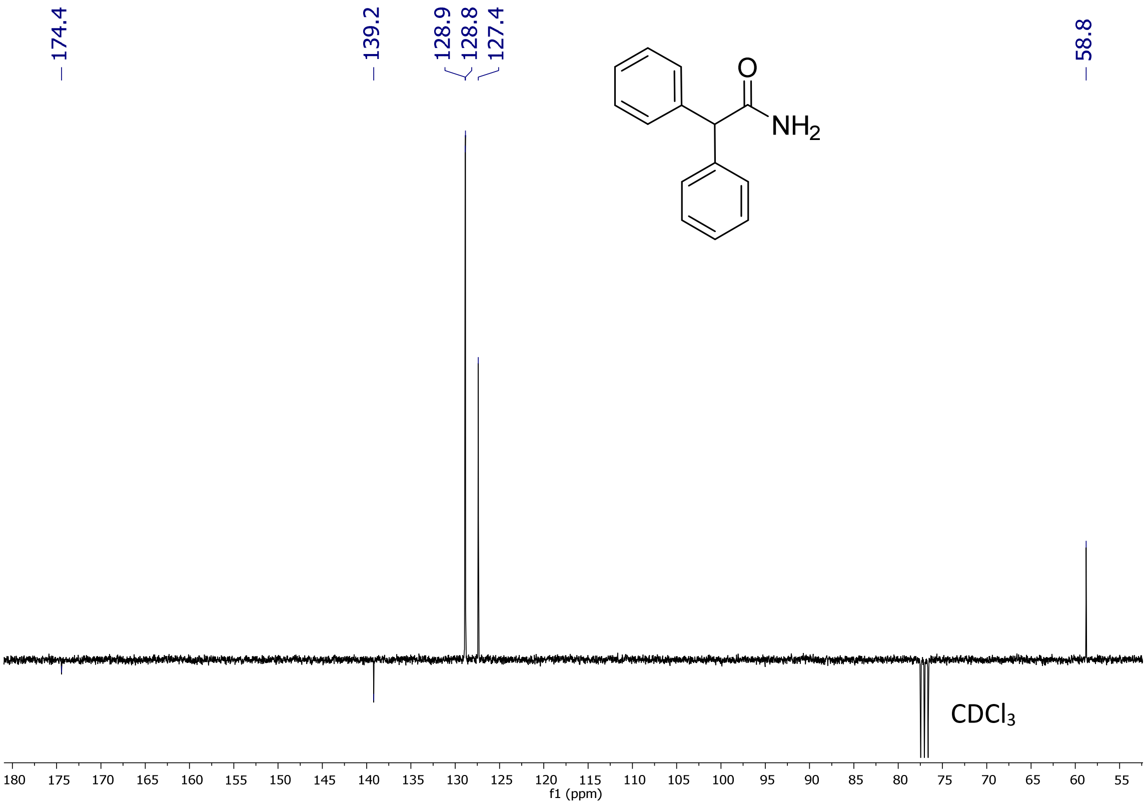

Figure S83. ^{13}C{^{1}H} APT NMR (75.48 MHz, $CDCl_3$, 298 K) spectrum of 2,2-diphenylacetamide.

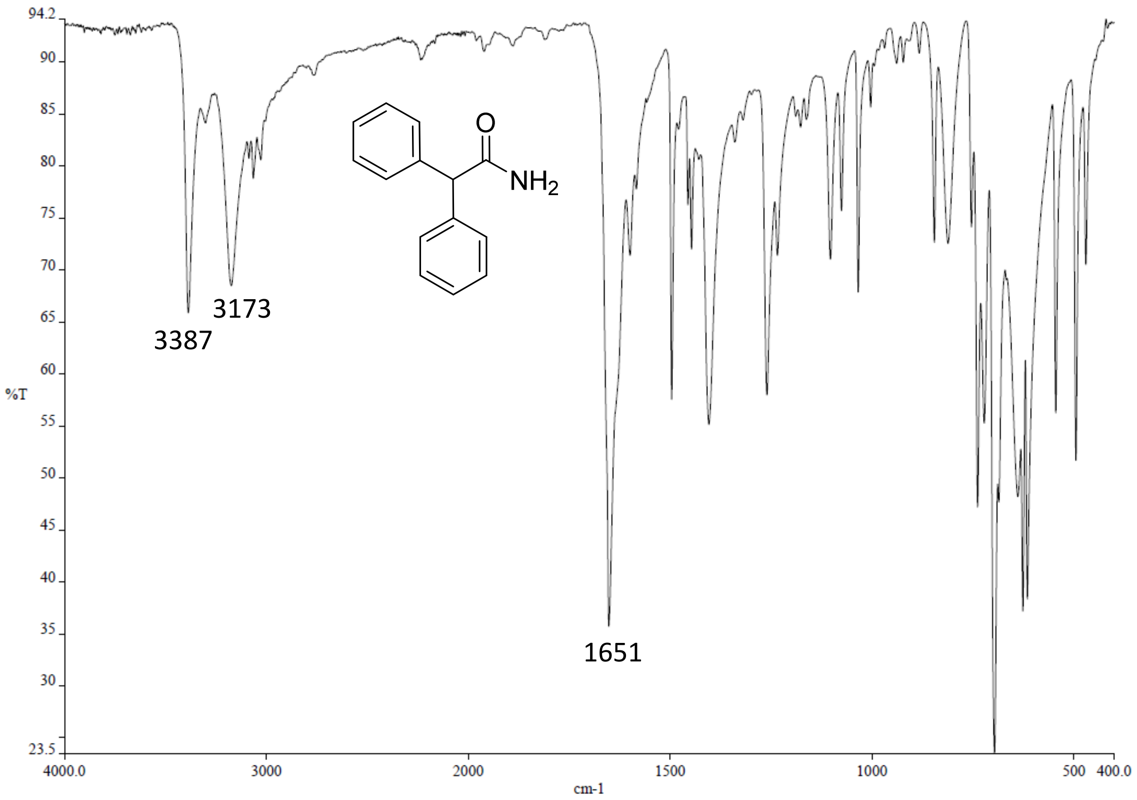

Figure S84. IR ATR spectrum of 2,2-diphenylacetamide.

^{1}H, ^{31}P {^{1}H}, ^{13}C{^{1}H} APT NMR spectra of complexes 2a and 2b.

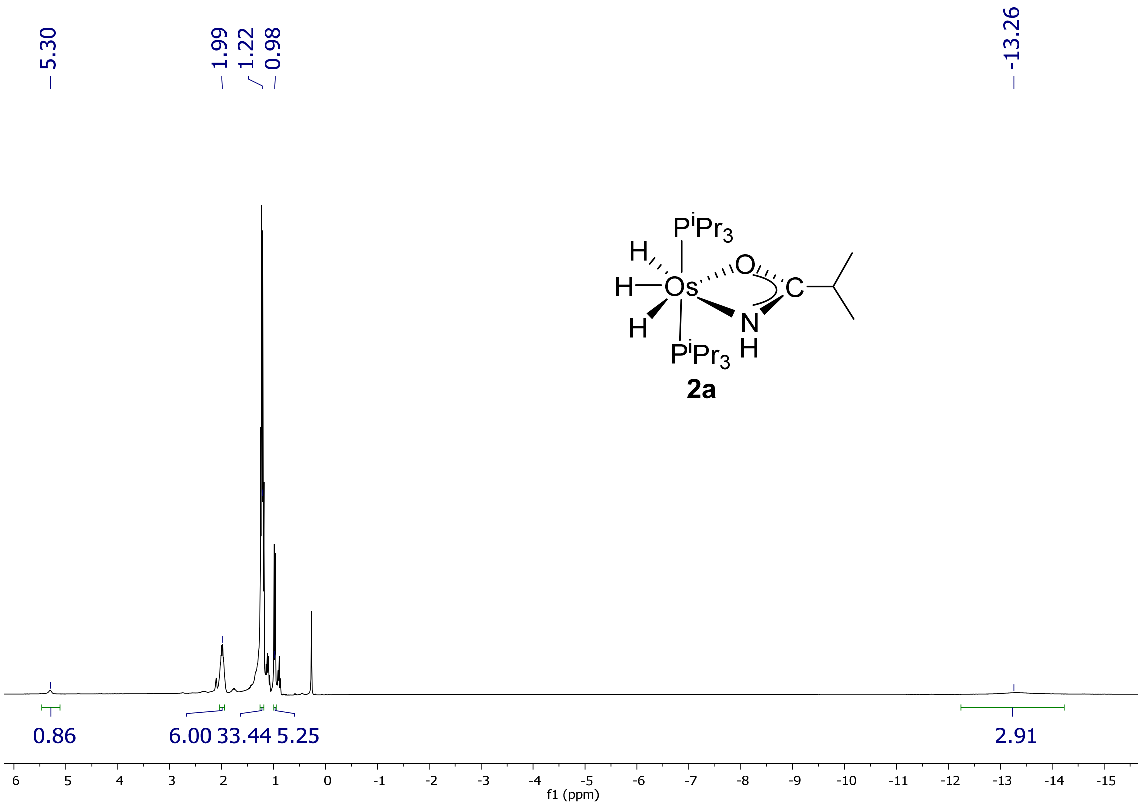

Figure S85. ^{1}H NMR (300.13 MHz, C_7D_8, 298 K) spectrum for complex **2a**.

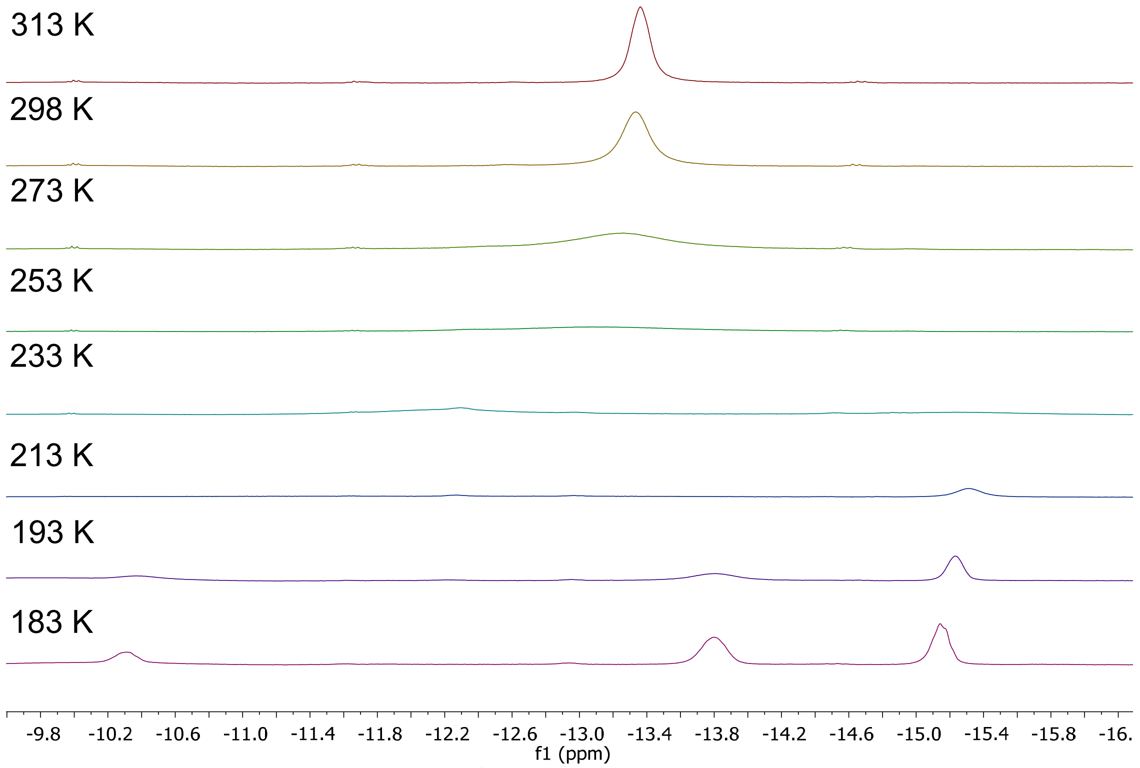

Figure S86. High-field region of the ^{1}H NMR (300.13 MHz, C_7D_8) spectrum of complex **2a** between 313 K and 183 K.

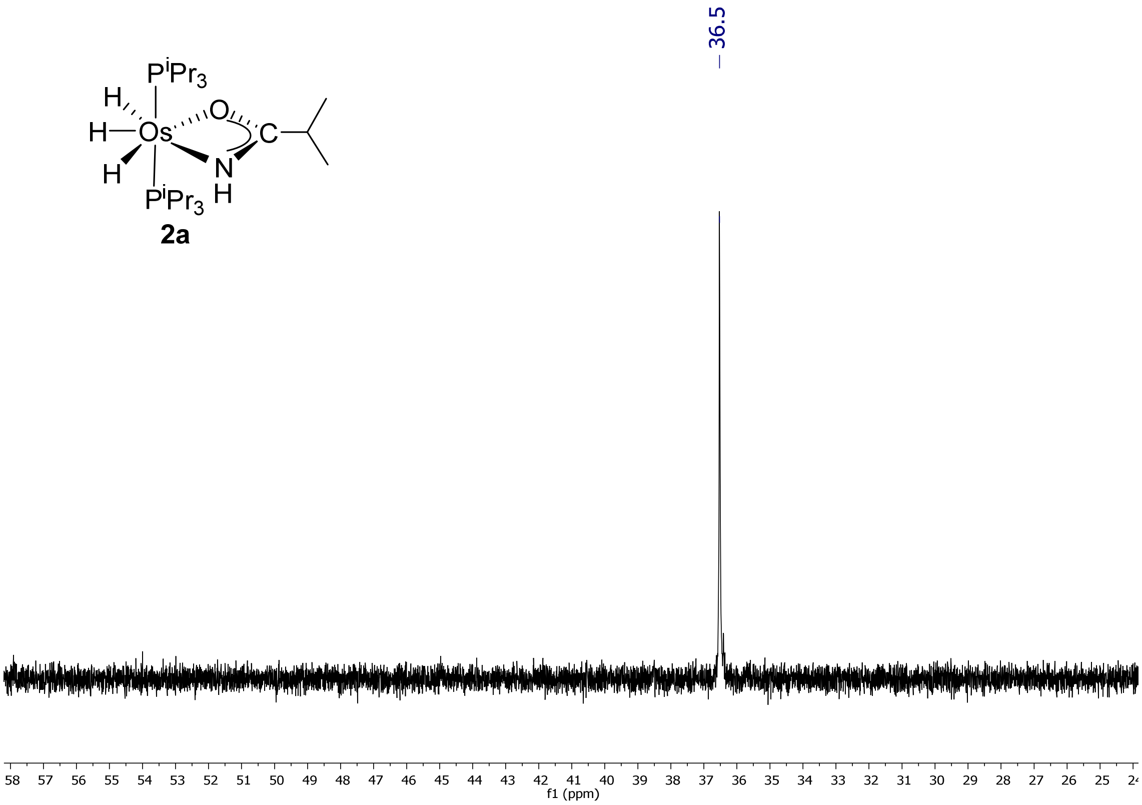

Figure S87. ^{31}P{^{1}H} NMR (121.50 MHz, C_7D_8, 298 K) spectrum for complex **2a**.

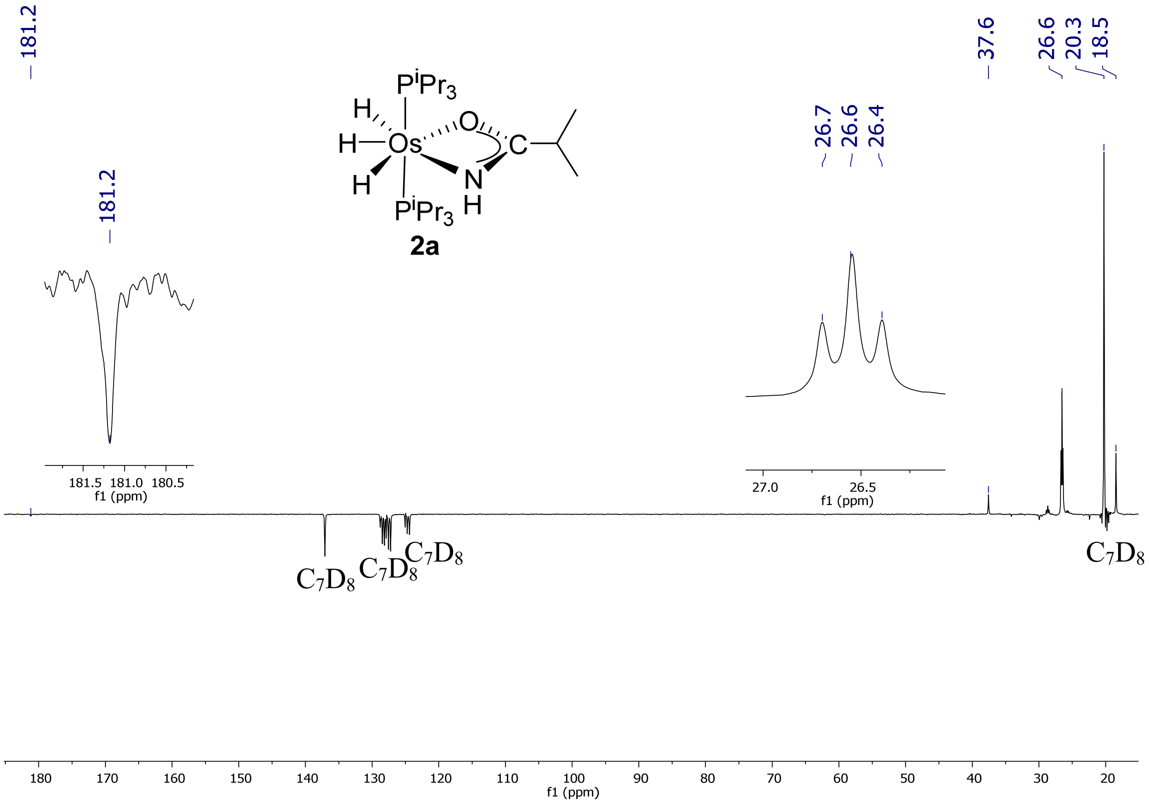

Figure S88. $^{13}C\{^{1}H\}$ APT NMR (75.48 MHz, $CDCl_3$, 298 K) spectrum of complex **2a**.

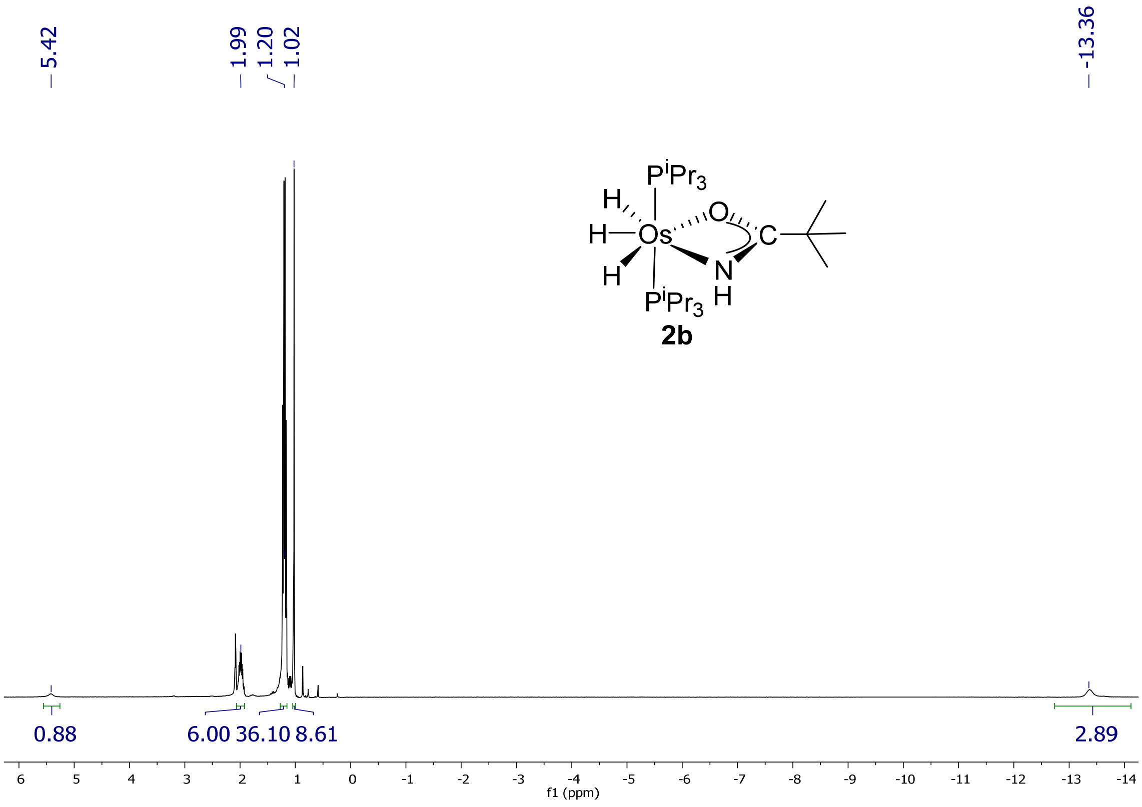

Figure S89. ^{1}H NMR (300.13 MHz, $CDCl_3$, 298 K) spectrum for complex **2b**.

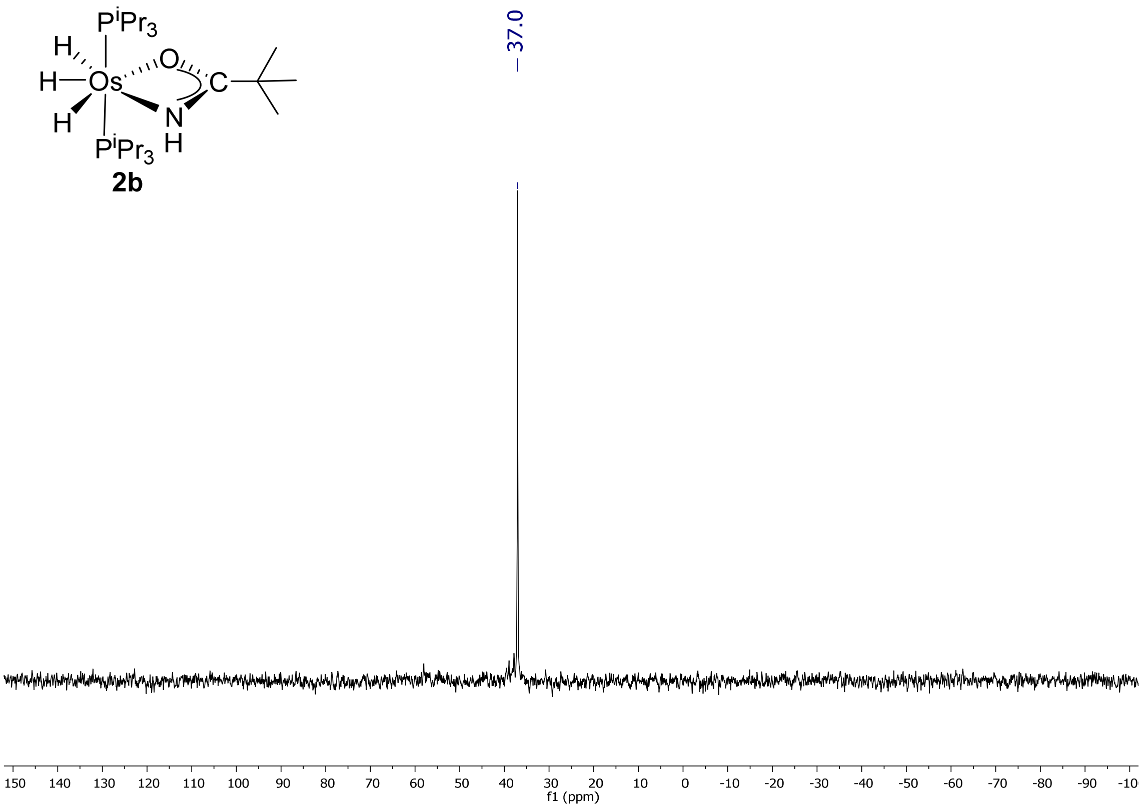

Figure S90. $^{31}P\{^{1}H\}$ NMR (121.50 MHz, C_7D_8, 298 K) spectrum for complex **2b**.

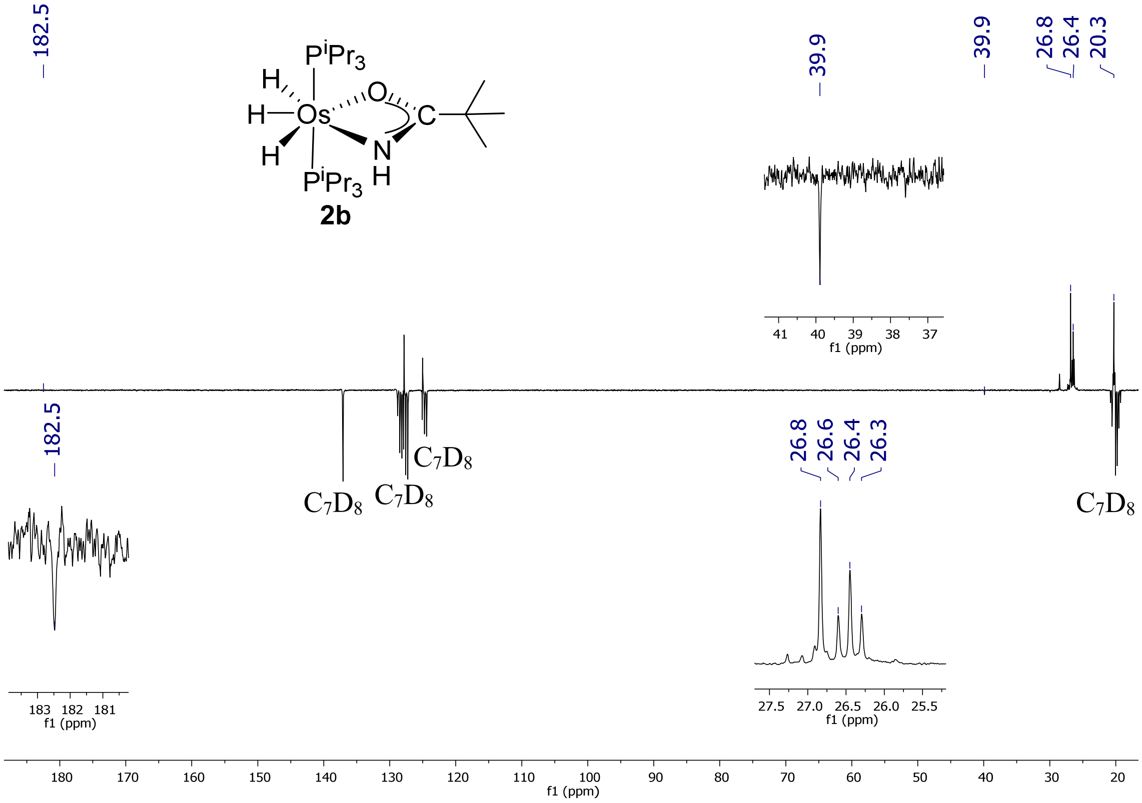

Figure S91. $^{13}C\{^{1}H\}$ APT NMR (75.48 MHz, $CDCl_3$, 298 K) spectrum of complex **2b**.

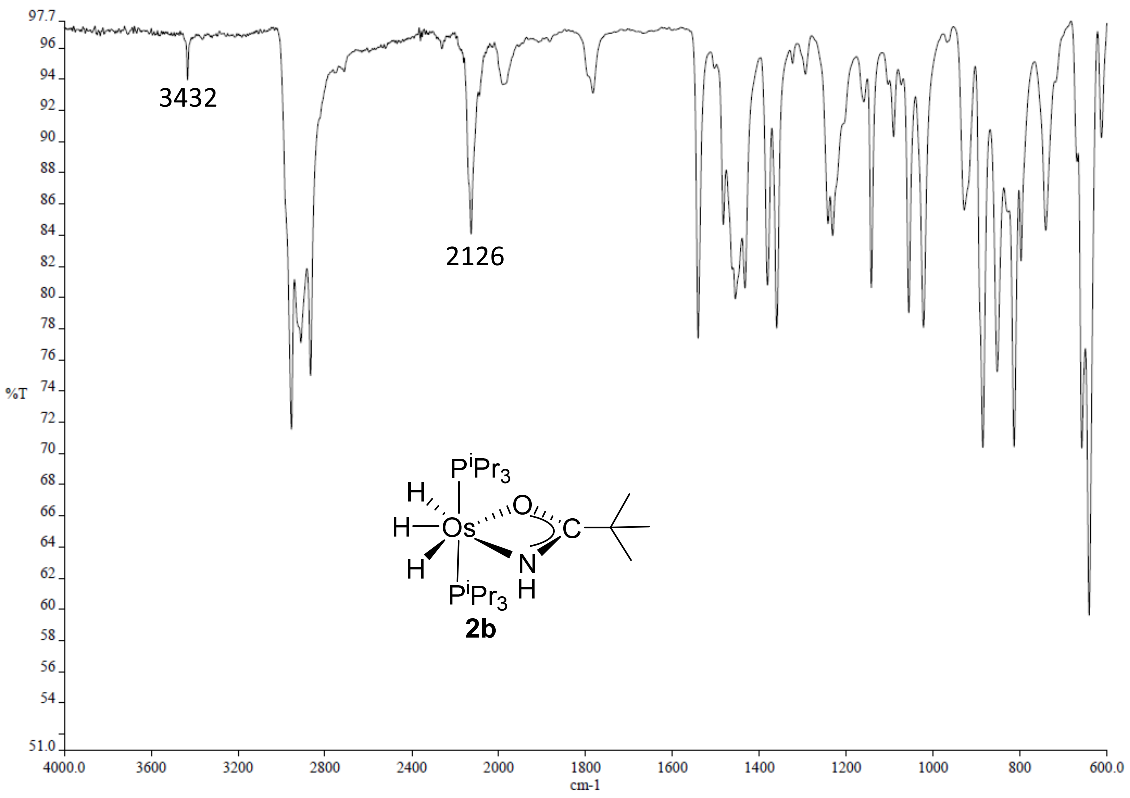

Figure S92. IR ATR spectrum of complex **2b**.

Energy of computed structures

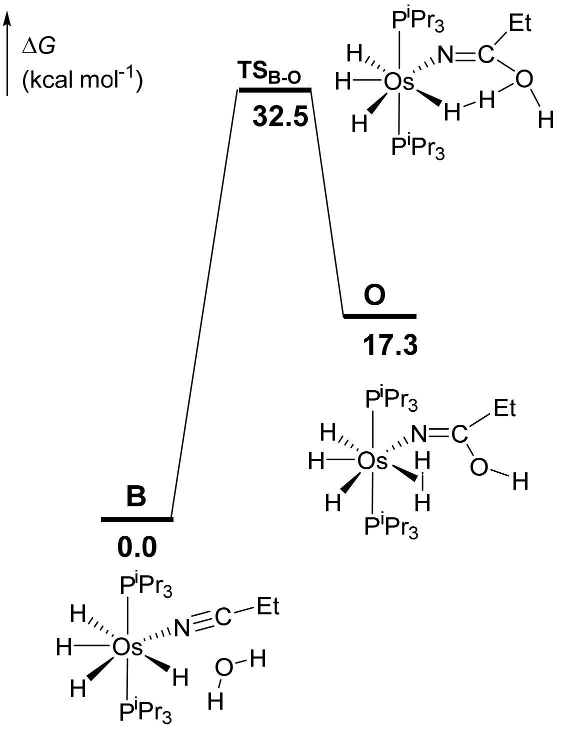

Figure S93. Computed energy profile for the nucleophilic attack of an external water molecule to the coordinated nitrile.

H_2O

Zero-point correction= 0.021181 (Hartree/Particle)
Thermal correction to Energy= 0.024016
Thermal correction to Enthalpy= 0.024960
Thermal correction to Gibbs Free Energy= 0.003516
Sum of electronic and zero-point Energies= -76.404856
Sum of electronic and thermal Energies= -76.402020
Sum of electronic and thermal Enthalpies= -76.401076
Sum of electronic and thermal Free Energies= -76.422520

Propionitrile

Zero-point correction= 0.074232 (Hartree/Particle)
Thermal correction to Energy= 0.078935
Thermal correction to Enthalpy= 0.079879
Thermal correction to Gibbs Free Energy= 0.047479
Sum of electronic and zero-point Energies= -172.013166
Sum of electronic and thermal Energies= -172.008463
Sum of electronic and thermal Enthalpies= -172.007519
Sum of electronic and thermal Free Energies= -172.039919

Propanamide

Zero-point correction= 0.102375 (Hartree/Particle)
Thermal correction to Energy= 0.108641
Thermal correction to Enthalpy= 0.109585
Thermal correction to Gibbs Free Energy= 0.072762
Sum of electronic and zero-point Energies= -248.455963
Sum of electronic and thermal Energies= -248.449697
Sum of electronic and thermal Enthalpies= -248.448753
Sum of electronic and thermal Free Energies= -248.485576

Complex A

Zero-point correction= 0.604451 (Hartree/Particle)
Thermal correction to Energy= 0.637528
Thermal correction to Enthalpy= 0.638472
Thermal correction to Gibbs Free Energy= 0.543242
Sum of electronic and zero-point Energies= -1486.672132
Sum of electronic and thermal Energies= -1486.639056
Sum of electronic and thermal Enthalpies= -1486.638112
Sum of electronic and thermal Free Energies= -1486.733342

Complex B

Zero-point correction= 0.707984 (Hartree/Particle)
Thermal correction to Energy= 0.749424
Thermal correction to Enthalpy= 0.750368
Thermal correction to Gibbs Free Energy= 0.636751
Sum of electronic and zero-point Energies= -1735.137734
Sum of electronic and thermal Energies= -1735.096294

Sum of electronic and thermal Enthalpies= -1735.095350
Sum of electronic and thermal Free Energies= -1735.208966

$TS_{(B-O)}$

Zero-point correction= 0.704794 (Hartree/Particle)
Thermal correction to Energy= 0.744469
Thermal correction to Enthalpy= 0.745413
Thermal correction to Gibbs Free Energy= 0.634687
Sum of electronic and zero-point Energies= -1735.087130
Sum of electronic and thermal Energies= -1735.047455
Sum of electronic and thermal Enthalpies= -1735.046511
Sum of electronic and thermal Free Energies= -1735.157237

Complex O

Zero-point correction= 0.708807 (Hartree/Particle)
Thermal correction to Energy= 0.748478
Thermal correction to Enthalpy= 0.749422
Thermal correction to Gibbs Free Energy= 0.639407
Sum of electronic and zero-point Energies= -1735.112065
Sum of electronic and thermal Energies= -1735.072394
Sum of electronic and thermal Enthalpies= -1735.071450
Sum of electronic and thermal Free Energies= -1735.181465

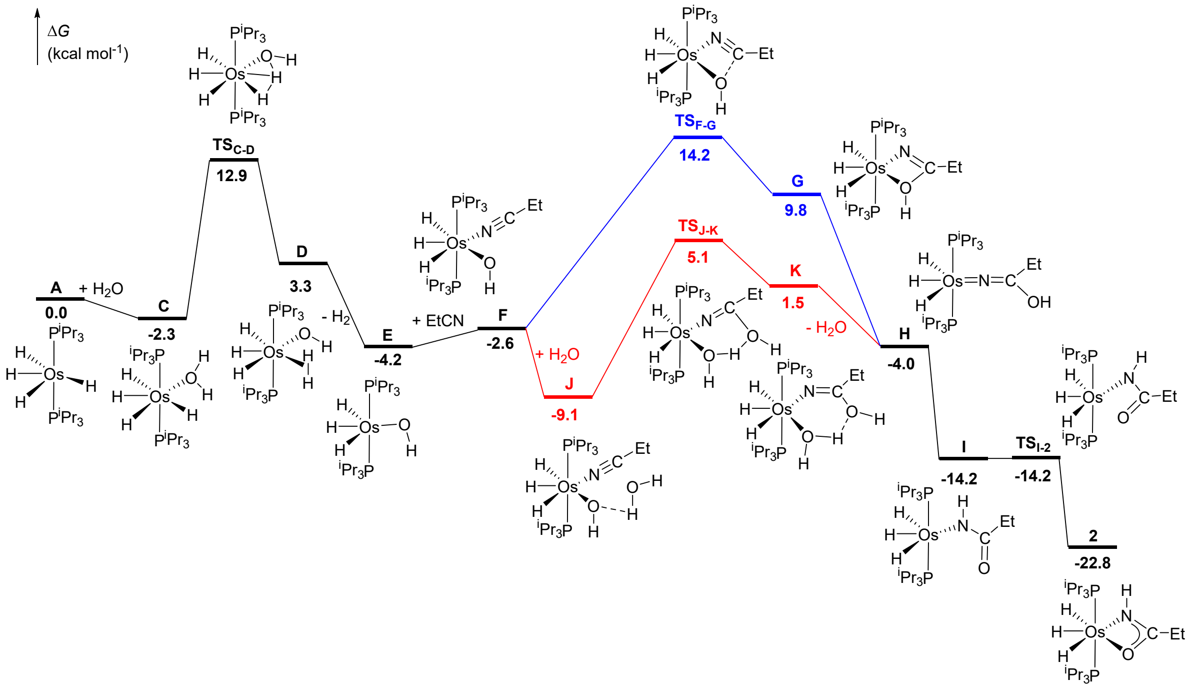

Figure S94. Computed energy profile for the formation of the κ^2-amidate (**2**) via intramolecular attack (blue lines) or intermolecular (red lines).

Complex C

Zero-point correction= 0.632071 (Hartree/Particle)
Thermal correction to Energy= 0.667277
Thermal correction to Enthalpy= 0.668221
Thermal correction to Gibbs Free Energy= 0.568687
Sum of electronic and zero-point Energies= -1563.096114
Sum of electronic and thermal Energies= -1563.060908
Sum of electronic and thermal Enthalpies= -1563.059963
Sum of electronic and thermal Free Energies= -1563.159497

$TS_{(C-D)}$

Zero-point correction= 0.627153 (Hartree/Particle)
Thermal correction to Energy= 0.661729
Thermal correction to Enthalpy= 0.662673
Thermal correction to Gibbs Free Energy= 0.564171
Sum of electronic and zero-point Energies= -1563.072345
Sum of electronic and thermal Energies= -1563.037769
Sum of electronic and thermal Enthalpies= -1563.036825
Sum of electronic and thermal Free Energies= -1563.135327

Complex D

Zero-point correction= 0.630516 (Hartree/Particle)
Thermal correction to Energy= 0.665251
Thermal correction to Enthalpy= 0.666195
Thermal correction to Gibbs Free Energy= 0.567258
Sum of electronic and zero-point Energies= -1563.087402
Sum of electronic and thermal Energies= -1563.052667
Sum of electronic and thermal Enthalpies= -1563.051722
Sum of electronic and thermal Free Energies= -1563.150660

Complex E

Zero-point correction= 0.612561 (Hartree/Particle)
Thermal correction to Energy= 0.647025
Thermal correction to Enthalpy= 0.647969
Thermal correction to Gibbs Free Energy= 0.548415
Sum of electronic and zero-point Energies= -1561.919112
Sum of electronic and thermal Energies= -1561.884647
Sum of electronic and thermal Enthalpies= -1561.883703
Sum of electronic and thermal Free Energies= -1561.983257

Complex F

Zero-point correction= 0.689745 (Hartree/Particle)
Thermal correction to Energy= 0.729812
Thermal correction to Enthalpy= 0.730756
Thermal correction to Gibbs Free Energy= 0.619061
Sum of electronic and zero-point Energies= -1733.949833
Sum of electronic and thermal Energies= -1733.909766
Sum of electronic and thermal Enthalpies= -1733.908822

Sum of electronic and thermal Free Energies= -1734.020518

$TS_{(F-G)}$

Zero-point correction= 0.690416 (Hartree/Particle)
Thermal correction to Energy= 0.728914
Thermal correction to Enthalpy= 0.729859
Thermal correction to Gibbs Free Energy= 0.623741
Sum of electronic and zero-point Energies= -1733.927107
Sum of electronic and thermal Energies= -1733.888609
Sum of electronic and thermal Enthalpies= -1733.887664
Sum of electronic and thermal Free Energies= -1733.993782

Complex G

Zero-point correction= 0.691475 (Hartree/Particle)
Thermal correction to Energy= 0.730098
Thermal correction to Enthalpy= 0.731042
Thermal correction to Gibbs Free Energy= 0.624375
Sum of electronic and zero-point Energies= -1733.933695
Sum of electronic and thermal Energies= -1733.895072
Sum of electronic and thermal Enthalpies= -1733.894128
Sum of electronic and thermal Free Energies= -1734.000795

Complex H

Zero-point correction= 0.691001 (Hartree/Particle)
Thermal correction to Energy= 0.730301
Thermal correction to Enthalpy= 0.731245
Thermal correction to Gibbs Free Energy= 0.621564
Sum of electronic and zero-point Energies= -1733.953422
Sum of electronic and thermal Energies= -1733.914123
Sum of electronic and thermal Enthalpies= -1733.913179
Sum of electronic and thermal Free Energies= -1734.022860

Complex I

Zero-point correction= 0.691994 (Hartree/Particle)
Thermal correction to Energy= 0.731016
Thermal correction to Enthalpy= 0.731960
Thermal correction to Gibbs Free Energy= 0.623217
Sum of electronic and zero-point Energies= -1733.970309
Sum of electronic and thermal Energies= -1733.931288
Sum of electronic and thermal Enthalpies= -1733.930343
Sum of electronic and thermal Free Energies= -1734.039086

$TS_{(I-2)}$

Zero-point correction= 0.690934 (Hartree/Particle)
Thermal correction to Energy= 0.729553
Thermal correction to Enthalpy= 0.730497
Thermal correction to Gibbs Free Energy= 0.622466

Sum of electronic and zero-point Energies= -1733.971329
Sum of electronic and thermal Energies= -1733.932710
Sum of electronic and thermal Enthalpies= -1733.931766
Sum of electronic and thermal Free Energies= -1734.039797

Complex 2

Zero-point correction= 0.692421 (Hartree/Particle)
Thermal correction to Energy= 0.731170
Thermal correction to Enthalpy= 0.732114
Thermal correction to Gibbs Free Energy= 0.623732
Sum of electronic and zero-point Energies= -1733.984059
Sum of electronic and thermal Energies= -1733.945310
Sum of electronic and thermal Enthalpies= -1733.944366
Sum of electronic and thermal Free Energies= -1734.052748

Complex J

Zero-point correction= 0.715173 (Hartree/Particle)
Thermal correction to Energy= 0.757661
Thermal correction to Enthalpy= 0.758605
Thermal correction to Gibbs Free Energy= 0.642706
Sum of electronic and zero-point Energies= -1810.380974
Sum of electronic and thermal Energies= -1810.338485
Sum of electronic and thermal Enthalpies= -1810.337541
Sum of electronic and thermal Free Energies= -1810.453440

$TS_{(J-K)}$

Zero-point correction= 0.713038 (Hartree/Particle)
Thermal correction to Energy= 0.753900
Thermal correction to Enthalpy= 0.754844
Thermal correction to Gibbs Free Energy= 0.643004
Sum of electronic and zero-point Energies= -1810.360754
Sum of electronic and thermal Energies= -1810.319891
Sum of electronic and thermal Enthalpies= -1810.318947
Sum of electronic and thermal Free Energies= -1810.430787

Complex K

Zero-point correction= 0.717594 (Hartree/Particle)
Thermal correction to Energy= 0.758831
Thermal correction to Enthalpy= 0.759775
Thermal correction to Gibbs Free Energy= 0.647294
Sum of electronic and zero-point Energies= -1810.366294
Sum of electronic and thermal Energies= -1810.325057
Sum of electronic and thermal Enthalpies= -1810.324113
Sum of electronic and thermal Free Energies= -1810.436594

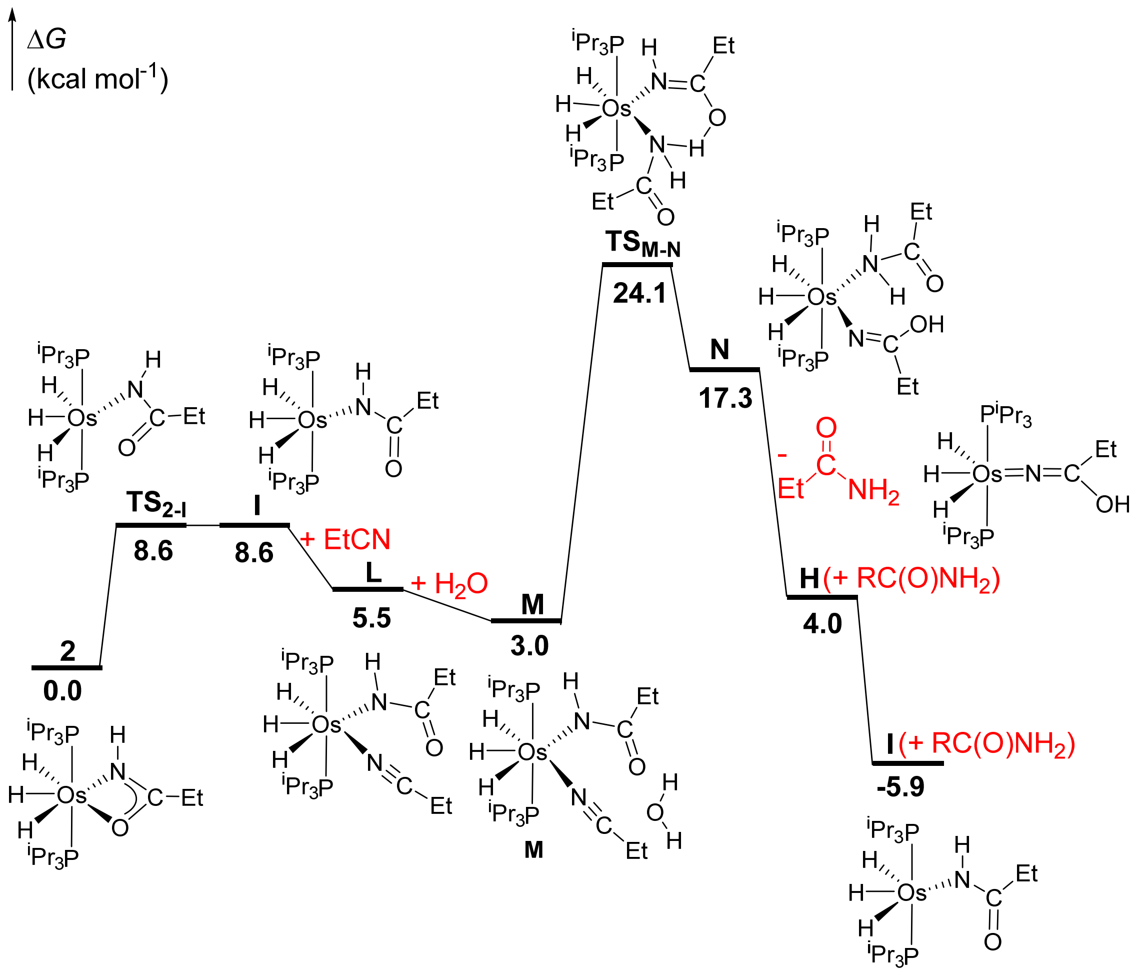

Figure S95. Computed energy profile for the catalytic cycle.

Complex L

Zero-point correction=	0.770076 (Hartree/Particle)
Thermal correction to Energy=	0.814295
Thermal correction to Enthalpy=	0.815240
Thermal correction to Gibbs Free Energy=	0.695408
Sum of electronic and zero-point Energies=	-1906.009235
Sum of electronic and thermal Energies=	-1905.965015
Sum of electronic and thermal Enthalpies=	-1905.964071
Sum of electronic and thermal Free Energies=	-1906.083902

Complex M

Zero-point correction=	0.795501 (Hartree/Particle)
Thermal correction to Energy=	0.842365
Thermal correction to Enthalpy=	0.843309
Thermal correction to Gibbs Free Energy=	0.718773
Sum of electronic and zero-point Energies=	-1982.433678
Sum of electronic and thermal Energies=	-1982.386815
Sum of electronic and thermal Enthalpies=	-1982.385871
Sum of electronic and thermal Free Energies=	-1982.510407

$TS_{(M-N)}$

Zero-point correction= 0.792413 (Hartree/Particle)
Thermal correction to Energy= 0.838008
Thermal correction to Enthalpy= 0.838953
Thermal correction to Gibbs Free Energy= 0.717283
Sum of electronic and zero-point Energies= -1982.401694
Sum of electronic and thermal Energies= -1982.356099
Sum of electronic and thermal Enthalpies= -1982.355155
Sum of electronic and thermal Free Energies= -1982.476824

Complex N

Zero-point correction= 0.798300 (Hartree/Particle)
Thermal correction to Energy= 0.844046
Thermal correction to Enthalpy= 0.844990
Thermal correction to Gibbs Free Energy= 0.723580
Sum of electronic and zero-point Energies= -1982.412995
Sum of electronic and thermal Energies= -1982.367250
Sum of electronic and thermal Enthalpies= -1982.366306
Sum of electronic and thermal Free Energies= -1982.487716

Comparative tables of metal-catalyzed nitrile hydration

Table S1. Transition metal catalyzed hydration of nitriles showing the number of aryl, alkyl and vinyl nitriles used for each catalyst

Catalyst	Aryl Nitriles	Alkyl Nitriles	Vinyl Nitriles	ref[a]
$(MeCp)_2Mo(OH)(H_2O)^+$	2	7	1	6b
[PtCl(PR$_2$OH)-{(PR$_2$O)$_2$H}] and Cp$_2$Mo(OH)(OH$_2$)	0	13	0	11d
$Na_2MoO_4{\cdot}2H_2O$	9	3	0	10
(η^5-C$_9$H$_7$)Ru(dppm)H	3	1	0	12a
cis-Ru(acac)$_2$(PPh$_2$py)$_2$	5	5	3	8a
L$_2$Ru(acac)$_2$ (L = 6-diphenylphosphino-N-pivaloyl-2-aminopyridine or 3-diphenylphosphinoisoquinolone)	1	0	0	8b
[RuCl$_2$(η^6 -C$_6$Me$_6$)(PTA-Bn)] (PTA-Bn=1-benzyl-3,5-diaza-1-azonia-7-phosphaadamantane chloride)	29	11	5	12b
{[(PCy$_3$)(CO)RuH]$_4$(μ^4-O)(μ^3-OH)(μ^2-OH)}	3	7	3	13c
TpRu(PPh$_3$)(H$_2$O)(NHC(O)CH$_3$)	3	7	0	9a
[Ru(H$_2$O)(NCMe)$_4$(P^{i}Pr$_3$)](BF$_4$)$_2$	0	1	0	12c
RuCl$_2$-{κ^1(P)-3-Ph$_2$PC$_6$H$_4$CH$_2$NH^{t}Bu}(η^6-1,3,5-C$_6$H$_3$Me$_3$)	10	3	1	12d
RuCl$_2$(η^3:η^3-C$_{10}$H$_{16}$)(THPA) (THPA = 2,4,10-trimethyl-1,2,4,5,7,10-hexaaza-3-phosphatricyclo[3.3.1.13,7]decane)	16	5	1	12e
RuCl$_2$(η^6-arene)(PR$_3$)	4	4	1	12f
[RuCl$_2$(L)(dmso-S)$_3$] (L = pirazols)	1	0	1	12g
RuCl$_2$(η^6 -C$_6$Me$_6$)-{P(NMe$_2$)$_3$}	21	11	2	6d
Ru(methallyl)$_2$(cod)	8	3	2	12h
RuCl$_2$(PTA)$_4$	11	2	1	12i
RuCl$_2$(η^6-p-cymene){κ^1-(P)-PPh$_2$py}	1	0	0	12j
Ru(η^6-*p*-cymene)Cl$_2$(P(NMe$_2$)$_3$)	0	3	0	12k
Ru(II) catalysts inspired by the metalloenzyme	1	0	0	12l
RuCl$_2$(η^6-p-cymene) + N-protonated thiazolyl-phosphine	19	7	3	12m
Ru(η^6-arene)Cl$_2$(P(NMe$_2$)$_3$)	0	5	0	12n
Ru(η^6-p-cymene)Cl$_2$(P(OH)Me$_2$)	1	6	2	12o
[(η^6-C$_6$H$_5$CH$_3$)RuCl(κ^2-(*P*,*N*)PTA-CPh$_2$NHPh)]Cl	10	2	1	12p
RuCl$_2$(pypz-H)(dmso)$_2$ and RuCl$_2$(pz-H)(dmso)$_3$	5	3	1	12q
[RuOTf{η^6:κ^1(P)-PPh$_2$-binaphthyl}{PPh$_2$(OH)}][OTf]	21	7	1	12r
RuCl$_2$(dmso)$_4$ and [(η^6-*p*-cymene)RuCl$_2$]$_2$	8	8	0	12s
[(η^6-toluene)RuCl$_2$(PTA-P^{i}Pr$_2$)]	5	1	1	12t
RuCl$_2$(L)(dmso-S)$_3$	1	0	1	6h
[RuCl$_2$(η^6-arene){P(OR)$_2$OH}]	1	1	0	12u
[RuCl$_2$(η^6-*p*-cymene){P(4-C$_6$H$_4$F)$_2$Cl}]	16	17	1	12v
[RuCl$_2$(η^3:η^3-C$_{10}$H$_{16}$)(PMe$_2$OH)]	26	7	1	12w
[(Me$_3$P)$_3$Ru(l-OH)$_3$Ru(PMe$_3$)$_3$][OPh]	10	5	1	8c
[RuCl$_2$(η^6-*p*-cymene){P(4-C$_6$H$_4$F)$_2$Cl}	0	31	0	6j
Ru(*p*-cymene)LCl (HL = 2-(5,6- dihydro-4H-1,3-oxazin-2-yl)phenol))	11	0	0	7b
RuCl$_2$(PTA)$_4$	9	1	0	6k
[RuCl$_2$(η^6-arene)PFu$_3$]	29	4	1	6l
RuH(CO)(PNP)	24	7	1	7c
[Os(CO)$_3$Cl$_2$]$_2$	0	1	0	26a
[OsCl$_2$(azole)$_2$(dm*s*o)$_2$] [OsCl$_2$(azole)(dm*s*o)$_3$]	0	5	0	11a
RuII- and OsII-arene	0	3	0	11b
[Os(η^6-*p*-cymene)(OH)IPr]OTf	7	5	0	25
[OsCl$_2$(η^6-*p*-cymene)(PMe$_2$OH)]	20	8	1	26b
[OsCl$_2$(η^6-*p*-cymene){PPh$_2$(NMe$_2$)}]	17	8	0	6h
[Co(cyclen)(OH$_2$)$_2$]$^{3+}$	0	1	0	13b
Rh(COD)(κ-C$_2$-PIN)Br	12	6	3	11e
[RhCl(COD){P(NMe$_2$)$_3$}	17	7	0	6g
RhCl(cod)(IMes)	7	0	0	9f
[Ir(O$_2$)(PMe$_3$)$_4$]Cl	0	1	0	11c
Cp*IrClL	13	3	0	18
(PCP)Ni–OH	5	4	3	9d
NiI(II) N-heterociclic carbenes	15	5	2	13d
(PN$_3$P)Ni(COOH)	3	2	1	9e
trans-PtHCl(PMe$_3$)$_2$	0	1	1	13a
PtH(PMe$_2$OH)(PMe$_2$O)$_2$H	1	2	1	16a

$PtH(PMe_2OH)(PMe_2O)_2H$	1	1	1	16b
$PtH(PMe_2OH)(PMe_2O)_2H$	2	5	0	16c
$(DPPF)PtCl_2$, PMe_2OH, and AgOTf	6	14	0	17
$Pt(COD)Cl_2$	9	2	0	7a
$Pd(OAc)_2/Sc(OTf)_3$	15	3	0	11f
$Cu_4I_4(H_2O)_4$	11	3	1	6e
CuO	13	2	0	6f
$(IPr)Au(NTf_2)$	22	3	3	9b
$[\{Au(NHC)\}_2(\mu\text{-}OH)][BF_4]$	1	0	0	9c
	**524 (60%)**	**296 (34%)**	**53 (6%)**	

[a]The numbers correspond to the references shown in the article.

Table S2. Conditions for de catalytic hydration of acetonitrile with different catalyst.

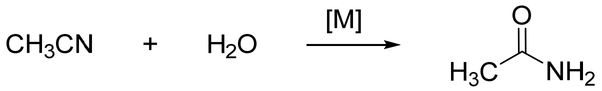

Catalyst	[M] mol %	t (h)	*T* (ºC)	Add.	Yield (%)	ref[a]
$[(MeCp)_2Mo(OH)(H_2O)]^+$	4	9 d	75	–	100	6b
$[Cp_2Mo(OH)(OH_2)]^+$	0.1	45	43	–	100	11d
$(\eta^5\text{-}C_9H_7)Ru(dppm)H$	0.01	72	120	–	86	12a
$TpRu(PPh_3)(H_2O)(NHC(O)CH_3)$	0.5	24	150	–	64	9a
$[Ru(H_2O)(NCMe)_4(P^iPr_3)](BF_4)$	0.01	120	80	KOH	15.2	12c
$RuCl_2(\eta^6\text{-}C_6Me_6)\{P(NMe_2)_3\}$	5	2	100	–	99	6d
$RuCl_2(\eta^6$-p-cymene) + N-protonated thiazolyl-phosphine	3	1	100	–	99	12m
$Ru(\eta^6$-p-cymene)$Cl_2(P(OH)Me_2)$	5	1	100	pH 3.5	98	12o
$[Ru(OTf)\{\eta^6:\kappa^1(P)\text{-}PPh_2$-binaphthyl$\}\{PPh_2(OH)\}][OTf]$	5	5	100	–	99	12r
$[RuCl_2(\eta^6$ -arene$)\{P(OR)_2OH\}]$	1	1	100	–	98	12u
$[RuCl_2(\eta^3:\eta^3\text{-}C_{10}H_{16})(PMe_2OH)]$	1	6	60	–	99	12w
$[(Me_3P)_3Ru(\mu\text{-}OH)_3Ru(PMe_3)_3][OPh]$	2	20	120	–	56	8c
$[RuCl_2(\eta^6$-arene$)PFu_3]$	3	2	80	–	99	6l
RuH(CO)(PNP)	3	24	50	–	96	7c
$[Os(CO)_3Cl_2]_2$	0.3	45	100	–	45	26a
$[Os(\eta^6$-p-cymene)(OH)IPr]OTf	3	6	120	KOH	97	25
$[OsCl_2(\eta^6$-p-pymene$)(PMe_2OH)]$	1	1	80	–	99	26b
$RhCl(COD)\{P(NMe_2)_3\}$	5	24	100	–	5	6g
Cp*IrClL	0.5	6	80	–	96	18
(PCP)Ni–OH	0.1-1	1-4	80	–	32-99	9d
$(PN^3P)Ni(OH)$	1	24	100	–	99	9e
$PtH(PMeOH)(PMe_2O_2)H$	10	15.5	100	–	91	16b
$Cu_4I_4(H_2O)_4$	2.5	21	100	–	63	6e

[a]The numbers correspond to the references shown in the article.

Table S3. Catalytic conditions for hydration of linear chain nitriles

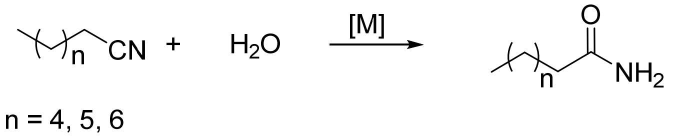

Catalyst	[M] mol %	t (h)	*T* (ºC)	Add.	Yield (%)	ref[a]
Na_2MoO_4–$2H_2O$	10	12	100	acetaldoxime	87	10
cis-Ru(acac)$_2$-(PPh$_2$py)$_2$	0.2	0.5	180	–	93	8a
[RuCl$_2$(η^6-C$_6$Me$_6$)(PTA-Bn)]	5	8	100	–	95	12b
[(Pcy$_3$)(CO)RuH]$_4$(μ^4-O)(μ^3-OH)(μ^2-OH)}	1	12	90	–	78	13c
TpRu(PPh$_3$)(H$_2$O)(NHC(O)CH$_3$)	0.5	24	150	–	31	9a
[RuCl$_2${κ^1(P)-3-Ph$_2$PC$_6$H$_4$CH$_2$NHR}-(η^6-arene)]	5	24	100		55	12d
[{RuCl$_2$(η^3:η^3-C$_{10}$H$_{16}$)}$_2$(THPA)]	5	24	100	–	94	12e
[RuCl$_2$(η^6-C$_6$Me$_6$){P(Nme$_2$)$_3$}]	5	4	100	–	97	6d
[Ru(methallyl)$_2$(cod)] + pyridylphosphine	1	5	80	–	99	12h
RuCl$_2$(PTA)$_4$	5	7	100	–	72	12i
RuCl$_2$(η^6-p-cymene) + N-protonated thiazolyl-phosphine	3	7	100	–	99	12m
[(η^6-C$_6$H$_5$CH$_3$)RuCl(κ^2-(P,N)PTA-CPh$_2$NHPh)]Cl	5	24	100	–	62	12p
[Ru(Otf){η^6:κ^1(P)-PPh$_2$-binaphthyl}{PPh$_2$(OH)}][Otf]	5	6	100	–	99	12r
(η^6-toluene)RuCl$_2$(PTA-P^{i}Pr$_2$)	5	24	100	–	74	12t
RuCl(η^6-p-cymene){P(4-C$_6$H$_4$F)$_2$Cl}	2	6	100	–	99	12v
RuCl$_2$(η^3:η^3-C$_{10}$H$_{16}$)(PMe$_2$OH)	1	6	60	–	97	12w
[RuCl$_2$(PTA)$_4$]	5	24	100	–	99	6k
[RuCl$_2$(η^6-arene)PFu$_3$]	3	2	80	–	99	6l
[OsCl$_2$(η^6-p-cymene)(PMe$_2$OH)]	1	1	80	–	99	26b
[OsCl$_2$(η^6-p-cymene){PPh$_2$(NMe$_2$)}]	1	4	100	–	99	6i
RhCl(COD){P(NMe$_2$)$_3$}	5	24	100	–	12	6g
[Rh(COD)(κ-C$_2$-PIN)Br]	0.5	6	25	^{t}BuOK	75	11e
Pd(OAc)$_2$/Sc(OTf)$_3$	5/10	12	70	AcOH	67	11f
CuO	10	12	100	acetaldoxime	90	6f

[a]The numbers correspond to the references shown in the article.

Table S4. Catalytic conditions for hydration of branched chain nitriles

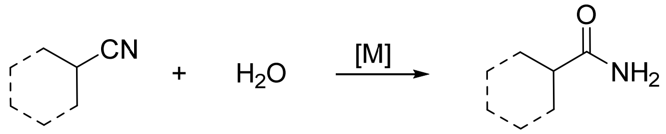

Catalyst	[M] mol %	t (h)	*T* (ºC)	Add.	Yield (%)	ref[a]
[(MeCp)$_2$Mo(OH)(H$_2$O)]$^+$	4	13 d	85	–	100	6b
[RuCl$_2$(η^6-C$_6$Me$_6$)(PTA-Bn)]	5	5	100	–	98	12b
[(Pcy$_3$)(CO)RuH]$_4$(μ^4-O)(μ^3-OH)(μ^2-OH)}	1	12	90	–	84	13c
TpRu(PPh$_3$)(H$_2$O)(NHC(O)CH$_3$)	0.5	24	150	–	50	9a
[{RuCl$_2$(η^3: η^3-C$_{10}$H$_{16}$)}$_2$(THPA)]	5	24	100	–	83	12e
Ru(η^6-p-cymene)Cl$_2$(P(OH)Me$_2$)	5	7.5	100	pH 3.5	74	12o
[Ru(OTf){ η^6:κ^1(P)-PPh$_2$-binaphthyl}{PPh$_2$(OH)}][OTf]	5	24	100	–	91	12r
RuCl$_2$(dmso)$_4$ and [(η^6-*p*-cymene)RuCl$_2$]$_2$	5	1	100	(pta-Bn)Cl	97	12s
RuH(CO)(PNP)	3	24	50	–	99	7c
[Os(η^6-p-cymene)(OH)IPr]OTf	3	16	120	KOH	97	25
[OsCl$_2$(η^6-p-cymene)(PMe$_2$OH)]	1	1.5	80	–	99	26b
[OsCl$_2$(η^6-p-cymene){PPh$_2$(NMe$_2$)}]	1	2.5	100	–	99	6i
Cp*IrClL	0.5	6	80	–	95	18
(PCP)Ni–OH	0.1-1.0	1-4	80	–	47-99	9d
(IPr)Au(NTf$_2$)	5	6	140	MW	70	9b

[a]The numbers correspond to the references shown in the article.

Table S5. Catalytic conditions for hydration of pivalonitrile

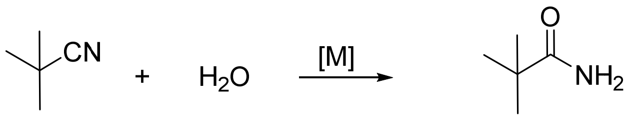

Catalyst	[M] mol %	t (h)	*T* (°C)	Add.	Yield (%)	ref[a]
cis-Ru(acac)$_2$-(PPh$_2$py)$_2$	0.2	0.5	180	–	75	8a
RuCl$_2$(PTA)$_4$	5	7	100	–	67	12i
Ru(η^6-p-cymene)Cl$_2$(P(OH)Me$_2$)	5	7.5	100	pH 3.5	90	12o
[(η^6-C$_6$H$_5$CH$_3$)RuCl(κ^2-(*P*,*N*)PTA-CPh$_2$NHPh)]Cl	5	24	100	–	79	12p
RuH(CO)(PNP)	3	24	50	–	96	7c
[Rh(COD)(κ-C$_2$-PIN)Br]	0.5	24	25	^{t}BuOK	38	11e
Cp*IrClL	0.5	16	80	–	97	18
(PCP)Ni–OH	0.5-1.0	6-8	80	–	95	9d

[a]The numbers correspond to the references shown in the article.
